# Stochastic models support rapid peopling of Late Pleistocene Sahul

**DOI:** 10.1038/s41467-021-21551-3

**Published:** 2021-04-29

**Authors:** Corey J. A. Bradshaw, Kasih Norman, Sean Ulm, Alan N. Williams, Chris Clarkson, Joël Chadœuf, Sam C. Lin, Zenobia Jacobs, Richard G. Roberts, Michael I. Bird, Laura S. Weyrich, Simon G. Haberle, Sue O’Connor, Bastien Llamas, Tim J. Cohen, Tobias Friedrich, Peter Veth, Matthew Leavesley, Frédérik Saltré

**Affiliations:** 1 Global Ecology, College of Science and Engineering, Flinders University, Adelaide, SA Australia; 2 ARC Centre of Excellence for Australian Biodiversity and Heritage, Wollongong, NSW Australia; 3 Centre for Archaeological Science, School of Earth, Atmospheric and Life Sciences, University of Wollongong, Wollongong, NSW Australia; 4 College of Arts, Society and Education, James Cook University, Cairns, QLD Australia; 5 Climate Change Research Centre, School of Biological, Earth and Environmental Sciences, University of New South Wales, Sydney, NSW Australia; 6 EMM Consulting, St Leonards, NSW Australia; 7 School of Social Science, University of Queensland, Brisbane, QLD Australia; 8 Max Planck Institute for the Science of Human History, Jena, Germany; 9 Faculty of Science, Medicine and Health, University of Wollongong, Wollongong, NSW Australia; 10 UR 1052, French National Institute for Agricultural Research (INRA), Montfavet, France; 11 College of Science and Engineering, James Cook University, Cairns, QLD Australia; 12 Department of Anthropology, Pennsylvania State University, University Park, PA USA; 13 Department of Archaeology and Natural History, School of Culture, History and Language, Australian National University, Canberra, ACT Australia; 14 School of Biological Sciences, Environment Institute, University of Adelaide, Adelaide, SA Australia; 15 National Centre for Indigenous Genomics, Australian National University, Canberra, ACT Australia; 16 Department of Oceanography, School of Ocean and Earth Science and Technology, University of Hawai’i at Manoa, Honolulu, Hawai’i USA; 17 Archaeology and the Centre for Rock Art Research and Management M257, School of Social Sciences, University of Western Australia, Crawley, WA Australia; 18 Department of Anthropology and Sociology, University of Papua New Guinea, Port Moresby, Papua New Guinea

**Keywords:** Ecology, Ecological modelling, Geography

## Abstract

The peopling of Sahul (the combined continent of Australia and New Guinea) represents the earliest continental migration and settlement event of solely anatomically modern humans, but its patterns and ecological drivers remain largely conceptual in the current literature. We present an advanced stochastic-ecological model to test the relative support for scenarios describing where and when the first humans entered Sahul, and their most probable routes of early settlement. The model supports a dominant entry via the northwest Sahul Shelf first, potentially followed by a second entry through New Guinea, with initial entry most consistent with 50,000 or 75,000 years ago based on comparison with bias-corrected archaeological map layers. The model’s emergent properties predict that peopling of the entire continent occurred rapidly across all ecological environments within 156–208 human generations (4368–5599 years) and at a plausible rate of 0.71–0.92 km year^−1^. More broadly, our methods and approaches can readily inform other global migration debates, with results supporting an exit of anatomically modern humans from Africa 63,000–90,000 years ago, and the peopling of Eurasia in as little as 12,000–15,000 years via inland routes.

## Abstract (web summary)

Advanced ecological modelling reveals how Sahul (Australia and New Guinea) was first peopled, suggesting the most probable routes and surprisingly rapid early settlement of this continent by anatomically modern humans starting 50,000 to 75,000 years ago.

## Introduction

Around 65,000–50,000 years ago (or earlier^1^), large^2^ and apparently well-organized groups^3^ of anatomically modern humans first entered the continent of Sahul—the landmass connecting New Guinea, some small islands of modern-day Indonesia, mainland Australia and Tasmania at lower sea levels than today. However, the exact timing of initial peopling remains controversial^4,5^ because of arid Sahul’s poor preservation environment and the relatively few reliable archaeological sequences compared to other regions of the world. Nonetheless, models are converging on the idea that first entry into New Guinea through Sulawesi was more likely^3,6,7^, although a southern route through Bali, Timor and onto the now-drowned Sahul Shelf in the Arafura Sea north of the modern-day Kimberley region in Western Australia is also plausible (or possibly both)^3,8–10^.

To date, the most likely migration routes and overall continental pathways of initial peopling have only been speculated from distant point estimates of genetic distance^11–13^, inferred biogeographic connectivity^14,15^ or sparse archaeological data^16–20^. Further, the movements of people through New Guinea have been largely ignored, even though the region was directly connected to northern Australia throughout the hypothesized period of initial peopling^2,3^. Recent genetic evidence from Aboriginal Australians and Papuans^11,12,21–23^ suggests that these peoples originated from a common ancestral population^11,13,21^, eventually diverging into their respective genetic clusters after an initial bottleneck^11^, with little evidence for subsequent gene flow^12,13,22,23^. While genomic evidence proposes that the peopling of Sahul was the result of a single migration event^11^, mitochondrial DNA evidence concludes that settlement occurred via both the southern and northern routes^13,23^. Subsequent gene flow apparently occurred predominately along the coasts of Australia^11,12^, with the arid interior acting as a barrier to movement^11^.

Regardless of the true timing(s) and point(s) of initial entry into Sahul, there is little ecological insight into how these first wave(s) of humans moved across and established populations in the rest of the continent. There is evidence that hunter–gatherer use of space is structured by ecological factors^24–27^. For instance, the average distance of residential mobility is a direct function of the available energy in the local environment^28–30^. These relationships reflect fundamental features in human land use, regardless of the physical or cultural environment, and are thus useful for constructing generalized predictions of past human dispersal at broad spatial scales to test against archaeological data.

We constructed a fully stochastic, cellular-automaton model at a resolution of 0.5° × 0.5° to quantify how the patterns of peopling of Sahul could have occurred based on measured ecological mechanisms. Stochastic, complex-system models like the one we present here are useful not only for forecasting phenomena like global and regional climate patterns^31^, biodiversity extinctions^32^ and land use^33^, but also for hindcasting past phenomena based on hypothesized, but hitherto untested mechanisms^34,35^. Our model and analyses incorporate data-based human-population dynamics to project cell-specific population densities relative to hindcasted^35^, climate-dependent^36^ predictions of maximum carrying capacity derived from established relationships between hunter–gatherer densities and environmental conditions^37^, data-based rules of migration between neighbouring cells, measured long-distance dispersal^38–41^ contingent on water availability^15^ and landscape ruggedness^42^ (a proxy for accessibility), as well as spatially clustered catastrophic mortality events operating at a generational scale based on data from across vertebrate taxa^43^.

We ran a series of 120 scenarios of the model modifying the dates and places of initial entry into Sahul from the islands of Wallacea^3^, as well as the hypothesized relationship between hindcasted net primary production (available biomass to consumers in an ecosystem) and maximum human carrying capacity. To rank the relative realism^44^ of which timings of initial entry, point(s) of initial entry and carrying capacity scenarios were best supported by the available archaeological data, we designed an analysis of the oldest-available, quality-rated archaeological dates to generate a map of initial arrival across the continent corrected for spatial bias and temporal uncertainty. We calculated the correspondence between this bias-corrected map and each of the 120 modelled scenarios to rank their relative likelihood given the archaeological data.

Our model provides a baseline approximation for the relative timing and spread of human dispersal in Sahul on the basis of ecological parameters alone. The reliance here on ecological principles explicitly does not mean that human dispersal in Sahul did not involve deliberate social, cultural and/or economic decisions. Rather, the movement parameters we used were derived from a large global dataset of contemporary and recent hunter–gatherer populations, whose behaviours and decision-making processes were embedded with cultural knowledge and practices. The stochastic nature of the model allows the effect of cultural adaptation on human movement, as captured in the ethnographic dataset, to be expressed probabilistically as part of the modelled human–environment relationship.

Here we show that full peopling of the entire continent of Sahul could have occurred within as few as 150–200 human generations (<5000 years), with up to 6.5 million people potentially living on the continent at saturation (New Guinea: ~1 million and Australia: ~5.5 million). Compared to the mapped archaeological layer, we find most relative support for an initial entry from Wallacea via the southern route onto the now-drowned Sahul Shelf, with a possible second entry later in western New Guinea. The results support both entry times of 50 or 75 ka (1000 years ago = ka), and a non-linear (rotated parabolic) relationship between human carrying capacity and net primary production as most likely of the three variants we test.

## Results

**Fig. 1:**
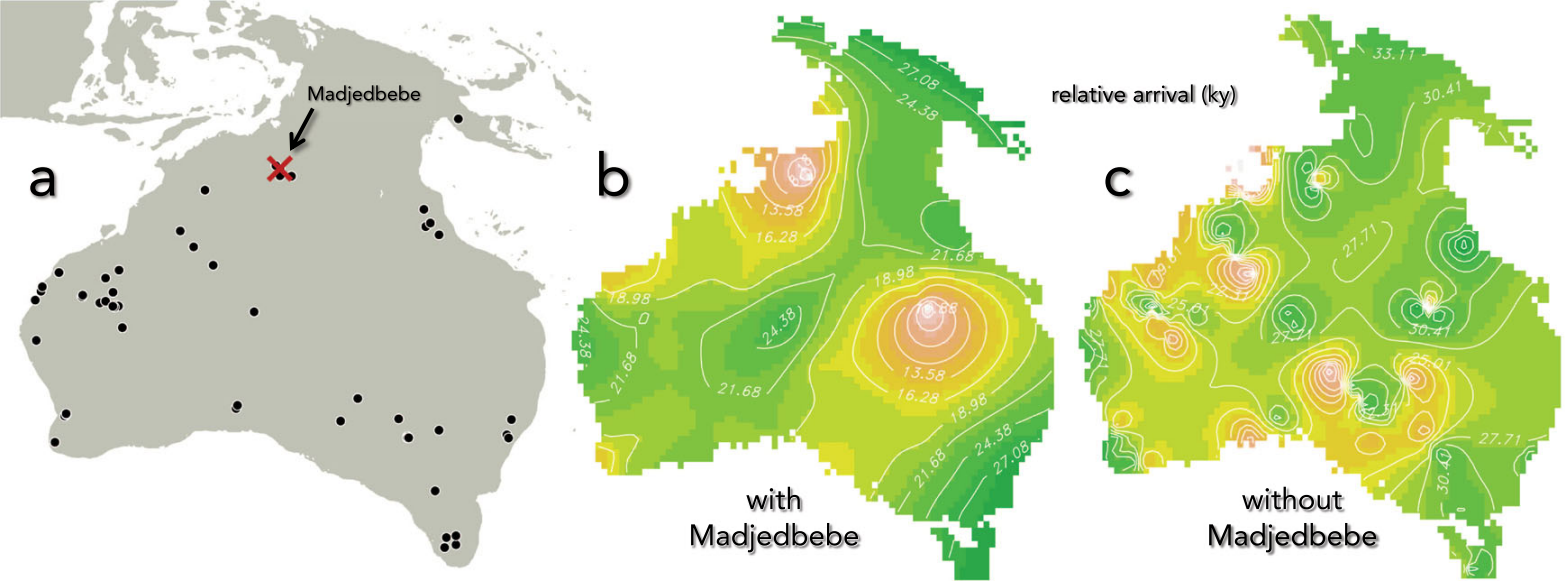
Spatial reconstructions of oldest archaeological dates. **a** Distribution of reliable (A* and A quality-rated) archaeological dates older than 30,000 years used to generate **b** a Signor–Lipps- and spatial-bias-corrected map layer of first arrival (see details in ‘Methods’); in **c** we removed the disputed^1,5,45–48,95^ Madjedbebe dates (position indicated in **a** by a red cross) and reconstructed the layer. All date isohyets in **b** and **c** are expressed in 1000-year (ky) increments relative to arrival time and are based on 100 simulated replicates of the spatial-inference algorithm. Colours indicate progressively earlier (pink) to later (green) archaeologically derived dates. See ‘Methods’ (Cellular-automaton framework) for the source of the map extent for Sahul.

**Fig. 2:**
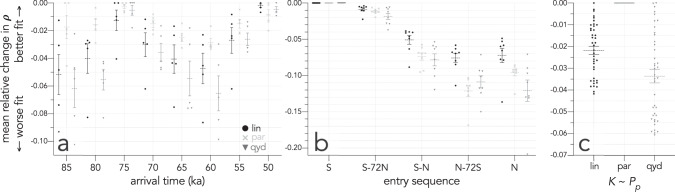
Correlation differences between first-arrival scenarios and archaeological dates. Relative reduction in Spearman’s *ρ* rank correlation between the predicted time of first arrival over all grid cells for each scenario and the Signor–Lipps- and spatial-bias-corrected archaeological map layer. Holding all other input parameters equal, the mean relative change in average *ρ* is expressed as a function of modifying **a** arrival time (from 85 to 50 ka, in 5000-year increments; five scenarios for each increment), **b** entry-point sequence (S = southern route through the Sahul Shelf; N = northern route through Bird’s Head of New Guinea, and combinations of these with lags expressed in terms of human generations: 72 generations ~2000 years; 8 scenarios each), and **c** assumed relationship between human carrying capacity (*K*) and hindcasted net primary production (*P*_*p*_) (lin = linear; par = rotated parabolic; qyd = reciprocal quadratic yield density—see details in ‘Methods’; three scenarios each). The horizontal bars represent the mean relative change in *ρ* and the error bars represent standard errors of the mean.

To estimate the most likely migration routes and overall continental pathways of initial peopling of Sahul, we ran 120 different scenarios (see details in 'Methods') where we varied entry time in 5000-year increments between the plausible limits of 85 and 50 ka, number of entries (single/dual), entry location (following a dominant northern and/or southern route) and sequence (northern–southern or southern–northern), and the relationship between human carrying capacity and net primary production (linear, parabolic or quadratic yield density; see details in ‘Methods’). By comparing our simulated predictions to the spatial bias- and Signor–Lipps-corrected map layer of timing of first arrival derived from the most-reliable (quality-rated) and currently available archaeological data (Fig. 1; see also Supplementary Information and Supplementary Data 1), the following characteristics emerged:

First, of the eight potential arrival times we considered, there was near-equal support (mean relative change in Spearman’s *ρ* compared to the top-ranked model = −0.005, Fig. 2a) for an entry time at 50 ka compared to 75 ka (mean relative change in Spearman’s *ρ* compared to the top-ranked model = −0.007; Fig. 2a). For the procedure to determine the correlation between modelled and archaeological estimates of initial arrival, see ‘Spatial correlation between modelled and archaeologically derived dates of initial arrival’ in the Supplementary Information. The fits derived from these two entry times arise because the older projected dates for the northwest of Sahul near the now-drowned Sahul Shelf entry corresponded closer to the 75-ka date, whereas the distribution of dates in the southeast of Sahul (modern-day Victoria and New South Wales) best aligned with entry at 50 ka (Fig. 1).

In terms of entry sequence, there was clearly more support (mean relative change in Spearman’s *ρ* compared to the top-ranked model = 0.000; Fig. 2b) for a single entry point at the Sahul Shelf offshore from the modern-day Kimberley region of Western Australia (southern entry), with slightly less support (mean relative change in Spearman’s *ρ* compared to the top-ranked model = −0.013, Fig. 2b) for a subsequent entry at western Bird’s Head (Vogelkop Peninsula) in modern-day western New Guinea (i.e., a single southern entry is most supported, followed by a dual entry first in the south and a second entry in the north). In contrast, all scenarios where the northern entry preceded a southern entry, or when there was only a northern entry, always provided worse fits to the primary archaeological map layer (Fig. 2b, Supplementary Fig. 1 and Supplementary Data 1).

Holding all parameters constant while only modifying the assumed relationship between human carrying capacity (*K*) and net primary production (*P*_*p*_), we found that the rotated parabolic relationship (Fig. 2c and Supplementary Fig. 2c, f) gave the best fit between predicted settlement patterns and the primary archaeological map layer, followed by the linear (Fig. 2c and Supplementary Fig. 2b, e) and the quadratic yield density relationships (Fig. 2c and Supplementary Fig. 2d, g). However, both the linear and the rotated parabolic relationships appeared to overemphasize extreme values of *K* across the landscape (Supplementary Fig. 2b, c, e, f). When we repeated the analysis excluding the Madjedbebe site^1,4,5,45–48^ from the derived archaeological map layer (Fig. 1c), the overall fits were much weaker (Supplementary Figs. 3 and 4). While the results using this layer were inconclusive with respect to entry time (Supplementary Fig. 3a), there remained more support for a dominant southern entry (Supplementary Fig. 3b) and the rotated parabolic relationship between *K* and *P*_*p*_ (Supplementary Fig. 3c).

**Fig. 3:**
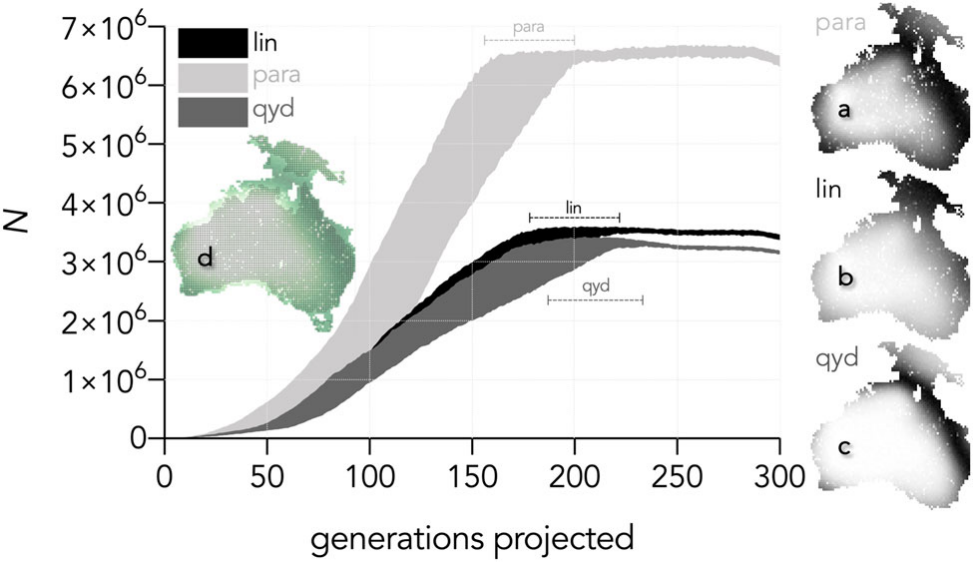
Projected population size according to three assumed relationships between human carrying capacity (*K*) and net primary production (*P*_*p*_). In all three curves, we applied top-ranked scenario settings (50-ka entry at the southern entry-point), but altered the underlying carrying capacity–net primary production relationship. Shaded areas represent 95% confidence intervals based on 100 stochastic simulations. The best-supported relationship was for the rotated parabolic (**a**. para; see also Supplementary Fig. 2), followed by the linear (**b**. lin) and then the quadratic yield density (**c**. qyd). The rotated parabolic resulted in a final population size of 6.31–6.51 million people at 300 generations, or an average population density of 0.55–0.57 people km^−2^. The linear model projected a total continental population of 3.37–3.46 million inhabitants at 300 generations (an average population density of 0.29–0.30 people km^−2^ over the entire continent), and the quadratic yield density projected a population of 3.13–3.19 million (density = 0.27–0.28 people km^−2^). These values are considered high compared with existing archaeological data (see ‘Discussion’), but provide an indication and trends of the populations during the initial peopling of the continent. Inset **d** shows the extent of Sahul overlaid with the modern grid for Australian and New Guinea. This reveals that 27.5% of the population estimate is attributed to now-drowned parts of the continental shelf. Further, the horizontal bars indicate the range of generations required to reach saturation of Sahul for each *K*–*P*_*p*_ relationship. The maps on the right show the relative population distribution at 300 generations for a single iteration following the three different *K*–*P*_*p*_ relationships. See ‘Methods’ (cellular-automaton framework) for the source of the map extent for Sahul.

Based on the projection with a 50-ka southern entry, but varying the underlying relationship between carrying capacity and net primary production, we find that the best-supported rotated parabolic relationship projected a total population of 6.3–6.5 million (0.55–0.57 people km^−2^) within 300 generations (New Guinea: ~0.93–0.96 million; Australia: 5.4–5.5 million), compared to ~3.1–3.5 million inhabitants (0.27–0.30 people km^−2^) using the other (linear or quadratic yield density) *K–P*_*p*_ relationships (Fig. 3). These values are considerably greater than previous estimates^49^, and we provide several caveats regarding the interpretation of this result in ‘Discussion’. The rotated parabolic model (Supplementary Fig. 2f) predicted the highest densities in a general band from northern Australia, east through what is today the Gulf of Carpentaria, up into southeast New Guinea, and down the eastern coastal/inland region of Australia to modern-day New South Wales. Smaller pockets of predicted high carrying capacity occur along the western coast in the Kimberley, and farther south in south–western Western Australia, as well as in Tasmania. These are all areas with ample archaeological evidence of use by humans as refuges before and during the Last Glacial Maximum at ~21 ka^50^.

According to the top-ranked parameters for the different scenarios we considered, full occupation of Sahul occurred between 156 and 200 generations (4368–5599 years) after initial (southern) entry at 50 ka, or between 169 and 208 generations (4732–5796 years) after initial (southern) entry at 75 ka followed by a second northern entry at 73 ka (both using a rotated parabolic relationship between *K* and *P*_*p*_). If we take a direct line from the Sahul Shelf entry-point to the south–eastern coast of mainland Australia north of (but excluding) Tasmania (50-ka scenario), or to the south-eastern tip of Tasmania (75-ka scenario), this implies a maximum rate of progression for regional occupation of 0.71–0.92 km year^−1^.

**Fig. 4:**
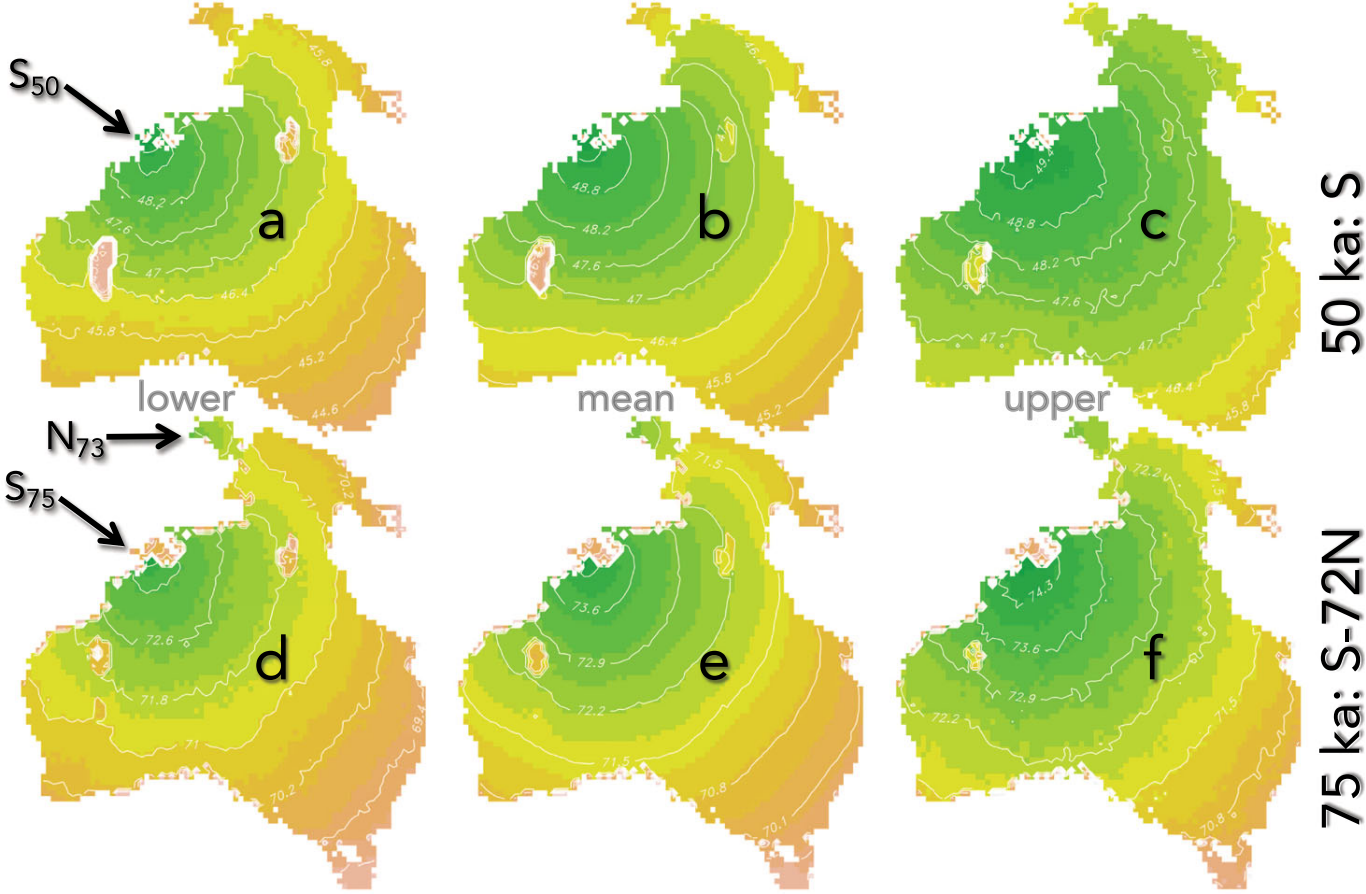
Pattern of first arrival for the two top-ranked modelled scenarios. Here, entry is via the southern route (entry points denoted by arrow in **a**) at 50 ka (S_50_; top row, **a**–**c**; ‘50 ka: S’), and an initial entry via the southern route at 75 ka (S_75_) followed by a second entry via the northern route 72 generations (~2000 years) later (N_73_; bottom row, **d**–**f**; ‘75 ka; S-72N’). Shown are the lower- and upper-percentile (95% confidence interval; **a**, **c** and **d**, **f**) and mean maps (**b** and **e**) for each scenario based on 100 runs of each model. Note in the 50-ka scenario (top row, **a**–**c**), Tasmania was not peopled within the modelled time frame (300 generations) because Bass Strait was flooded during this period (position of Tasmania shown in bottom row). Colours indicate progressively older (green) to younger (pink) dates. All date isohyets are expressed as ky relative to arrival time. See ‘Methods’ (cellular-automaton framework) for the source of the map extent for Sahul.

The general pattern of spread in these scenarios followed an eastward expansion from the southern entry-point from the Sahul Shelf (adjacent to the modern-day Kimberley coast), and then down both the west coast and south- east through east-central Australia (Fig. 4). The eastward expansion across inland Sahul follows the rotated-parabolic model’s predicted regions of highest density, suggesting early inland expansion through the Gulf Country and onto the eastern side of the continent. In both the single, southern entry- and dual-entry scenarios, early movement occurred from the Sahul Shelf northeast into New Guinea (Fig. 4). In the dual-entry scenario (75 ka at the Sahul Shelf followed by 73 ka at Bird’s Head), dispersal northeast from the Sahul Shelf and east from Bird’s Head reached the main body of New Guinea at approximately the same time.

There was a notable gap in the peopling of the arid interior of Australia where the Gibson and Great Victoria Deserts are found today, which is imposed by the limitation of long-distance dispersal and by the water-availability dispersal and occupation-limitation functions (see details in ‘Methods’). This is consistent with the genetic evidence concluding the same phenomenon of extensive arid landscapes restricting settlement^11^. The presence of the inland deserts resulted in population divergence to either side of the arid interior. Most movement was eastward (as stated above), with a smaller dispersal south from the Kimberley, which followed a narrower dispersal path between the coastline and the arid zone. The spread continued southeast toward Tasmania in the 75-ka scenario (Fig. 4d–f). It is also notable that due to submergence of the land bridge between Tasmania and mainland Australia between 62 and 46 ka^51^, scenarios with an initial entry at 50 ka did not result in the peopling of Tasmania during this initial phase (Fig. 4a–c). This phenomenon arises because we assumed only terrestrial progression of the migration wave, whereas sufficiently advanced watercraft technology^3^ could have brought the first people to Tasmania across the intermittently flooded Bass Strait^51^. We note, however, that there is no evidence of voyaging between Tasmania and the mainland since the flooding of Bass Strait ~12 ka^52,53^.

**Fig. 5:**
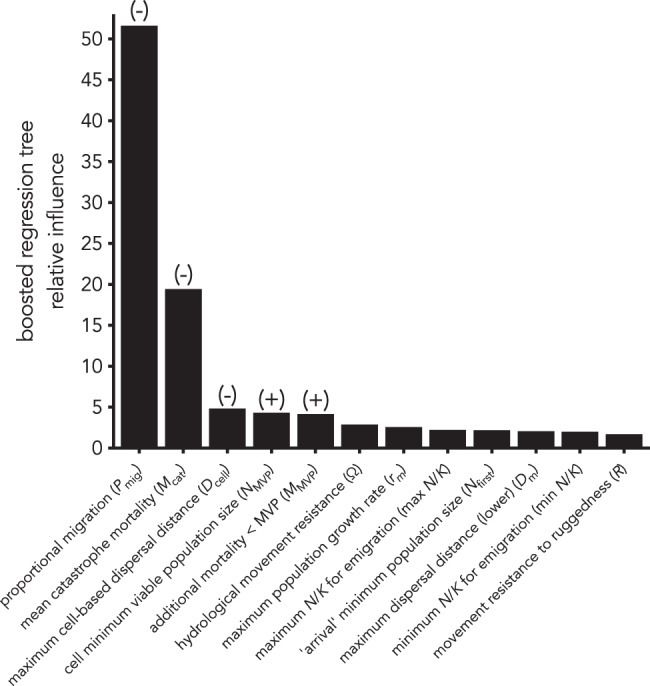
Global sensitivity analysis results. Shown are relative inference scores (summing to 100 across all parameters considered) from a boosted-regression tree^93^ of the relative importance of the model parameter ranges on the time taken to reach continental saturation. See main text for parameter descriptions and ranges tested. The most influential parameters (five top-ranked) are also given with the direction of their influence on the timing of continental saturation: (−) = negative, (+) = positive. Settings for the boosting regression tree were: error distribution = Gaussian, bag fraction = 0.75, learning rate = 0.008, tolerance = 0.0001, maximum number of trees = 10,000, and tree complexity = 2. See also Supplementary Fig. 5.

Testing the model sensitivity to variation in its parameters^54^, the boosted-regression tree emulator (explaining 97.8% of the deviance) for the twelve-dimension, Latin hypercube-sampled parameter space indicated that only two parameters had reasonable influence on the time to saturation of the continent (Fig. 5): (i) variation in the proportion of the population migrating between cells when an inter-cell movement occurred (*P*_mig_)—a 50% decrease in this proportion resulted in a 21% reduction in the time to continental saturation (Fig. 5 and Supplementary Fig. 5a), and (ii) the negative effect of the mean mortality occurring during a catastrophe event (*M*_cat_)—a 50% decrease in this value resulted in a 17% slowing of the time to continental saturation (Supplementary Fig. 5b). Three other variables had a weak effect on time to saturation (Fig. 5): (iii) the cell-based maximum dispersal distance modifier (*D*_cell_), (iv) cell minimum viable population size (*N*_MVP_) and (v) additional mortality <MVP (*M*_MVP_). Increasing the cell-based maximum dispersal distance (*D*_cell_) by five times resulted in a 5% faster time to continental saturation (Supplementary Fig. 5c), whereas a 50% increase in *N*_MVP_ resulted in a 2% slowing of the time to saturation (Supplementary Information Fig. 5d). Likewise, a 50% increase in the additional mortality below MVP (*M*_MVP_) slowed saturation time by ~4% (Supplementary Fig. 5e). All other parameters considered had substantially weaker influence on the rate of progression of the peopling wave (Fig. 5).

As another example of the relative insensitivity of the time to continental saturation by even large changes in maximum dispersal distance, we re-ran 100 iterations of the top-ranked scenario (southern entry only at 50 ka) with *D*_cell_ increased to five times the value in the original model. This only changed the estimated time to saturation to 153–185 generations (4285–5152 years) compared to 156–200 generations predicted from the original model.

## Discussion

One of the most revealing insights provided by our stochastic model is that the rapid peopling of the entire continent of Sahul occurred potentially within as few as 4370–5600 years, across a wide range of environments, including rainforests, savannas, deserts, alpine regions, grasslands and temperate forests. This result was largely insensitive to assumptions regarding landscape carrying capacity (minimum and maximum ratios of *N*/*K* per cell invoking an emigration, and maximum population growth) (Fig. 5). Based on the ecological and anthropological mechanisms we applied, the emergent pattern of spread across Sahul was at least partially congruent with the overall pattern of first arrival derived from the bias-corrected primary archaeological map layer. Rapid expansion across Sahul has been hypothesized previously^12,19^, whereas our model provides the first robust test that such a swift saturation (at least from the perspective of the time people first entered Sahul) was biologically and ecologically feasible. Relying solely on archaeological or genetic data would be unlikely to identify, or be able to test, such rapid rates of human migration given the scarcity of reliable data at the continental scale during the period of initial peopling. The complexity of this fully stochastic model also ensures that no single component—measured or otherwise assumed—can dominate the outcomes of any scenario run. The emergent property of the many interacting and stochastically sampled functions and parameters provide the net spatial patterns of peopling and the plausible pace of this process. No single function within the model could achieve the same outcomes.

The emergent pattern of occupation revealed by our stochastic model is also largely consistent with the available genetic and archaeological evidence, especially with respect to the dominant pathways suggested for Sahul’s western and eastern regions^55^, rather than through the dry interior. Indeed, our hydrologically limited dispersal function effectively supported the genetic view that the dry interior was at least partially a barrier to movement and permanent settlement for the earliest human occupants^11,12^, even though the realized value of the parameter itself had little effect on the rate of progression of the continental migration wave (Fig. 5). Where our model diverges, however, is that the movements southward were not necessarily coastal exclusively, especially within the central and eastern margins of the continent. Indeed, the hindcasted carrying capacity model suggests that expansive areas east of the western deserts were entirely amenable to migration and settlement according to the data-based functions applied in the model. Conversely, the extensive distribution of the arid lands westward (beyond even the modern-day extents of the western deserts) during most of the plausible period of initial settlement pushed the western movement corridor close to the west coast of Sahul (Fig. 4). It is important to be aware that our model predicts migration in terms of settlement patterns as opposed to temporary forays that do not result in local settlement. Second, while a genetic study^11^ found evidence only for southward movement from New Guinea to Australia, our scenarios predict rapid, northeast movement onto the Sahul Shelf into New Guinea due to the high carrying capacity predicted for the Gulf region.

Our model has also provided the capacity to test the relative likelihood of some alternative entry points, as well as the demographic context for the initial peopling of Sahul from Wallacea. First, the highest-ranked scenarios all point toward a prevailing entry via the southern route (via the Sahul Shelf). Any scenario where a northern entry occurred before a southern entry led to a lower relative ranking. This suggests that although the northern route was likely an easier pathway for entry^3^, the dominance of the southern route is more consistent with the available archaeological evidence (recognising that archaeological investigation of New Guinea remains limited to date). Given that our highest-ranking scenarios provided the strongest support for a southern entry, with a potential secondary northern entry, our treatment of temporally restricted (<700 years) initial peopling events of >1000 individuals^2^ is realistic. This also potentially implies that populations in Wallacea required sufficient time to grow large enough before widespread migration events took place^2^. Further, the mounting genetic evidence^13,23^ concurs with our conclusions regarding timing, support for a dual entry and Late Pleistocene population expansion. Unlike our model, however, genetics cannot yet shed light on precedence of particular or multiple entry points.

The highest-ranked relationship between carrying capacity and net primary production predicted a maximum continental-wide population of 6.3–6.5 million (0.55–0.57 people km^−2^) based on the carrying capacity predicted from the LOVECLIM Earth-systems model and the particular form of the best-supported relationship between human-population density and net primary production we considered. One of the main determinants of this range—which is substantially larger than previous reconstructions of population size for Late Pleistocene Sahul^49^—is the relatively higher support for the non-linear relationship (rotated parabolic) between human carrying capacity and net primary production compared to the other two forms. Although a non-linear relationship is consistent with evidence from elsewhere that ancient people selected habitats that were structurally intermediate (ecotones) between open plains and dense forests (e.g., savannas)^56^, thus providing vegetation mosaics that were more ecologically diverse and favourable to human visitation and settlement than maximum-productivity environments^57^, we did not have empirical data to support the exact form of that relationship. Testing the relative support among three different types of relationships does not mean that the rotated parabolic is in fact the correct one; instead, it merely gives more support for the hypothesis of non-linearity versus linearity. The combination of the spatial pattern of net primary production across Sahul (Supplementary Fig. 2) and the latter’s relationship with human density drives the magnitude of the total population estimate (Fig. 3). Without a specific, empirically supported relationship, it is entirely plausible that the total population size could have been substantially smaller, especially if other, unknown conditions prevented populations from achieving local carrying capacity. The true magnitude of the population estimate is therefore not central to our approach nor the ensuing results.

Nonetheless, the large estimate and the resulting population-density range are not at odds with post-contact estimates of hunter–gatherer densities across temperate coastal regions of Australia (mean ± SD: 0.34 ± 0.24 people km^−2^, with an upper limit of 0.81 people km^−2^ assuming a Gaussian distribution) and densities of shifting agriculturalists in New Guinea (4.37 ± 4.22 people km^−2^)^58^. In addition, the only existing estimates of population size in Sahul^49^ did not account for arrival before 50 ka, and instead focussed on periods much later than the arrival window we considered. Neither did those estimates include New Guinea, which represents ~15% of the Sahul landmass during the period of interest (and contains areas of higher ecological productivity than much of the remainder of Sahul), nor did they include the sunken coastal-shelf (~1.43 million km^2^, or ~19% more land area than Australia today). Overlaying the modern extent of Australia and New Guinea on the predicted population grid at 300 generations from initial entry indicates that ~27.5% of the continent’s total population is attributed to cells that are now under water (Fig. 3). In the case of the 50-ka single-entry scenario shown in Fig. 3, the above-water component of the Australian part of Sahul would give a total population of 3.1 million people at saturation.

The large assemblage of artefacts in the earliest phase of human occupation at Madjedbebe is also potentially consistent with a large population size^1^. For example, a 2 × 2-m area of the lower (Phase 2) assemblage at Madjedbebe contains tens of thousands of stone artefacts, >1000 pieces of ground ochre, >400 grindstones and ground fragments, >30 ground edge axes/fragments and includes distinctive technologies not recovered from the overlying layers (e.g., discoidal cores, thinning flakes, convergent flakes, points)^1^. This density of artefacts is greater than that seen for the Holocene layers at the site and the richness of cultural materials exceeds even that of many dense Holocene sites in Australia and New Guinea^5,59^. These data support the idea that Madjedbebe was intensively occupied, supportive of the early high population densities predicted by our model. In addition, recent evidence suggests that northern Sahul was relative wet during the period of human arrival as the intertropical convergence zone shifted south^60^, potentially increasing productivity in this region. Improving and expanding the archaeological record in places of initial arrival and where population densities were highest, including now-drowned but previously productive coastal-shelf sites^61^, must therefore form a research priority to improve model predictions of the development of human societies during the Late Pleistocene.

At a global scale, our conclusions have important implications for the migration of anatomically modern humans in the Late Pleistocene, and especially for those moving out of Africa. While specific to Sahul, our model does support the notion that during this period humans were able to migrate and occupy a wide range of environments and climatic zones, with only the most extreme arid environments forming barriers to movement in this region. Our models do not indicate a preference for coastal environments, with inland grasslands and rainforests settled first; these results lend support to the hypothesis of inland dispersal^62^. Our estimated migration rates of 0.71–0.92 km year^−1^ are consistent with previous estimates of rapid spread throughout Sahul from genetic evidence^12^. Our rates also provide a realistic model for maximum movement rates of humans across Eurasia, where as few as 12,000–15,500 years would be required to reach Sahul from Africa (assuming a minimum global-arc distance of 11,000 km; or 16,300–21,100 years assuming a minimum-distance coastal route of ~15,000 km). Assuming initial entry to Sahul between 75 and 50 ka, this lends support to arguments and genetic evidence^63^ for permanent movement out of Africa at the end of Marine Isotope Stage 5 or beginning of Stage 4, and perhaps movement into Sahul^64^ during both Stages 4 and 3.

Along with other recent models of the demographic conditions needed for successful entry to Sahul^2^, our model provides further evidence for the adaptability and technologically advanced state of anatomically modern humans during the Late Pleistocene. Our model also supports the notion of large populations being present across Southeast Asia and Sahul during this period^2,3^, which contrasts with past views of dispersed, small bands of hunter–gatherers. However, the lack of expansive archaeological records means that disparities persist, especially regarding the general absence of early sites in the vicinity of Bird’s Head and the highlands of New Guinea. There is a marked disparity also between the high maximum population sizes proposed here and the comparative paucity of documented archaeological sites across Sahul prior to the Last Glacial Maximum.

## Methods

### Cellular-automaton framework

We constructed the cellular-automaton model in the open-access R statistical computing environment (cran.rproject.org). We provide all code, data and instructions to repeat the analysis^65^, which can be run on any desktop computer. Our spatial model is based on a 0.5° × 0.5° raster grid of Sahul from 0.5 to 43.0° S latitude, and 110.5 to 153.5° E longitude (86 rows and 87 columns). The land area of Sahul changes with fluctuating sea levels, so we estimated exposed land in 1000-year time slices to follow our available hindcasts of carrying capacity (see ‘Carrying capacity’ below) based on a digital elevation model and estimated sea-level change over the period of interest (from 85 to 40 ka; see Scenarios). We used the ETOPO1 global relief model of Earth’s surface^66^ to estimate the exposed landmass of Sahul through time. To reconstruct the landmass changes of Sahul every 1000 years, we applied sea-level variability outputs^67^ to the ETOPO1 model. We also included fluctuations in Lake Carpentaria that could potentially act as a natural barrier for human movement over time. We modified the contour of the lake based on modelled sea-level changes^68^ applied to the digital elevation model.

From an initially peopled cell (see Scenarios), the new population can grow following a Ricker population-dynamics model, and emigrate to adjacent cells following stochastically resampled rules of dispersal; likewise, each cell can receive immigrants from adjacent cells following similar dispersal rules (see ‘Emigration and immigration’, and ‘Long-distance dispersal’).

### Population-dynamics model

Each cell within the grid acts as a particular sub-population unit within the overall dynamics of Sahul, and the summary information provided at the end of a simulation is an overall expression of all cells. The change in human abundance (*N*) within each cell is governed by the following phenomenological (Ricker) equation of population dynamics:

1 $$N_{i,j,t + 1} = N_{i,j,t}e^{r_m\left( {1 - \frac{{N_{i,j,t}}}{{K_{i,j,t}}}} \right)} - \left( {E_{i,j,t} - I_{i,j,t}} \right)$$

where *i* is the cell row number in the 0.5° × 0.5° latitude lattice, *j* is the cell column number, *t* is the time interval in units of human generations (1 *g* = 27.9 years)^2^, *N*_*i*,*j*,*t* + 1_ is the number of individuals in cell *i*, *j* at the next time interval (*t* + 1), *N*_*i*, *j*, *t*_ is the number of individuals in cell *i*, *j* at time interval *t*, *r*_*m*_ is the maximum rate of population increase when resources are not limiting, *K*_*i, j, t*_ is the cell-specific carrying capacity (see ‘Carrying capacity’ below), and the *E*_*i, j, t*_ and *I*_*i, j, t*_ parameters represent the number of individuals emigrating from and immigrating into the focal cell *i*, *j* per time interval *t*, respectively (see ‘Emigration and immigration’). As an estimate of *r*_*m*_, we set the age-structured Leslie matrix for Aboriginal hunter–gatherers^2^ to have a survival probability (subdiagonal matrix entries) all equal to 1 (complete survival in every age class), and then took the log_*e*_ of that matrix’s dominant eigenvalue to the power of *g* multiplied by 2 as the generationally scaled *r*_*m*_ estimate required for Eq. (1). Finally, we imposed a beta-resampled additional mortality parameter *M*_MVP_ = 0.2 for cells with a population size <100 individuals. This threshold of *N*_MVP_ = 100 is derived from the ecological concept of minimum viable population size^69^ where ~100 effective individuals are required to avoid inbreeding depression^70^. This assumes that individual fitness declines when populations decline below the minimum size due to inbreeding depression from non-random mating and other Allee effects related to social disruption.

### Carrying capacity

We constructed a theoretical carrying capacity (*K*) for each cell from a hindcasted estimate of net primary production based on the LOVECLIM climate reconstruction^2,3,35^. In this case, we define carrying capacity as the upper limit to total abundance^71^, which is expressed mathematically as the point at which net population growth becomes stable (*r* = 0)^72,73^. LOVECLIM is a three-dimensional Earth-system model of intermediate complexity^35^ that includes representations of the atmosphere, ocean and sea ice, land surface (including vegetation), ice sheets and the carbon cycle. LOVECLIM produces climates over the past 800 ka in 1000-year time-averaged increments (layers) that we downscaled (using a bilinear interpolation)^74,75^ from a spatial resolution of 5.625° × 5.625° to 0.5° × 0.5°. For each grid cell and each 1000-year layer, we extracted net primary production^76^ (kg C m^−2^ year^-1^) as the comprehensive indicator of relative carrying capacity through time^2^. To translate net primary production into a carrying capacity expressed in units of humans the landscape was capable of supporting, we used the predicted relationship between net primary production and human density for hunter–gatherer societies^37^. Here, we rescaled the net primary production values over all grid cells to concur with the minimum (0.018 km^−2^) and maximum (1.152 km^−2^) human densities, multiplied by cell area (3080.25 km^2^) to give per-cell *K*. We then linearly interpolated these values between the 1000-year layers for each cell.

This approach assumes a linear relationship between *K* and net primary production (Supplementary Fig. 2). However, others have suggested a non-linear relationship where maximum *K* occurs at mid-range net primary production, because past humans possibly struck a compromise between high productivity and ease of passage and/or visibility to hunt prey by tending towards ecotones of mid-range productivity^56,57^; this type of non-linear relationship has also been identified between herbivore biomass and mean annual rainfall across sub-Saharan Africa^77^. We therefore also applied a 180°-rotated parabola model between *K* and net primary production (*P*_*p*_) of the form:

2 $$K = a(P_p - h)^2 + K_{{\mathrm{max}}}$$

where we set *K*_max_ for the rotated parabolic relationship at 0.5*K*_max_ for the linear relationship, *a* = −3, *h* = the median of *P*_*p*_, and where we scaled the rotated parabolic Σ*K*_max_ across all cells and temporal layers to equal Σ*K*_max_ of the linear relationship (because of a higher frequency of cells with mid-range compared to high net primary production) (Supplementary Fig. 2). This approach assumes a logarithmic growth to and decline from a peak, so we also considered a third non-linear relationship—a reciprocal quadratic yield density—between *K* and *P*_*p*_ of the form:

3 $$K = \frac{{P_p}}{{a + bP + cP_p^2}}$$

where *a* = 200, *b* = 0.6 and *c* = 0.2, which instead assumes a logistic growth to and decline from a more pronounced peak (Supplementary Fig. 2). Whether we invoked the linear, rotated parabolic or reciprocal quadratic yield relationship (see Scenarios), we Poisson-resampled the resultant *K* for each cell per generational time step to simulate spatio-temporal uncertainty in *K*.

### Emigration and immigration

We reasoned that emigration out of a focal cell *i*, *j* to its eight immediately neighbouring cells would be a function of the gradient in *K*_*t*_ between two cells^44^, as well as how close the abundance *N*_*i*, *j*, *t*_ of cell *i*, *j* was to *K*_*i*, *j*, *t*_. If the Poisson-resampled *K*_*t*_ ratio between two adjacent cells (*K*_rel_ = *K*_*i*, *j*, *t*_/*K*_*i* + *y*, *j* + *x*, *t*_, where *x* and *y* are integers ranging from −1 to 1 to define the immediate one-cell neighbourhood of the focal cell *i*, *j*) was <1, and *N*_*i*, *j*, *t*_/*K*_*i*, *j*, *t*_ was > a random uniform proportion between 0.3 and 0.7, then emigration ensued. The values of 0.3 and 0.7 originate from Birdsell^78^ who stated that group ‘budding off’ or ‘fissioning’ occurred when a population reached 30–70% of carrying capacity. We further imposed an exponential decay function to describe the declining probability of emigration (Pr(*E*)) as the ratio of *K*_*i*, *j*, *t*_/*K*_*i* + *y*, *j* + *x*, *t*_ = *K*_rel_ increased towards 1 (Supplementary Fig. 6), where:

4 $$\Pr \left( E \right) = e^{ - 3.2K_{{\mathrm{rel}}}}$$

For the size of the emigrating group *N*_mig_, we assumed that this represented a beta-resampled proportion *P*_mig_ = 1/3, such that *N*_mig_ is centred on *N*_*i*, *j*, *t*_ × *P*_mig_ (with SD = 0.05*N*_*i*, *j*, *t*_ × *P*_mig_) based on the observation that when coastal populations of Aboriginal Australians fission, the population tends to separate into one larger and one smaller group, with the latter emigrating^79^. We applied this calculation for each of the eight cell comparisons, removing those already emigrated from each cell’s subsequent estimate (moving in sequence through directions NW, N, NE, W, E, SW, S and SE of focal cell *i*, *j*). Likewise, when *K*_*i*, *j*, *t*_/*K*_*i* + *y*, *j* + *x*,*t*_ > 1, immigration into cell *i*, *j* occurred following the same movement rules as for emigration.

### Long-distance dispersal

We used the allometric relationship of natal dispersal for omnivorous and herbivorous mammals^40^ to predict a dispersal probability for humans. Assuming a mean adult mass of *M* = 50 kg, maximum natal dispersal distance *D*_*m*_ is estimated as *aM*^*b*^, where *a* = 3.31 ± 1.17 and *b* = 0.65 ± 0.05 for omnivores and herbivores combined^40^. This produced an estimated maximum dispersal distance *D*_*m*_ ranging from 22.4 to 69.3 km. As a maximum dispersal range, this compares well to the average mobility of African hunter–gatherers of 1400–3900 km^2^/generation^39^ (equivalent to a radius of 21.1–35.2 km assuming a perfect circle), and the 0.4–1.1 km yr^−1^ (11.2–30.7 km/generation) estimates for Palaeolithic human expansions in northern Europe^41^. Also, Gould^38^ reported journeys by Aboriginal Australians of 400 to 560 km as ‘not unusual’ and perhaps the greatest mobility ever recorded, moving as many as nine times in three months, and covering an area of ~2600 km^2^ (radius = 28.8 km).

Next, we used the estimated probability of dispersal (Pr(*d*_max_)) of *d* exceeding multiples (1 to 10) of one cell width (0.5 × 111.12 = 55.6 km) as $\Pr \left( {d_{{\mathrm{max}}}} \right) = e^{ - d/aM^b}$ (Supplementary Fig. 7). However, there is evidence globally that the territory size of hunter–gatherer groups is strongly related to local productivity, with a greater need to expand foraging areas as productivity declines^19,80^. Using territory size and rainfall data from Hiscock^80^, we assumed the same relative change in rainfall applied to net primary productivity, but shifted the power–law relationship upwards to match the slope of the upper limit of maximum dispersal distance (Supplementary Fig. 7b). Thus, for every tenfold decrease in relative net primary production, maximum dispersal distance increases by 12.7 times (Supplementary Fig. 7b). Once a long-distance dispersal event occurred, we Poisson-resampled the maximum dispersal distance to provide a *δx* and a *δy* to move from the focal cell in cell units (including a random direction: east–west for *δx*, and north–south for *δy*). The size of the long-distance-dispersing population followed the same rules as for neighbouring-cell emigration.

### Distance-to-water limitation

While territory size, and hence, maximum dispersal distances increase with increasing aridity according to the relationships described above, there is evidence that human dispersal is ultimately limited by water availability^15^. This is likely to be even more relevant in Australia, the driest inhabited continent on Earth—indeed, estimated routes of gene flow among Aboriginal Australians suggest that the arid interior acted as a barrier to migration^11^. We therefore invoked an additional limitation on dispersal by calculating a probability of realizing a long-distance dispersal event (*P*_*l*_) according to the following equation previously designed to limit modelled species migrations^81^:

5 $$P_l = 1 - \left( {\frac{{D_l}}{{D_{H_2O}}}} \right)^{\Omega}$$

where *D*_*l*_ = the realized maximum dispersal distance generated from the algorithm described above, $D_{H_2O}$ = the distance to water in units of map cells derived from the Australian Water Observations from Space dataset^15^, and *Ω* = the hydrological resistance parameter set arbitrarily to a value of 3 to invoke landscape-scale resistance to movement only in the driest areas of Sahul per generational time step.

### Ruggedness

We hypothesized that high landscape ruggedness (elevational gradient) might at least partially impede the progress of human expansion across the landscape^42^, so we tested this using data available in an ethnographic and environmental dataset compiled by Binford^42^. Available in the binford library^82^ in R, the dataset includes >200 variables measuring aspects of hunter–gatherer subsistence, mobility and social organization for 339 ethnographically documented groups. Given the evidence that mobility is a function of productivity^36,80^, we constructed a simple linear model of annual movement varying with annual rainfall and the difference between maximum and minimum elevation within a 25- (40.2 km) mile radius of the group’s centroid (equivalent to an elevational gradient; i.e., ruggedness). Taking the cube root of annual movement and the difference in maximum and minimum elevation to comply with the assumption of Gaussian error distributions, the expected relationship between movement and rainfall prevailed, and there was a weak effect of elevational difference—a maximum of 1% reduction in annual movement (Supplementary Fig. 8). Expressed on the linear scale and standardizing annual movement and elevational difference to the range of 0–1 (assuming a constant median annual rainfall value), an exponential decay function of the form:

6 $$M_{{\mathrm{red}}} = a + b\root {3} \of {{G_{{\mathrm{rel}}}}}$$

where *M*_red_ = the proportion of expected total annual movement, *a* = 1.001116, *b* = −0.0104453 and *G*_rel_ = the standardized ruggedness from 0 to 1, described the reduction in annual movement rates up to a maximum of 1% (Supplementary Fig. 8). For all instances of emigration, immigration and long-distance dispersal, we assigned this function to the total number of people migrating for each cell based on its standardized ruggedness. We computed the topographic ruggedness index^83^ as the difference in elevation between a given cell and its eight neighbouring central cells, based on our digital elevation model. For a given cell, we then squared each of the eight elevation difference values (to render them positive), and calculated the square root of the averages of the squares. We updated the spatial resolution of our results to 0.5° × 0.5° to match the other environmental layers.

### Catastrophic mortality events

Palaeo-demographic investigations of past human populations suggest that long-term population growth rates were just slightly higher than zero as a result of episodes of catastrophic mortality arising from pandemics, natural disasters and violent conflicts occurring every few generations^84^. This also agrees well with estimates of the probability of mass mortality events scaling to generation time for vertebrates (Pr(catastrophe) = 0.14 per generation)^43^. We thus sampled binomially at Pr = 0.14 for whether a catastrophe occurred in each focal cell, and then beta-sampled the severity of the event centred on *M*_cat_ = 0.5 (SD = 0.5/10) to emulate a stochastic catastrophe event of 50% mortality, on average, for that cell^43^.

However, we reasoned that a random allocation of catastrophes among cells across the entirety of Sahul was not realistic, for the reason that mortality events arising from natural disasters, warfare or disease outbreaks would likely be spatially aggregated. We therefore imposed a Thomas cluster process using the *rThomas* function from the spatstat R library^85^, setting the intensity of the Poisson process of cluster centres *κ* to a linear relationship between the number of cells occupied per iteration and a vector ranging from 0.3 to 1.2, the standard deviation of random displacement along each coordinate axis of the grid of a given cell away from the cluster centre *σ*_scale_ = 0.015, and the mean number of cells per cluster *μ* = 0.6 × the mean dimension of the occupied grid per iteration. This combination of parameters led to a reasonable degree of spatial clustering while maintaining a random spread of cells around a catastrophe focal point, as well as maintaining the overall proportion of cells across the landscape experiencing a catastrophic mortality event ~0.14 per generational iteration.

### Scenarios

We ran 120 scenarios (8 entry times, ×5 entry sequences, ×3 relationships between carrying capacity and net primary production) where we modified three main components of the stochastic simulations: (i) the timing of first entry to Australia (from 85 to 50 ka, in 5000-year increments), (ii) the place of entry (northern, southern, simultaneous northern and southern, northern followed by southern 2000 years later, or southern followed by northern 2000 years later) and (iii) the form of the relationship between hindcasted net primary productivity and human carrying capacity (linear, rotated parabolic or reciprocal quadratic yield density). We repeated each scenario 100 times to generate a per-cell confidence interval of time of first arrival. Here, we deemed a cell to have been populated for the first time once it received ≥100 individuals (*N*_first_), which is considered the minimum viable effective population size to avoid inbreeding depression^70^.

### Comparison layers

To test the resultant outputs against real archaeological data, we compiled a conservative list of ages older than 30 ka obtained from across Sahul (see ‘Compiling reference archaeological dates’ in the Supplementary Information and Supplementary Data 1). However, the spatial coverage of these ages is highly uneven (Fig. 1a), so we applied a maximum-likelihood method to correct for the Signor–Lipps effect first developed by Solow^86^ and adapted for spatial inference of both first-arrival and extinction patterns^87^. While described in more detailed elsewhere^87^, we briefly summarize the approach here.

To correct for the inherent spatial bias of dates in a landscape, let *x*_1_,…*x*_*n*_ be the spatial locations of *n* dated specimens in an area *W* and *a*_1_,…*a*_*n*_ their respective ages. The estimated average age *M*(*x*) of a putative date at a given location *x* is based on a standard kriging procedure^88^ derived from the spatial covariance between the age of two dated specimens as a function of their respective pairwise distance, so that:

7 $$\hat M\left( x \right) = \mathop {\sum}\limits_{i \le n} {w_i\left( x \right)a_i}$$

where $w_1\left( x \right), \ldots w_n\left( x \right)$ follows $\mathop {\sum}\nolimits_{i \le n} {w_i\left( x \right) = 1}$ and minimizes

8 $$\mathop {\sum}\limits_{i \le n} {w_i\left( x \right)\gamma \left( {x_i - x_j} \right) + \mu = \gamma (x - x_j)}$$

for *j* ≤ *n*, with *μ* being a Lagrange multiplier so that $\mu = \mathop {\sum}\nolimits_{i \le n} {\gamma (x_i - x)}$ and *γ* is the variogram:

9 $$\gamma \left( u \right) = \frac{1}{2}E\left( {a\left( z \right) - a\left( {z + u} \right)} \right)^2 = \sigma ^2 - c\left( u \right)$$

where *a*(*z*) is the age *a* of a specimen found at a given location *z* (with *z* ∈ *W*), *σ*^2^ is the variance of *a*(*z*) and *c*(*u*) is the covariance between *a*(*z*) and *a*(*z* + *u*), with any two locations in *W* separated by distance *u*.

We then modified Solow’s method^89^ to correct for taphonomic bias, which assumes initially that the distribution of ages through time is uniform between a given age *A*_0_ when individuals are assumed to be present, and the date of arrival *A*. For *n* ages of a given time series at a given location, the estimated terminal age $\hat A$ is therefore:

10 $$\hat A = A_0 + \frac{{n + 1}}{n}{\max} _{i}\left( {a_i - A_o} \right)$$

To integrate this method into a spatial context, we estimated a preliminary age *A*_*p*_ across space assuming $\hat M\left( x \right)$ follows a stationary random field:

11 $$\hat A_p\left( x \right) = 2\hat M\left( x \right) - A_0$$

But this generates a spatial bias $\hat A_p\left( x \right) - A(x)$, in every $\hat A_p\left( x \right)$, so we applied a simulation-based, spatial-bias-correction procedure^90^ to estimate the bias generated by Eq. (11) at each *x* across *W*. The first step assumes that $\hat A_p\left( x \right)$ is the ‘true’ date of the terminal event in *x*. Based on these $\hat A_p\left( x \right)$, we generated *k* age samples $a^{(k)} = (a_1^{\left( k \right)}, \ldots ,a_n^{(k)})$ at the same locations *x*_1_, … *x*_*n*_ following the same spatial pattern and characteristics as the dated record and sampled independently from a uniform distribution on $[A_{0,}\hat A_p\left( {x_i} \right)]$. We then inferred $\hat A^{(k)}(x)$, the timing of the terminal event for the *k* new simulated time series and calculated an estimated total bias $\hat B\left( x \right)$ across all *k* ages:

12 $$\hat B\left( x \right) = \frac{1}{k}\mathop {\sum}\limits_k {\hat A^{\left( k \right)}\left( x \right) - \hat A_p\left( x \right)}$$

The final estimate of the timing of the terminal event of interest $\hat A\left( x \right)$ is the distribution of the preliminary dates $\hat A_p\left( x \right)$ for every location *x* corrected by $\hat B\left( x \right)$, such that:

13 $$\hat A\left( x \right) = \hat A_p\left( x \right) - \hat B\left( x \right)$$

Because archaeological age estimates *a*_*i*_ are always associated with an inherent dating uncertainty *σ*_*i*_, we assumed that age uncertainties are Gaussian and independent^91^ so that the probability density of the estimated age of the terminal event *A*(*x*) follows:

14 $$\int_{{\it{\epsilon }}_1,...,{\it{\epsilon }}_n} {A_{\left\{ {a_1 + {\it{\epsilon }}_1, \ldots \;a_n + {\it{\epsilon }}_n} \right\}}(x)\prod _ig_i\left( {{\it{\epsilon }}_i} \right)d{\it{\epsilon }}_1 \ldots d{\it{\epsilon }}_n}$$

where g_*i*_ = the density of the Gaussian random variable with mean 0 and variance $\sigma _i^2$, and $A_{\left\{ {a_1 + {\it{\epsilon }}_1,\, ..\, a_n + {\it{\epsilon }}_n} \right\}}(x)$ = the final estimate at a given location *x* for a time series of age $a_1 + {\it{\epsilon }}_1,\, ..\, a_n + {\it{\epsilon }}_n$ located at *x*_1_,..*x*_*n*_, respectively. We applied the same Cook and Stefanski bias-correction procedure so that the *k* ages are independently sampled from a uniform distribution on $[A_{0,}\hat A_p\left( {x_i} \right)]$ at the same locations *x*_1_, … *x*_*n*_ following the same spatial pattern and characteristics as the dated record. This gives $a^{(k)} = (a_1 + {\it{\epsilon }}_1^{(k)},\, ..\, a_n + {\it{\epsilon }}_n^{(k)})$ with ${\it{\epsilon }}_i^{(k)}$ independently sampled as a function of the probability density described in Eq. (14) to account for the dating uncertainty associated with each age. We then use the terminal ages $\hat A^{(k)}(x) = A_{\left\{ {a_1^{\left( k \right)},\,..,\, a_n^{\left( k \right)}} \right\}}\left( x \right)$ to estimate the bias in Eq. (12) and apply this to provide a corrected timing of $\hat A\left( x \right)$ for every *x* following Eq. (13).

### Global sensitivity analysis

We designed a global sensitivity analysis to provide robust sensitivity measures of the probability of the time to saturation of the entire Sahul continent to variation in the underlying parameters of our stochastic model^54,92^; this analysis does not repeat the scenario-testing parameters (i.e., time of entry, point(s) of entry, *K*–*P*_*p*_ relationship). For this global sensitivity analysis, we used the initial scenario parameters of a 50-ka entry at the southern route, the rotated parabolic relationship between *K* and *P*_*p*_, and assuming a founding population size stochastically sampled between 1300 and 1500 people for the entry point^2^.

Here, we ran the cellular-automaton spatial model 1000 times, randomly sampling 12 of its parameters uniformly for each iteration based on a Latin hypercube-sampling protocol^54^. We set the 12 parameters to be sampled with ±50% variation on the median value used in the model (except for *N*_cat_ and max *N*/*K* with a maximum upper bound of 0.99, and for maximum *D*_cell_—see below); these 12 parameters were: (i) the maximum generational rate of population increase *r*_*m*_ used to parameterize the phenomenological population-dynamics model per cell (range: 0.10–0.31), (ii) the minimum maximal dispersal distances *D*_*m*_ estimated from the allometric prediction (11–34 km), (iii) the cell-based maximum dispersal distance modifier (max *D*_cell_), ranging from 1× to 5× the value set in the original model (10 cells), (iv) the cell-based minimum viable population size *N*_MVP_ (50–150 individuals) below which we set (v) an additional mortality parameter *M*_MVP_ (0.1–0.3), (vi) the hydrological resistance parameter *Ω* invoking landscape-scale resistance to movement only in the driest areas of Sahul per generational time step (1.5–4.5), (vii) the beta-resampled mean mortality of a cell during a catastrophe event *M*_cat_ (0.38–0.99), (viii) the beta-resampled proportion of people moving between cells when a migration event occurs *P*_mig_ (0.17–0.50), the beta-resampled (ix) minimum and (x) maximum ratios of *N*/*K* per cell invoking an emigration event (0.15–0.45 and 0.35–0.99, respectively), (xi) a resistance modifier *R* that modified the relationship between landscape ruggedness and maximum dispersal probability (0.5–1.5) and (xii) the population threshold *N*_first_ above which we determined a cell to be occupied for the calculation of the date of first arrival in a cell (50–150 individuals).

We chose to summarise the output of each of these 1000 parameter-sampled runs of the spatial model as the time taken to achieve continental saturation (i.e., the number of years taken from initial entry to occupy every cell in Sahul). In a separate analysis, we then tested the influence of the per-model run parameter values (predictors) on the time to continental saturation (response) using a boosted-regression tree^93^ emulator with the function gbm.step in the dismo R library^94^. Here, we set the error distribution family as Gaussian, the bag fraction to 0.75, the learning rate to 0.008, the tolerance to 0.0001, the maximum number of trees to 10,000 and the tree complexity to 2 (first-order interactions only). To assess the relative contribution of each of the 12 randomly sampled parameters to the time to spatial saturation, we calculated the boosted-regression tree metrics of relative influence^54^.

### Reporting summary

Further information on research design is available in the Nature Research Reporting Summary linked to this article.

## Supplementary information

Supplementary information

Peer Review File

Reporting Summary

Description of Additional Supplementary Files

Supplementary Data 1

## Data Availability

All data are available for download at github.com/cjabradshaw/SahulHumanSpread (10.5281/zenodo.4453767).

## Code availability

All R code is available for download at github.com/cjabradshaw/SahulHumanSpread (10.5281/zenodo.4453767).
