# Supplementary material for: Stochastic models support rapid peopling of Late Pleistocene Sahul
Source: Nat Commun. 2021 Apr 29;12:2440. doi: 10.1038/s41467-021-21551-3 (PMC8085232; doi:10.1038/s41467-021-21551-3)
Supplement: Supplementary file 1 — Supplementary information [file 41467_2021_21551_MOESM1_ESM.docx]

Supplementary Methods

**Stochastic models support rapid peopling of Late Pleistocene Sahul**

Corey J. A. Bradshaw, Kasih Norman, Sean Ulm, Alan N. Williams, Chris Clarkson, Joël Chadoeuf, Sam C. Lin, Zenobia Jacobs, Richard G. Roberts, Michael I. Bird, Laura S. Weyrich, Simon G. Haberle, Sue O’Connor, Bastien Llamas, Tim J. Cohen, Tobias Friedrich, Peter Veth, Matthew Leavesley, Frédérik Saltré

***Compiling reference archaeological dates***

We constructed the reference archaeological age dataset for Sahul by building on the *AustArch* compilation^1^ (see Supplementary Data 1). We selected 30,000 BP (years before present) for radiocarbon ages and 30,000 years ago for other techniques as an arbitrary threshold to encompass ages representing the earliest phases of human occupation of the continent. We removed ages younger than 30,000 BP/30,000 years ago and those documented as not directly related to human occupation (for example, ages taken on samples below archaeological deposits) from the *AustArch* dataset. We systematically compiled ages published since the completion of *AustArch* in 2014 from the literature. These included several sites in the north and northwest of Australia, with suites of new ages from Madjedbebe^2^, Nawarla Gabarnmang^3^, Carpenter’s Gap 1^4,5^, Riwi^6^, and Kimberley regions (Minjiwarra)^7^ that we added to the dataset. We also included recent research in Australia’s central-west coast (Boodie Cave)^8^, Western Desert (Karnatukul)^9^, and Pilbara^10-15^. We then added new dates from excavations at Warratyi^16^ and Moyjil^17^, and a few ages of > 30 ka omitted in the original *AustArch* compilation (including hearth ‘PACD H1’)^18^ and Kakutungutanta^19^.

The *AustArch* dataset is confined to Australia, so to extend the reference archaeological ages to encompass all of Sahul, we consulted syntheses of early ages from New Guinea^20^ to identify original sources^21-31^, which we then reviewed in detail. We did not include archaeological sites on islands that have never formed part of the Sahul landmass (e.g., the Bismarck and Solomon Archipelagos) in the dataset.

We consulted the original sources for all > 30 ka (1 ka = 1000 years before present) ages to compile details about the dating technique, with a particular focus on sample pretreatment, provenance and cultural associations. Missing sample pretreatment information was, where available, provided by the University of Waikato Radiocarbon Dating Laboratory and the Australian National University Radiocarbon Laboratory, or in some cases we obtained this information directly from the authors.

We quality rated ages largely following the schema developed by Rodríguez-Rey and colleagues^32^ for evaluating ages associated with Sahul megafauna deposits. Quality rating was a two-step process resulting in allocating ages to one of four categories of reliability (ranging from *A**, *A*, *B* to *C*, with *A* =* highly reliable and *C =* not reliable)^32^. The first step evaluated the dating technique itself resulting in assignment of the age to one of four categories (ranging from *m**, *m*, *B* to *C*, with *m** = reliable dating technique and *C =* unreliable dating technique)^32^. The second step evaluates the strength of association of ages rated *m** and *m* with the dating target, in this case the association of the sample with cultural deposits, resulting in a final reliability rating (*A**, *A*, *B* and *C*). Where a sample is reported as directly associated with a stratigraphic unit containing cultural deposits, we have accepted it as ‘associated’.

We deviated from Rodríguez-Rey and colleagues’ approach^32^ in accepting charcoal ages pretreated with acid-base oxidation (ABOx) as ‘*m**’ (rather than ‘*m*’) and acid-base-acid (ABA) or acid-alkali-acid (AAA) as ‘*m*’ (rather than ‘*B*’). We also rated hydrogen pyrolysis (hypy) as ‘*m*’, which was not included in Rodríguez-Rey et al.^32^. For marine shells, we rated samples subject to either x-ray diffraction or Feigl staining as ‘*m**’ rather than ‘*m*’. The original schema only accepted charcoal ages pretreated with ABOx. Although ABOx remains the gold standard for pretreatment of older charcoal samples (> 30 ka)^33^, only a few samples in Sahul have been subjected to ABOx pretreatment, and in some cases only negligible differences are observed between ABA- and ABOx-pretreated samples^3,34^, whereas in other cases differences can be non-negligible^35^. Including all ABA- and AAA-pretreated samples therefore permits a much broader consideration of the available data, but also carries with it some potential for age underestimation. Quality ratings for other dating techniques follow the criteria outlined in detail in Rodríguez-Rey et al.^32^.

We reduced the dating quality of any infinite age or any age without a reported error to *C*. We reduced the overall quality rating of any age > 75 ka to a *C*. We also reduced the overall quality rating of any age to a *C* where the authors reported the age as unreliable or not associated with cultural material. We calibrated all radiocarbon dates to calendar dates before present using the Southern Hemisphere Calibration curve (ShCal13)^36^ from the OxCal radiocarbon calibration tool version 4.2 (c14.arch.ox.ac.uk)^37^.

For radiocarbon ages with asymmetrical errors, we used the larger of the errors in all analyses. Clearly the choice of date inclusion/exclusion will have bearing on the ranking of the tested scenarios. To that end, we derived a second archaeological layer where we excluded all of the dates^2,38-44^ from the Madjedbebe site, and then redid the Signor-Lipps- and spatial bias-corrected layer for comparison (Fig. 1c). Rerunning all scenarios as for the original archaeological layer, we generated a new scenario rank (Supplementary Fig. 3 and 4).

In addition to the limitations of dating procedures and the uncertainties associated with the available ages, we recognize that the oldest ages for human occupation might be underestimated for some sites, because (*i*) we removed infinite radiocarbon ages from the dataset, (*ii*) earlier archaeological layers were not dated (e.g., Nawarla Gabarnmang)^3^, and/or (*iii*) potentially older occupation deposits are no longer preserved (e.g., sites such as Carpenter’s Gap 1, where the lowest archaeological layer sits directly on bedrock)^4,5^.

***Spatial correspondence between modelled and archaeologically derived dates of initial arrival***

For each scenario, we tested the correlation between the modelled date of initial arrival for each cell in the landscape and the date derived from the bias-corrected archaeological layer. To do this, we randomly resampled cells from the landscape for both the estimated arrival times from the modelled scenarios and the validation layer, and determined the Spearman’s *ρ* for these cells. This approach partially avoids spatial autocorrelation by reducing the number of adjacent cells in the sample. We then calculated the upper and lower 95% confidence limits for *ρ* for each of the 100 iterations of the validation run for each scenario tested. We then resampled those *ρ* across all iterations to provide the correlation coefficients’ confidence intervals reported in Supplementary Fig. 1 and 4. Where the confidence intervals do not overlap 0, the values are statistically non-random.

It is important to understand that the actual value of *ρ* is not the central metric of the congruency analysis. What is instead important is the relative change in *ρ* among the scenarios tested. The fully stochastic model built from real data combined into functions that capture human behavioural phenomena as they respond to temporal and spatial variability in environmental carrying capacity cannot be expected to return high correlation coefficients as might be expected from a simple bivariate analysis. Comparing the emergent property of these many different processes (peopling of cells progressively over time) to an equally uncertain expression of actual human presence derived from the archaeological data (corrected for spatial and preservation biases) necessarily implies lower expected correlation, especially across nearly 4000 cells. Therefore, the comparison is not a validation as such, but instead a congruency. A validation requires a set of known (observed) attributes used to ‘validate’ the predictions of a model. In the current case, however, the archaeological data are not measured observations of a phenomenon — they are themselves subject to extensive uncertainty, including but not restricted to dating uncertainty, contamination, preservation bias, and sampling bias. They cannot therefore be used to validate the model predictions. Compiling these archaeological dates into a single layer incorporating both their inherent biases and uncertainties, in addition to the real (and often ignored^45^) spatial biases they create, means that the resultant archaeological date layer is itself a modelled output. We can, however, examine congruency between that layer and the model predictions to demonstrate which of the scenarios tested is (more) ecologically realistic.

**Supplementary Fig. 1** Spearman’s rank (*ρ*) correlations for 40 simulation scenarios compared to the primary archaeological layer. Initial scenario parameters include date of first entry (50k, 55k, 60k, 65k, 70k, 75k, 80k, or 85k), and place(s) of entry (S = southern route; N = northern route), as well as any time lags between entry points (S-N = simultaneous entry at both routes; S-72N = southern entry initially followed by one through the northern route 72 generations later; N-72S = northern entry initially followed by one through the southern route 72 generations later). All scenarios are based on the rotated parabolic relationship between human carrying capacity and net primary production (see Supplementary Fig. 2). Error bars represent 95% confidence intervals of 100 resampled iterations of each scenario.

**
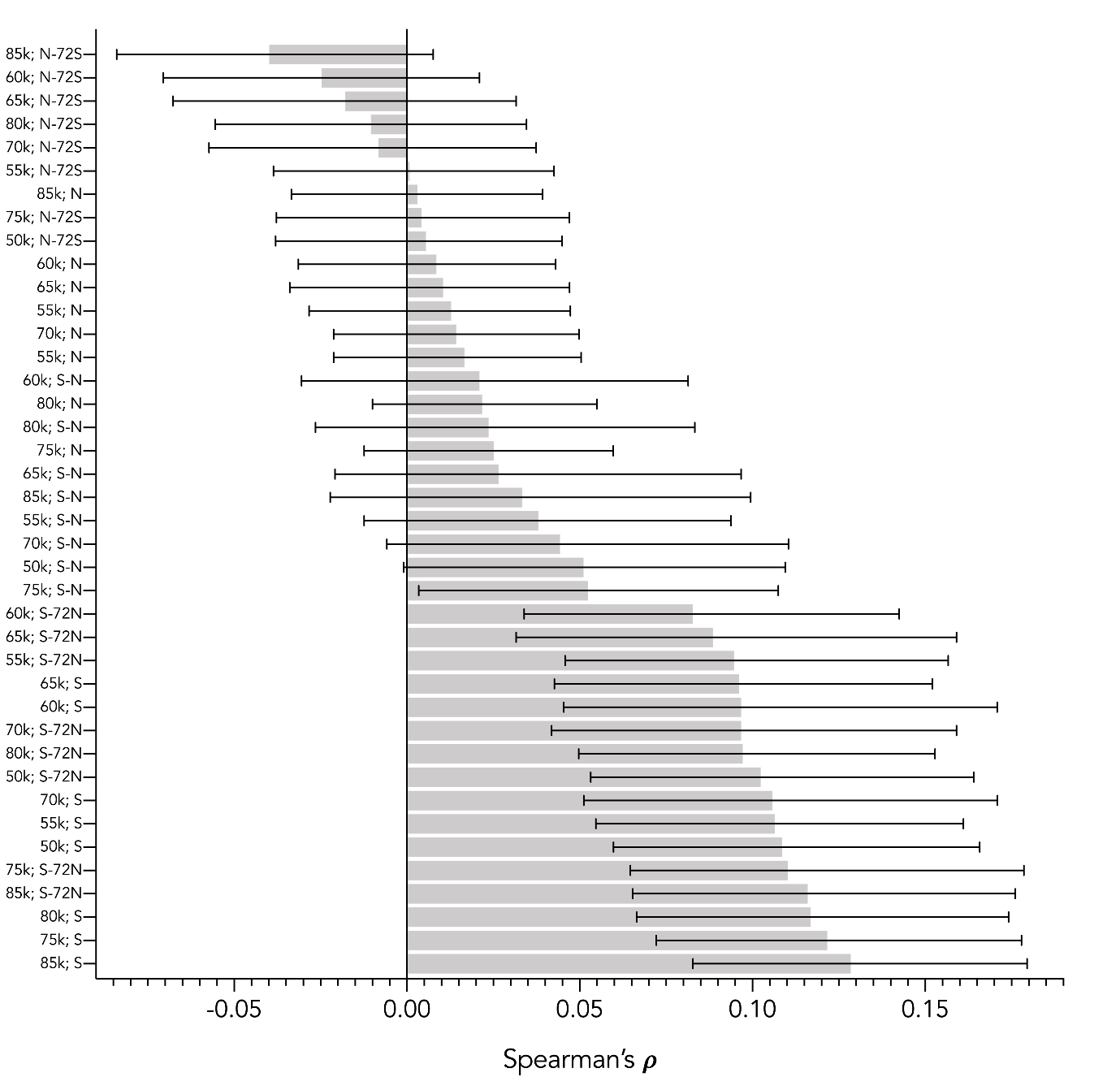
**

**Supplementary Fig. 2** (a) Assumed relationships between LOVECLIM-hindcasted net primary production and human carrying capacity (*K*, individuals per cell): linear, rotated parabolic, and reciprocal quadratic yield density. The probability density distribution (frequency histogram standardised so the values sum to 1) of cell-specific carrying capacities for the (b) linear, (c) rotated parabolic, and (d) reciprocal quadratic yield density across the entire Sahul landscape are shown, as well as the map distributions of *K* at 50 ka according to the (e) linear, (f) rotated parabolic, and (g) reciprocal quadratic yield density relationships. See Methods (Cellular-automaton framework) for the source of the map extent for Sahul.


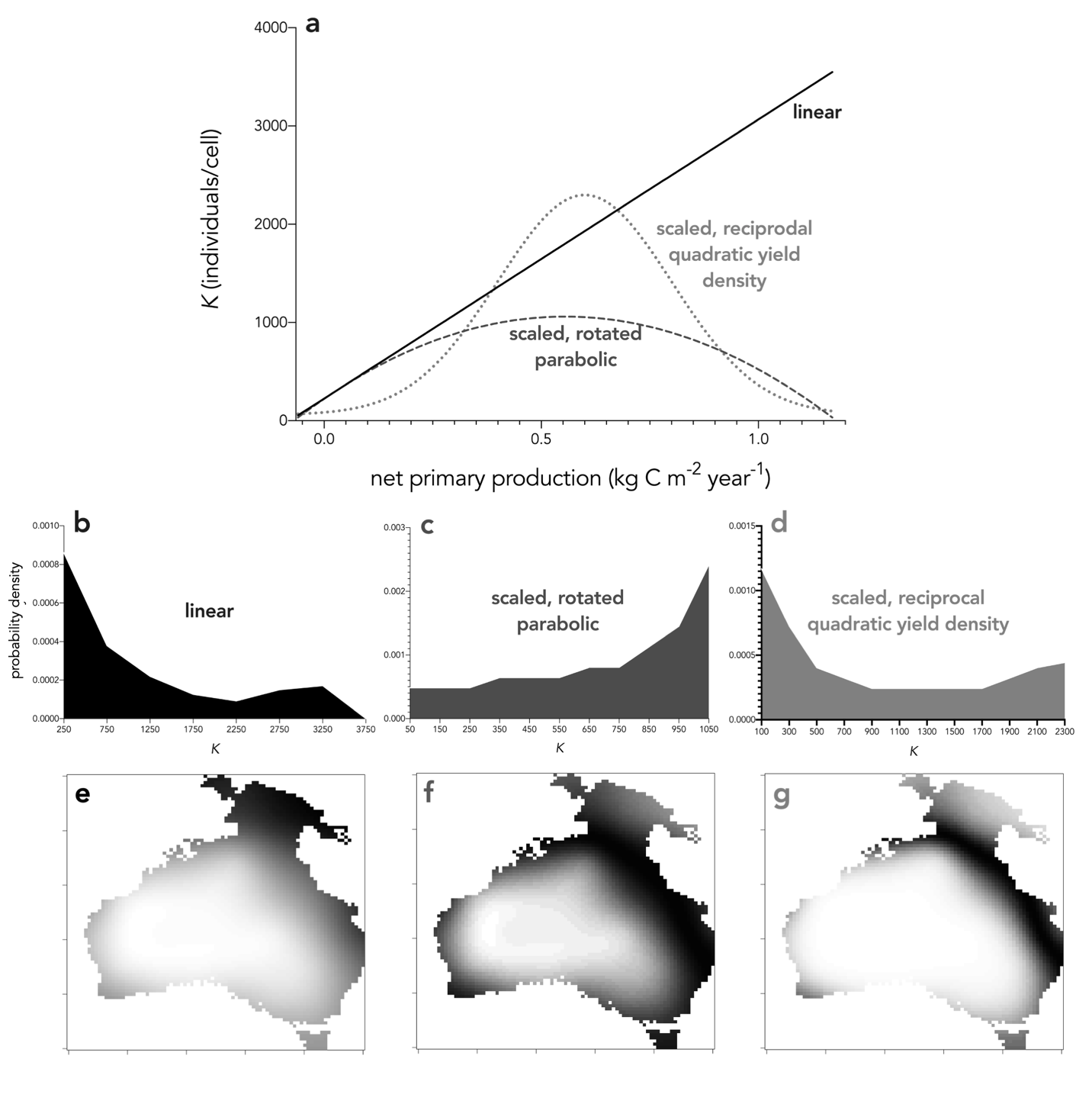


**Supplementary Fig. 3** Relative reduction in Spearman’s *ρ* rank correlation between the predicted time of first arrival over all grid cells for each scenario and the Signor-Lipps- and spatial bias-corrected archaeological layer without the Madjedbebe dates. Holding all other input parameters equal, the mean relative change in average is *ρ* expressed as a function of modifying (a) arrival time (from 85 to 50 ka, in 5 k increments; 5 scenarios for each increment), (b) entry point sequence (S = southern route through the Sahul Shelf; N = northern route through Bird’s Head of New Guinea, and combinations of these with lags expressed in terms of human generations: 72 generations ~ 2000 years; 8 scenarios each), and (c) assumed relationship between human carrying capacity (*K*) and hindcasted net primary production (*P_p_*) (qyd = reciprocal quadratic yield density; lin = linear; para = rotated parabolic — see *Methods* for details; 3 scenarios each). The horizontal bars represent the mean relative change in *ρ* and the error bars represent standard errors of the mean.

**Supplementary Fig. 4** (a) Spearman’s rank (*ρ*) correlations for 40 simulation scenarios compared to the archaeological layer without the Madjedbebe dates (cf. Supplementary Fig. 1). Initial scenario parameters include date of first entry (50k, 55k, 60k, 65k, 70k, 75k, 80k, or 85k), and place(s) of entry (S = southern route; N = northern route), as well as any time lags between entry points (S-N = simultaneous entry at both routes; S-72N = southern entry initially followed by one through the northern route 72 generations later; N-72S = northern entry initially followed by one through the southern route 72 generations later). All scenarios are based on the rotated parabolic relationship between human carrying capacity and net primary production (see Supplementary Fig. 2). Error bars represent 95% confidence intervals of 100 resampled iterations of each scenario.

**
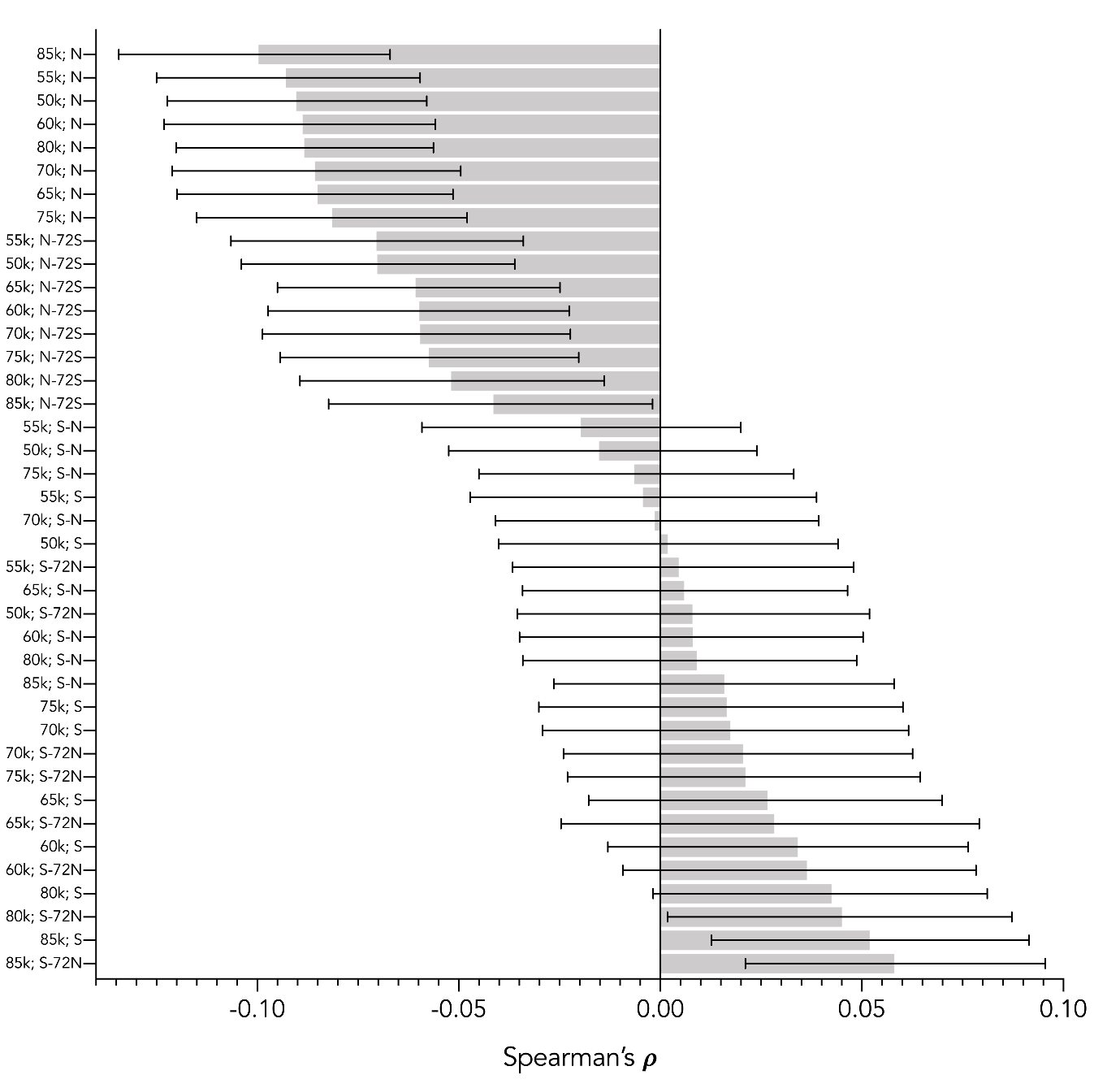
**

**Supplementary Fig. 5** Fitted relationships between variation in the five most-influential model parameters on the predicted time to continental saturation (see also Fig. 5). (a) A 50% reduction in the lower limit of the proportion of people migrating out of a cell (*P*_mig_) produced a 21% slowing of the time to saturation (a 50% increase in *P*_mig_ produced a 11% increase in the time to saturation). (b) A 50% reduction in mean catastrophe mortality (*M*_cat_) produced a 17% increase in the time to saturation (a 14% increase in *M*_cat_ produced a 3% faster time to saturation). (c) Multiplying the maximum cell-based dispersal distance by five times produced a 5% faster time to saturation. (d) A 50% reduction in the cell minimum viable population size (*N*_MVP_) produced a 4% faster saturation time (whereas a 50% increase in *N*_MVP_ slowed saturation time by 2%). (e) A 50% reduction in the additional mortality below MVP (*M*_MVP_) produced a 3% faster saturation time (whereas a 50% increase in *M*_MVP_ slowed saturation time by 4%. Settings for the boosting regression tree were: error distribution = Gaussian, bag fraction = 0.75, learning rate = 0.008, tolerance = 0.0001, maximum number of trees = 10,000, and tree complexity = 2.

**
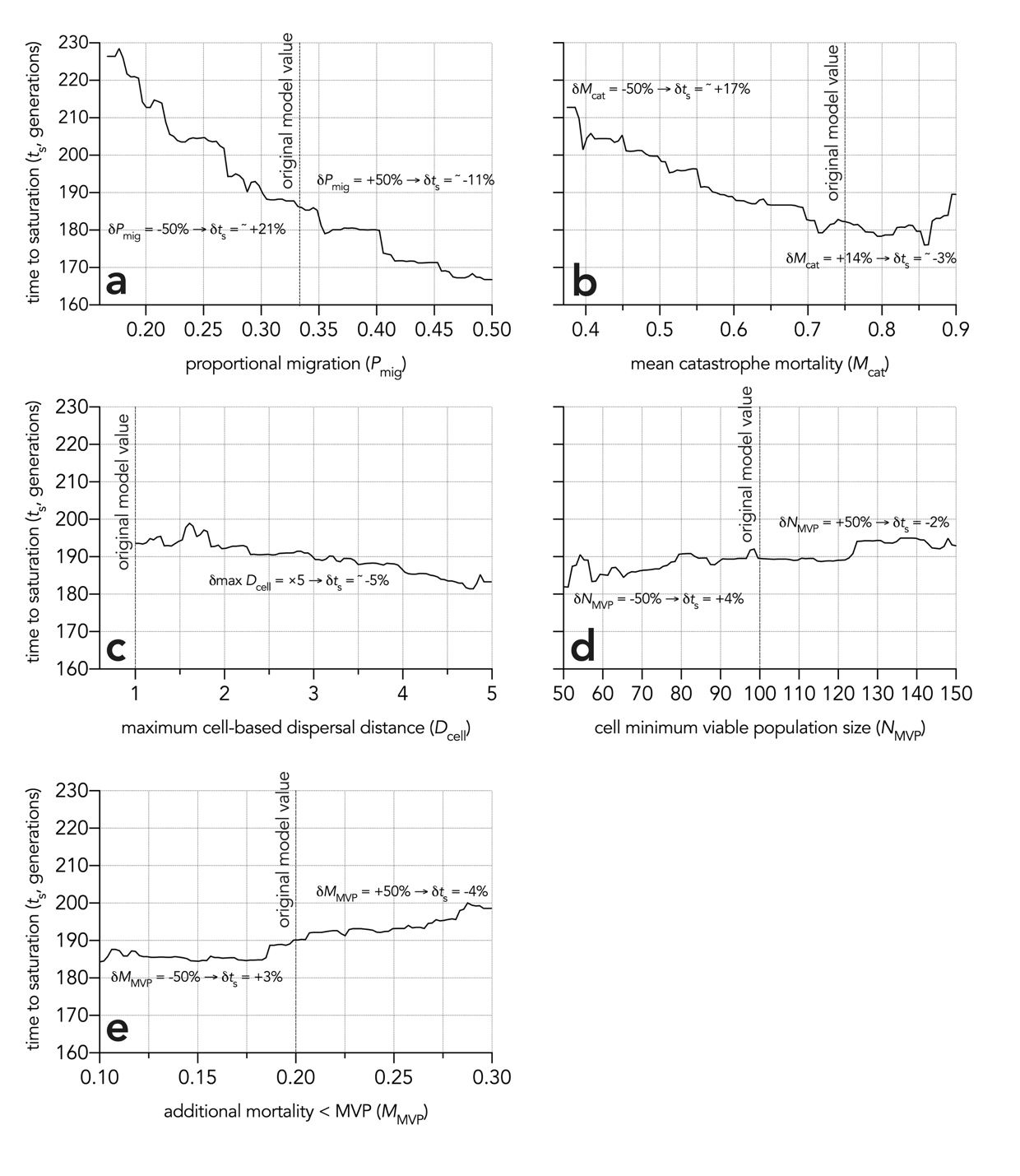
**

**Supplementary Fig. 6** The exponential decay function ($\Pr\left( E \right)=e^{-3.2K_{\text{rel}}}$) describing the declining probability of emigrating (Pr(*E*)) from a focal cell *i*, *j* at time *t* as the ratio of cell carrying capacities *K_i_*_,_*_j_*_,_*_t_*/*K_i_*_+_*_y_*_,_*_j_*_+_*_x_*_,_*_t_* (*K*_rel_) to the target cell *i*+*y*, *j*+*x* increases toward 1.


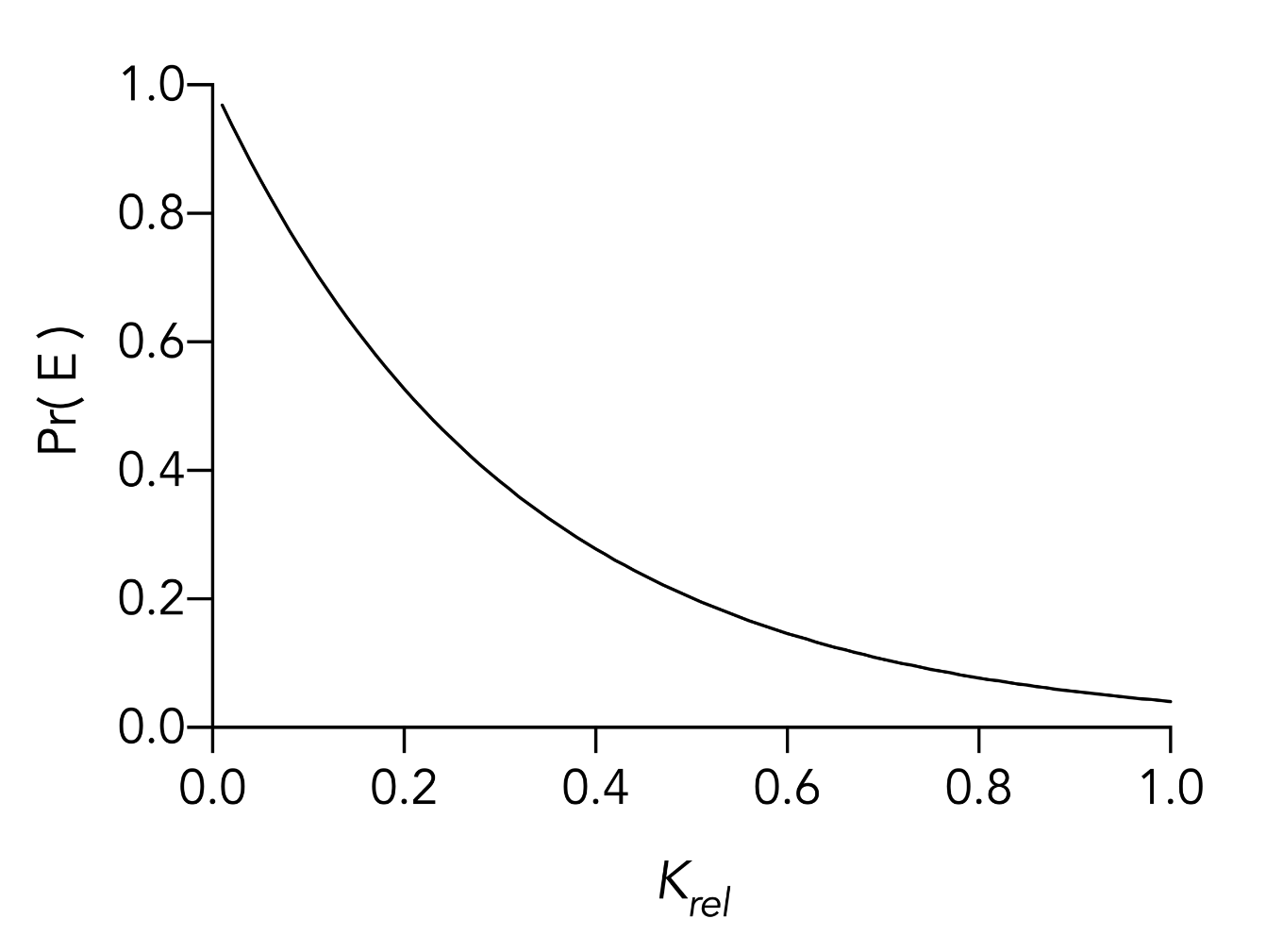


**Supplementary Fig. 7** (a) Estimated probability of dispersal, Pr(*D*_m_), of distance *D* exceeding multiples (1 to 10) of one cell width (0.5×111.12 = 55.6 km) according to the equation: $\Pr\left( D_{\text{m}} \right)=e^{{-d}/{aM^{b}}}$. The shaded area represents the uncertainty in the allometric prediction of maximum dispersal distance of 22.4 to 69.3 km expressed in in terms of number of grid cells across the probability range. (b) Re-configured relationship between territory size and hindcasted net primary production assuming the same relationship between territory size and rainfall from Hiscock^46^. We shifted this power-law relationship upwards to match the slope of the upper limit of maximum dispersal distance, meaning that for every 10-fold decrease in relative net primary production, average maximum dispersal distance declined by 12.7 times.


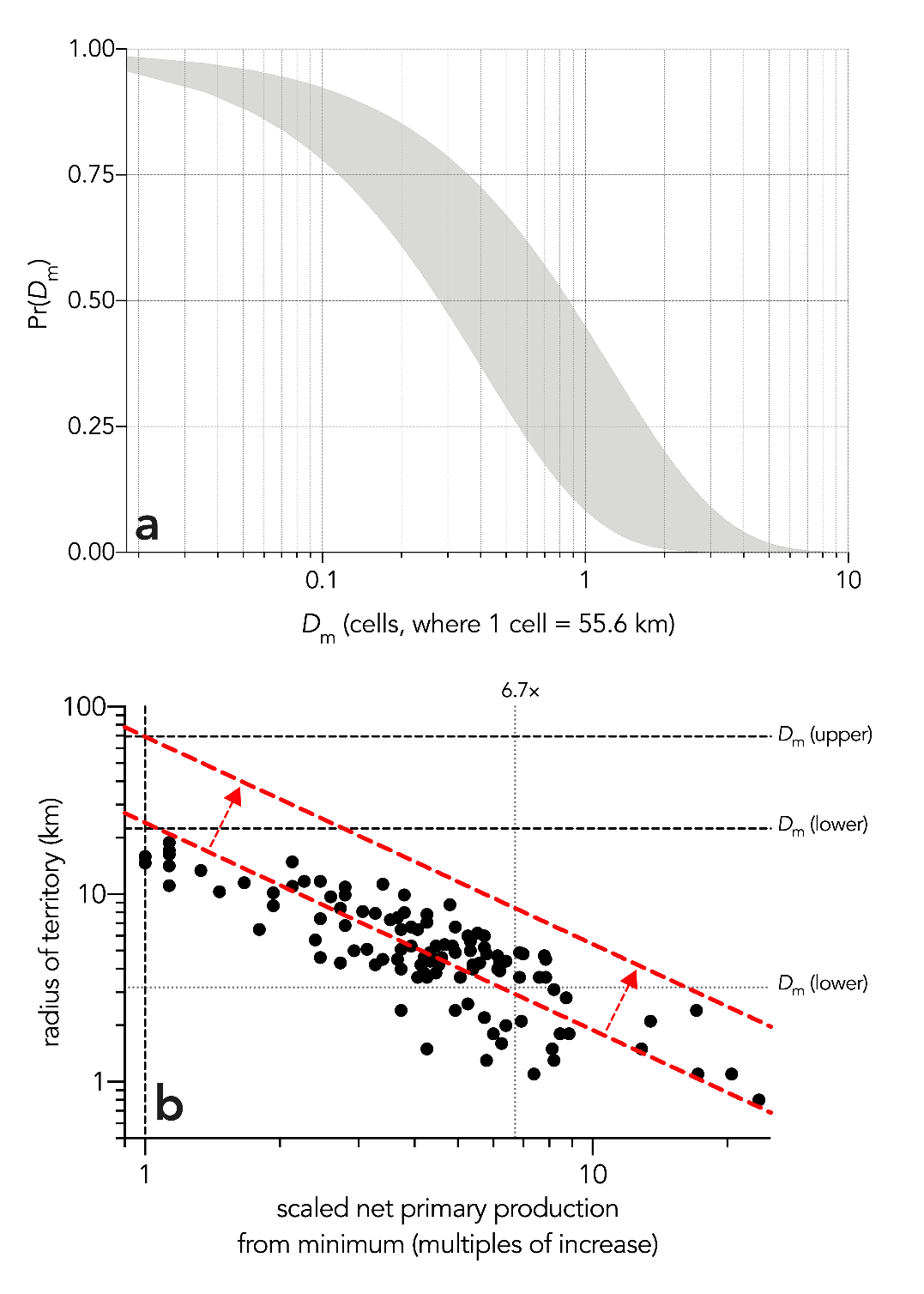


**Supplementary Fig. 8** Relationship between the proportional change in movement probability as a function of elevational difference following the exponential decay function $M_{\text{red}}=a+b\sqrt[3]{G_{\text{rel}}}$, where *M*_red_ = the proportion of expected total annual movement, *a* = 1.001116, *b* = -0.0104453, and *G*_rel_ = the standardized ruggedness from 0 to 1. This reduces annual movement rates up to a maximum of 1%.


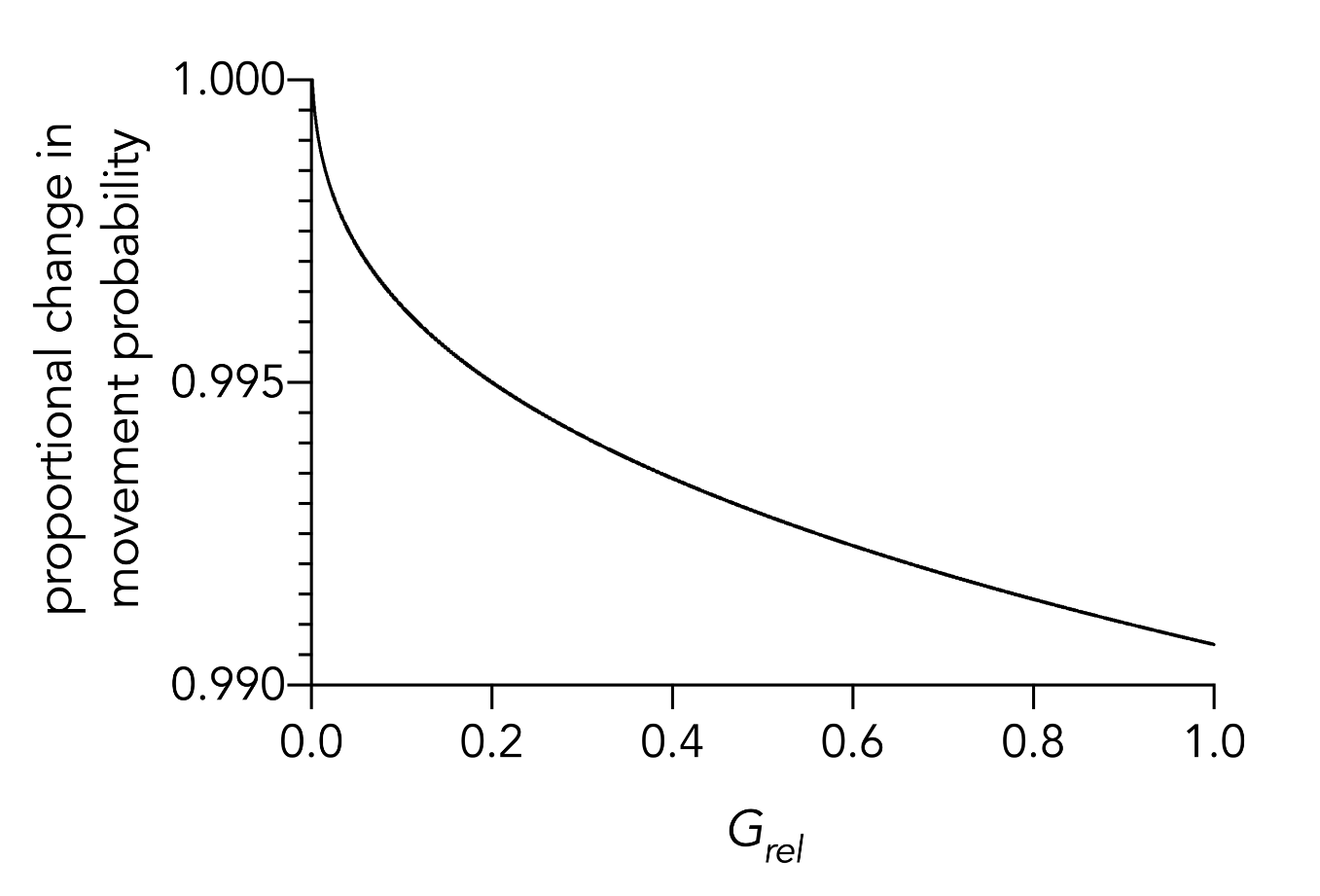


**Supplementary references**

1 Williams, A. N., Ulm, S., Smith, M. & Reid, J. AustArch: a database of ^14^C and non-^14^C ages from archaeological sites in Australia - composition, compilation and review. *Intern Archaeol* **36**, doi:10.11141/ia.36.6 (2014).

2 Clarkson, C. *et al.* Human occupation of northern Australia by 65,000 years ago. *Nature* **547**, 306-310, doi:10.1038/nature22968 (2017).

3 David, B. *et al.* 45,610–52,160 years of site and landscape occupation at Nawarla Gabarnmang, Arnhem Land plateau (northern Australia). *Quat. Sci. Rev.* **215**, 64-85, doi:10.1016/j.quascirev.2019.04.027 (2019).

4 Langley, M. C., O'Connor, S. & Aplin, K. A >46,000-year-old kangaroo bone implement from Carpenter's Gap 1 (Kimberley, northwest Australia). *Quat. Sci. Rev.* **154**, 199-213, doi:10.1016/j.quascirev.2016.11.006 (2016).

5 Maloney, T., O'Connor, S., Wood, R., Aplin, K. & Balme, J. Carpenters Gap 1: a 47,000 year old record of indigenous adaption and innovation. *Quat. Sci. Rev.* **191**, 204-228, doi:10.1016/j.quascirev.2018.05.016 (2018).

6 Wood, R. *et al.* Towards an accurate and precise chronology for the colonization of Australia: the example of Riwi, Kimberley, Western Australia. *PLoS One* **11**, e0160123, doi:10.1371/journal.pone.0160123 (2016).

7 Veth, P. *et al.* Minjiwarra: archaeological evidence of human occupation of Australia’s northern Kimberley by 50,000 BP. *Aust. Archaeol.* **85**, 115-125, doi:10.1080/03122417.2019.1650479 (2019).

8 Veth, P. *et al.* Early human occupation of a maritime desert, Barrow Island, North-West Australia. *Quat. Sci. Rev.* **168**, 19-29, doi:10.1016/j.quascirev.2017.05.002 (2017).

9 McDonald, J. *et al.* Karnatukul (Serpent’s Glen): a new chronology for the oldest site in Australia’s Western Desert. *PLoS One* **13**, e0202511, doi:10.1371/journal.pone.0202511 (2018).

10 Cropper, D. & Law, W. B. Rockshelter Excavations in the East Hamersley Range, Pilbara Region, Western Australia 454 (Archaeopress, Oxford, United Kingdom, 2018).

11 Marsh, M., Hiscock, P., Williams, D., Hughes, P. & Sullivan, M. Watura Jurnti: a 42000–45000-year-long occupation sequence from the north-eastern Pilbara. *Archaeol. Oceania* **53**, 137-149, doi:10.1002/arco.5152 (2018).

12 Morse, K., Cameron, R. & Reynen, W. A tale of three caves: new dates for Pleistocene occupation in the inland Pilbara. *Aust. Archaeol.* **79**, 167-178, doi:10.1080/03122417.2014.11682033 (2014).

13 Reynen, W., Vannieuwenhuyse, D., Morse, K., Monks, C. & Balme, J. What happened after the Last Glacial Maximum? Transitions in site use on an arid inland island in north-western Australia. *Archaeol. Oceania* **53**, 150-162, doi:10.1002/arco.5155 (2018).

14 Reynen, W. *Rockshelters and Human Mobility during the Last Glacial Maximum in the Pilbara Uplands, North-Western Australia* PhD thesis, University of Western Australia, (2018).

15 Slack, M. J., Law, W. B. & Gliganic, L. A. Pleistocene settlement of the eastern Hamersley Plateau: a regional study of 22 rock-shelter sites. *Archaeol. Oceania* **53**, 191-204, doi:10.1002/arco.5163 (2018).

16 Hamm, G. *et al.* Cultural innovation and megafauna interaction in the early settlement of arid Australia. *Nature* **539**, 280-283, doi:10.1038/nature20125 (2016).

17 Sherwood, J. E. *et al.* The Moyjil site, south-west Victoria, Australia: chronology. *Proc R Soc Vic* **130**, 32-49, doi:10.1071/RS18005 (2019).

18 Walshe, K. Port Augusta hearth site dated to 40,000 years. *Aust. Archaeol.* **74**, 106-110, doi:10.1080/03122417.2012.11681940 (2012).

19 Dias, A. & Rapley, S. New radiocarbon dates from the Chichester Range, Pilbara, Western Australia. *J. Aust. Assoc. Consult. Archaeol.* **2**, 9-14 (2014).

20 O'Connell, J. F. & Allen, J. The process, biotic impact, and global implications of the human colonization of Sahul about 47,000 years ago. *J. Archaeol. Sci.* **56**, 73-84, doi:10.1016/j.jas.2015.02.020 (2015).

21 Chappell, J. *et al.* Reconciliation of late Quaternary sea levels derived from coral terraces at Huon Peninsula with deep sea oxygen isotope records. *Earth Planet. Sci. Lett.* **141**, 227-236, doi:10.1016/0012-821X(96)00062-3 (1996).

22 Fairbairn, A. S., Hope, G. S. & Summerhayes, G. R. Pleistocene occupation of New Guinea's highland and subalpine environments. *World Archaeol.* **38**, 371-386, doi:10.1080/00438240600813293 (2006).

23 Gosden, C. & Robertson, N. in *Report of the Lapita Homeland Project. Occasional Papers in Prehistory 20* (eds J. Allen & C. Gosden) 20-45 (Department of Prehistory, Research School of Pacific Studies, Australian Natitonal Universitty, 1991).

24 Groube, L., Chappell, J., Muke, J. & Price, D. A 40,000 year-old human occupation site at Huon Peninsula, Papua New Guinea. *Nature* **324**, 453-455, doi:10.1038/324453a0 (1986).

25 Leavesley, M. & Allen, J. Dates, disturbance and artefact distributions: another analysis of Buang Merabak, a Pleistocene site on New Ireland, Papua New Guinea. *Archaeol. Oceania* **33**, 63-82, doi:10.1002/j.1834-4453.1998.tb00405.x (1998).

26 Leavesley, M. G. *et al.* Buang Merabak: early evidence for human occupation in the Bismarck Archipelago, Papua New Guinea. *Aust. Archaeol.* **54**, 55-57, doi:10.1080/03122417.2002.11682070 (2002).

27 Pavlides, C. & Gosden, C. 35,000-year-old sites in the rainforests of West New Britain, Papua New Guinea. *Antiquity* **68**, 604-610, doi:10.1017/S0003598X00047104 (1994).

28 Roberts, R. G. Luminescence dating in archaeology: from origins to optical. *Rad. Measure.* **27**, 819-892, doi:10.1016/S1350-4487(97)00221-7 (1997).

29 Summerhayes, G. R. *et al.* Human adaptation and plant use in highland New Guinea 49,000 to 44,000 years ago. *Science* **330**, 78, doi:10.1126/science.1193130 (2010).

30 Torrence, R. *et al.* Pleistocene colonisation of the Bismarck Archipelago: new evidence from West New Britain. *Archaeol. Oceania* **39**, 101-130, doi:10.1002/j.1834-4453.2004.tb00568.x (2004).

31 O’Connor, S. *et al.* The power of paradigms: examining the evidential basis for early to Mid-Holocene pigs and pottery in Melanesia. *Pac. Archaeol.* **2**, 1-25 (2011).

32 Rodríguez-Rey, M. *et al.* Criteria for assessing the quality of Middle Pleistocene to Holocene vertebrate fossil ages. *Quat. Geochronol.* **30**, 69-79, doi:10.1016/j.quageo.2015.08.002 (2015).

33 Bird, M. I. *et al.* The efficiency of charcoal decontamination for radiocarbon dating by three pre-treatments – ABOX, ABA and hypy. *Quat. Geochronol.* **22**, 25-32, doi:10.1016/j.quageo.2014.02.003 (2014).

34 Alex, B. *et al.* Radiocarbon chronology of Manot Cave, Israel and Upper Paleolithic dispersals. *Sci. Adv.* **3**, e1701450, doi:10.1126/sciadv.1701450 (2017).

35 Fifield, L. K. *et al.* Radiocarbon dating of the human occupation of Australia prior to 40 ka BP—successes and pitfalls. *Radiocarbon* **43**, 1139-1145, doi:10.1017/S0033822200041795 (2001).

36 Reimer, P. J. *et al.* IntCal13 and Marine13 radiocarbon age calibration curves 0–50,000 years cal BP. *Radiocarbon* **55**, 1869-1887, doi:10.2458/azu_js_rc.55.16947 (2013).

37 Bronk Ramsey, C. Bayesian analysis of radiocarbon dates. *Radiocarbon* **51**, 337-360, doi:10.1017/S0033822200033865 (2009).

38 Clarkson, C. *et al.* Reply to comments on Clarkson et al. (2017) ‘Human occupation of northern Australia by 65,000 years ago’. *Aust. Archaeol.* **84**, 84-89, doi:10.1080/03122417.2018.1462884 (2018).

39 Veth, P. Breaking through the radiocarbon barrier: Madjedbebe and the new chronology for Aboriginal occupation of Australia. *Aust. Archaeol.* **83**, 165-167, doi:10.1080/03122417.2017.1408543 (2017).

40 Bowdler, S. ‘Human occupation of northern Australia by 65,000 years ago’ (Clarkson et al. 2017): a discussion. *Aust. Archaeol.* **83**, 162-163, doi:10.1080/03122417.2017.1408198 (2017).

41 Hiscock, P. Discovery curves, colonisation and Madjedbebe. *Aust. Archaeol.* **83**, 168-171, doi:10.1080/03122417.2017.1408544 (2017).

42 Wood, R. Comments on the chronology of Madjedbebe. *Aust. Archaeol.* **83**, 172-174, doi:10.1080/03122417.2017.1408545 (2017).

43 Dortch, J. & Malaspinas, A.-S. Madjedbebe and genomic histories of Aboriginal Australia. *Aust. Archaeol.* **83**, 174-177, doi:10.1080/03122417.2017.1408546 (2017).

44 O’Connell, J. F. *et al.* When did *Homo sapiens* first reach Southeast Asia and Sahul? *Proc. Natl. Acad. Sci. U.S.A.* **115**, 8482, doi:10.1073/pnas.1808385115 (2018).

45 Saltré, F. *et al.* Climate-human interaction associated with southeast Australian megafauna extinction patterns. *Nat. Comm.* **10**, 5311, doi:10.1038/s41467-019-13277-0 (2019).

46 Hiscock, P. *Archaeology of Ancient Australia*. (Routledge, 2008).
