# Supplementary material for: Stochastic models support rapid peopling of Late Pleistocene Sahul
Source: Nat Commun. 2021 Apr 29;12:2440. doi: 10.1038/s41467-021-21551-3 (PMC8085232; doi:10.1038/s41467-021-21551-3)
Supplement: Supplementary file 3 — Reporting Summary [file 41467_2021_21551_MOESM3_ESM.pdf]

nature research

Corresponding author(s): Corey J. A. Bradshaw

Last updated by author(s): Jan 21, 2021

Reporting Summary

Nature Research wishes to improve the reproducibility of the work that we publish. This form provides structure for consistency and transparency in reporting. For further information on Nature Research policies, see our Editorial Policies and the Editorial Policy Checklist.

Statistics

For all statistical analyses, confirm that the following items are present in the figure legend, table legend, main text, or Methods section.

n/a	Confirmed	
☐	☒	The exact sample size (*n*) for each experimental group/condition, given as a discrete number and unit of measurement
☒	☐	A statement on whether measurements were taken from distinct samples or whether the same sample was measured repeatedly
☐	☒	The statistical test(s) used AND whether they are one- or two-sided<br>*Only common tests should be described solely by name; describe more complex techniques in the Methods section.*
☐	☒	A description of all covariates tested
☐	☒	A description of any assumptions or corrections, such as tests of normality and adjustment for multiple comparisons
☐	☒	A full description of the statistical parameters including central tendency (e.g. means) or other basic estimates (e.g. regression coefficient) AND variation (e.g. standard deviation) or associated estimates of uncertainty (e.g. confidence intervals)
☐	☒	For null hypothesis testing, the test statistic (e.g. F, t, r) with confidence intervals, effect sizes, degrees of freedom and P value noted<br>*Give P values as exact values whenever suitable.*
☒	☐	For Bayesian analysis, information on the choice of priors and Markov chain Monte Carlo settings
☒	☐	For hierarchical and complex designs, identification of the appropriate level for tests and full reporting of outcomes
☐	☒	Estimates of effect sizes (e.g. Cohen's d, Pearson's r), indicating how they were calculated

Our web collection on statistics for biologists contains articles on many of the points above.

Software and code

Policy information about availability of computer code

Data collection	all data available at doi:10.5281/zenodo.4453767)
Data analysis	All code written in R 3.5.2 (provided at doi:10.5281/zenodo.4453767). R packages used: sp, rgdal v1.5-16, raster v3.3-13, oceanmap v0.1.1, insol v1.2.1, OceanView v1.0.5, abind v1.4-5, pracma v2.2.9, binford v0.1.0, rgl v0.100.54, scatterplot3d, spatstat, spatialEco v1.3-1, SpatialPack v0.3-819, doSNOW v1.0.18, iterators v1.0.12, snow v0.4-3, foreach v1.5.0, lhs v1.0.2, data.table v1.13.0 (all packages available: https://CRAN.R-project.org). Also used Oxcal 4.4 (https://c14.arch.ox.ac.uk/oxcal/OxCal.html) to calibrate radio carbon dates to calendar years.

For manuscripts utilizing custom algorithms or software that are central to the research but not yet described in published literature, software must be made available to editors and reviewers. We strongly encourage code deposition in a community repository (e.g. GitHub). See the Nature Research guidelines for submitting code & software for further information.

Data

Policy information about availability of data

All manuscripts must include a data availability statement. This statement should provide the following information, where applicable:

- Accession codes, unique identifiers, or web links for publicly available datasets
- A list of figures that have associated raw data
- A description of any restrictions on data availability

data included as .csv files at doi:10.5281/zenodo.4453767: 1. distance to water (Australian Water Observations from Space dataset doi:10.1073/pnas.1608470113); net primary production (LOVECLIM output doi:10.5194/gmd-3-603-2010); ruggedness (Riley, S. J., DeGloria, S. D. & Elliot, R. A terrain ruggedness index that quantifies topographic heterogeneity. Intermount. J. Sci. 5:23-27,1999); palaeo sea level & lake fill (ETOPO1 https://catalog.data.gov/dataset/etopo1-1-arc-minute-global-relief-model & doi:10.1038/ncomms6076); rainfall & territory size (Hiscock, P. Archaeology of Ancient Australia. Routledge, 2008); human life tables

(doi:10.1038/s41559-019-0902-6). Additional archaeological data provided from AustArch database (doi:10.11141/ia.36.6) supplemented with data published in the relevant literature (described in SI material).

Field-specific reporting

Please select the one below that is the best fit for your research. If you are not sure, read the appropriate sections before making your selection.

- [ ] Life sciences
- [ ] Behavioural & social sciences
- [x] Ecological, evolutionary & environmental sciences

For a reference copy of the document with all sections, see nature.com/documents/nr-reporting-summary-flat.pdf

Ecological, evolutionary & environmental sciences study design

All studies must disclose on these points even when the disclosure is negative.

Study description	We construct a cellular-automaton model to test hypotheses regarding the entry points, sequency of entry, timing of entry, and relationship to carrying capacity for the first anatomically modern humans to people Sahul.
Research sample	All modelled (simulated) results; however, we include data from LOVECLIM (climate hindcast model), hindcasted sea level, anthropological relationships for dispersal, permanent water distribution, landscape ruggedness, and archaeological data as a comparison layer. Data sources described above.
Sampling strategy	We sampled data for each cell of Sahul at a 0.5 degree x 0.5 degree resolution based on the minimum resolution of the datasets described above.
Data collection	S. Ulm and A. Williams collated all archaeological data; F. Saltré & T. Friedrich provided all LOVECLIM data and outputs by running the LOVECLIM model; F. Saltré generated lake and sea level data from the ETOPO1 product; F Saltré downscaled both LOVECLIM net primary production and ruggedness data; M. Bird provided distance to water data from previous work. C. Bradshaw provided human demographic data from previous work.
Timing and spatial scale	Collection of all data sources mentioned occurred between November 2018 and December 2019. Archaeological data were supplemented with newly published data in June 2020.
Data exclusions	Archaeological date data excluded if dated younger than 30,000 years BP, and if radiocarbon dates were below a 'A' rating (using technique modified from doi:10.1016/j.quageo.2015.08.002)
Reproducibility	All simulations were repeated 100s to 1000s of times, with no simulation failures.
Randomization	Fully randomised (resampled) stochastic model
Blinding	The authors responsible for providing the underlying data, including the archaeological comparison data, were not involved in any aspect of the model coding or implementation. Their results were therefore not influenced by the model itself.

Did the study involve field work? [ ] Yes [x] No

Reporting for specific materials, systems and methods

We require information from authors about some types of materials, experimental systems and methods used in many studies. Here, indicate whether each material, system or method listed is relevant to your study. If you are not sure if a list item applies to your research, read the appropriate section before selecting a response.

Materials & experimental systems

n/a	Involved in the study
[x]	[ ] Antibodies
[x]	[ ] Eukaryotic cell lines
[x]	[ ] Palaeontology and archaeology
[x]	[ ] Animals and other organisms
[x]	[ ] Human research participants
[x]	[ ] Clinical data
[x]	[ ] Dual use research of concern

Methods

n/a	Involved in the study
[x]	[ ] ChIP-seq
[x]	[ ] Flow cytometry
[x]	[ ] MRI-based neuroimaging
