# Supplementary material for: Stochastic models support rapid peopling of Late Pleistocene Sahul
Source: Nat Commun. 2021 Apr 29;12:2440. doi: 10.1038/s41467-021-21551-3 (PMC8085232; doi:10.1038/s41467-021-21551-3)
Supplement: Supplementary file 4 — Description of Additional Supplementary Files [file 41467_2021_21551_MOESM4_ESM.pdf]

Description of Additional Supplementary Files

File name: Supplementary Data 1
Description: Reference archaeological age dataset for Sahul constructed by building on the *AustArch* compilation[1]. We selected 30,000 BP (years before present) for radiocarbon ages and 30,000 years ago for other techniques as an arbitrary threshold to encompass ages representing the earliest phases of human occupation of the continent. Quality-rated ages from archaeological deposits ≥ 30 ka. See *Compiling reference archaeological dates* for definitions. Note that 'AGE' refers to years BP for radiocarbon ages and years for other techniques.

References

1 Williams, A. N., Ulm, S., Smith, M. & Reid, J. AustArch: a database of ^{14}C and non-^{14}C ages from archaeological sites in Australia - composition, compilation and review. *Intern Archaeol* **36**, doi:10.11141/ia.36.6 (2014).
