# Supplementary material for: Stochastic models support rapid peopling of Late Pleistocene Sahul
Source: Nat Commun. 2021 Apr 29;12:2440. doi: 10.1038/s41467-021-21551-3 (PMC8085232; doi:10.1038/s41467-021-21551-3)
Supplement: Supplementary file 2 — Peer Review File [file 41467_2021_21551_MOESM2_ESM.pdf]

Reviewer #1 (Remarks to the Author):

This is a well-written manuscript, on a most interesting topic, i.e., the peopling of Sahul. As noted by the authors, Sahul was one of the earliest places on Earth that was solely occupied by migrating groups of modern human populations. The current group of authors have been writing on this extremely interesting topic of late, examining routes of entry into Sahul, the rate of movement, and the size of the dispersing populations. This article concerns a testing of these hypotheses through use of stochastic models. I am not a modelling expert, and I can not evaluate the modelling aspect of this paper. I did, however, carefully read the presentation, and I was able to follow the authors logic, which was nicely laid out. Through assembly of a range of data sets and analysis, the authors present a series of significant results and conclusions. They argue in favour of a entry point via the northwestern portion of the Sahul shelf and they find that the continent was probably occupied rapidly, meaning across ca. 5,000 years. I am particularly interested to see that they conclude that they entry could have been between 75-50 ka, consistent with arguments that Asia could have been originally colonised between 90-60 ka by H. sapiens, employing terrestrial routes, as opposed to a model for rapid coastal dispersals.

I have very little to quibble with in this article. One point I would like to make, however, is that one of the most important aspects underlying this model is archaeology. The early archaeological record of Sahul is pulled together nicely in the Supplementary Information. As noted by the authors, the archaeological data is crucial and underpins what is being done here, even if it is not entirely complete. I found Figure S1 to be of great importance, and I recommend that it be integrated as a fifth figure into the main text. The data behind this figure is of importance to the model, and it provides the basis, as the authors note, for future ground work, and I could not agree more with their final statement: "Improving and expanding the archaeological record in places of initial arrival and periods must therefore form a research priority to improve model predictions of the development of human society during the Late Pleistocene." I therefore believe the authors should consider integrating Figure S1 into the main text as it would clearly show the importance of the data that we need to prove the conclusions of the model as correct.

Reviewer #2 (Remarks to the Author):

The major claims of this paper are: 1. That the most likely date for the first entry of humans into Sahul are 50 Ka, (with 75 as a second option); 2. That from colonisation to reaching carrying capacity was rapid (~ 4000 - 6000 years); 3. That the mostly likely route was directly into NW Australia, rather than via New Guinea; and 4. That the equilibrium population was higher than previous estimates (6.3 - 6.5 million people).

This is a relatively well-established research question - the timing and routes of Australian colonisation, and the routes and dates proposed are approximately ones that have been extensively discussed as the most likely. The novelty of the paper lies in the approach. The paper is a purely modelling one, using a number of parameters to calculate patterns of dispersion. The key and novel elements of this one are ecological - that is, in addition to using the generally well-established route models, i.e. friction models or visual connectivity - emphasis is placed on the ecology, particularly productivity and carrying apacity (using spatially explicit modelling and time-corrected ecological values). These environmental parameters are tied in to human demographic ones, particularly residential mobility of hunter-gatherers (based on a number of previously published papers on hummer gatherer ecology and mobility).

The construction of the model is extremely impressive, as it treats with considerable sophistication a whole number of thorny issues in ecological modelling - for example, the tension between business as usual models of population growth and dispersion and the impact of stochastic and detrimental effects. The interaction of hunter-gatherer demography with the shifting levels of primary productivity

is also nicely done.

As ever, there remain issues with the paper that I think need to be addressed in a revision.
1. The major one is that the paper is written in such a way as to suggest that the model demonstrates what happened in Australian prehistory, rather than ascribing probabilistic values to scenarios, or ruling out others as infeasible. This is partly a matter of how it is written, but it also reflects the very low prominence in the main text given to the actual archaeological evidence. This is presented only as a series of Spearman correlations with a highly abstracted model of the Australian archaeological record. Impressive as the approach using the Signor-Lipps effect is, this is no substitute for actually looking hard at the evidence. The obvious, and recognised, issue is the site of Madjedbebe, which has a date at least 65 Ka, possibly older. Unless this site is rejected (unlikely as one of the authors excavated it), it seems to me that it constitutes one of Huxley's 'ugly facts' in the face of a beautiful model. As the difference in probability between the 50 Ka date and the earlier date entry is not that great, it would be worth paying more attention to the actual evidence, especially as it rules directly to their major claim.
2. In the same vein, another conclusion is the very rapid colonisation - 4000-6000 years in round figures. My experience in modelling population dispersals is that models tend to come up with very rapid rates - in practice, modelled populations tend to breed like rabbits, and spread very fast. The problem is getting the rates down to something that sort of makes common sense. It is possible that these were the rates, and that is the period of time to saturation, but either this needs to be assessed more in the context of very rapidly expanding invasive species (which do occur), or there needs to be a bit more sensitivity testing to the relationship with carrying apacity, or the level at which carrying capacity is set.
3. I have no particular quarrel with the southern point of entry, but Malaspinas et al, in their paper on the genomics of Australia, suggest that the Sahulian population entered as a a single clade, but diverged quickly afterwards. While that is feasible from the southern entry, the geographical patterns of relatedness between Australians and Papuans needs to be accounted for. I should add, in fairness, that the Malaspinas genomic dates accord well with the preferred 50 Ka scenario in terms on chronology, even if they do not geographically.
4. Perhaps the most surprising - but not impossible - claim in the paper is the proposed population of Australia at demographic equilibrium - presumably before 40 Ka, if I understand the paper correctly, but certainly in the Pleistocene. Over 6 million people is a very large estimated number. Previous estimates have varied considerably, but as far as I know none has exceeded 2 million (certainly for the Pleistocene), and most are considerably less, seldom exceeding 1 million. While there is some discussion of this in the text, one cannot help wondering if this number reflects a problem with the model.

I am a fan of modelling approaches, as I think one of the biggest problems in most archaeological work has been the absence of properly quantified expectations. I think this is a very promising approach and paper, and would like to see a version of it published. However, I think some further revisions or testing of the parameters are required to address the issues outlined above. I always think models are at their most interesting where they highlight anomalies, and these are what could very positively be assessed.

I have not checked the code or statistical work, which would require specialist assessment.

Reviewer #3 (Remarks to the Author):

Stochastic models reveal rapid early peopling of Late Pleistocene Sahul
Corey J. A. Bradshaw, Kasih Norman, Sean Ulm, Alan N. Williams, Chris Clarkson, Joël Chadoeuf, Sam C. Lin, Zenobia Jacobs, Richard G. Roberts, Michael I. Bird, Laura S. Weyrich, Simon G. Haberle, Sue

O’Connor, Bastien Llamas, Tim J. Cohen, Tobias Friedrich, Peter Veth, Matthew Leavesley, Frédérik Saltré

This interesting paper attempts to assess the fit between alternative models of colonization and spread through the Sahul and the archaeological data, while considering ecological/climatic factors. The study is an impressive exercise along two lines of exploration – (1) of utilizing an array of inference methods and bias corrections for the archaeological data, and (2) the implementation of generative models that simulate alternative colonization and spread dynamics across a realistic explicit spatial grid.
I am generally skeptical regarding the possibility to precisely infer a value for singular events in systems that involve so many moving parts (in this case: exactly the when and the where of the colonization).
Models that consider multiple interacting dynamics like this one are super-useful to get a handle on order-of-magnitude of timescales of processes, to demonstrate feasibility of alternative qualitative dynamics, to assess the relative importance of different parameters and processes in the larger picture, and to fit parameters of recurring processes (say, for example, the rate of migration between populations, or the rate and distribution of effect-sizes of adaptations, or even the effective size of a population bottleneck which is in reality a convolution of dozens of demographic fluctuations of different magnitudes, and so on).
However, testing with such models whether a scenario of colonization at 75 kya or one that occurred at 50kya is more likely, seems to me over-ambitious when there are so many unknowns. This study didn’t convince me otherwise.
I offer below my comments, in hope that this helps improve future versions of the manuscript.

• It is a bit odd to test only 5 or 7 scenarios (time-wise), get nearly the highest-possible support for the latest date tested, and then have the title use the wording “models reveal rapid early peopling…” in the title.
• Population size estimates are also odd: they are “baked” into the model to a great extent by the parameters of carrying capacity; they don’t rely on any archaeological data (I think), and they aren’t tested in comparison to archaeological data. Thus, the result that the model predicts larger population sizes than previously considered is primarily a model assumption, and not a result, isn’t it?
• The reason that previous studies considered smaller population sizes is that most studies do not assume that paleolithic carrying capacities align very well (perhaps due to cultural complexity, or maybe other factors) with present-day hunter-gatherer societies. So the difference between this study and others seems to stem from the assumptions, not the results (I’m agnostic regarding which is correct; just pointing this out).
• The assumptions regarding mortality/reproduction rates due to minimum viable population size seem to me unwarranted. A population size suffers from inbreeding below a certain effective population size in the long run, after equilibrating to the genetic diversity that is characteristic of such a small populations size. A population of 100 individuals that is sampled at random from a 10,000-individual population size will have heterozygosity and even allelic diversity that is only slightly skewed compared to the original population size, and very very far from the genetic diversity that characterizes an N=100 population. It would take dozens or hundreds of generations (on the order of the whole time of the simulation) for heterozygosity to drop to the low levels that characterize N=100 at equilibrium and for inbreeding depression to kick in.
• There is no statistical significance on anything. This is fine, but there also isn’t an alternative statistical approach that would help tell apart what differences are more or less reliable (e.g. some kind of Bayesian analysis, or other non-frequentist approach). It seems from the error bars in figure S2 that more than half of the scenarios tested have fit quality that are not different from one another in a statistically significant way.
• I suddenly realized – the best fits of the model and the data have a rho on the order of 0.15? That doesn’t sound very high. Maybe I misunderstand. Perhaps add an explanation.
• It is extremely annoying to read the results with the figures not embedded in the appropriate place in the text. Nat Comms allows submission with embedded figures and captions. An annoyed reviewer

is a less constructive one.
• Figure S1, which is crucially important for understanding what the main findings are based on, is in the supplementary, and very hard to understand. A legend would help a lot, and clearer explanation and marking of what each isohyet means would help. Are they currently at numbers like "24,380 kya" instead of round numbers? When the error bars around each estimate are on the order of magnitude of hundreds or thousands of years? Since you have a full topographic map of the whole "landscape of timing", you can choose which isohyets to mark…
• Also, are they estimated using dating methods as an absolute date, and then translated to a date relative to an inferred date of first arrival, which is the greatest unknown in the study? Or did I misunderstand? Either way – probably deserves some clarification.
• This is a bit of a blasphemous question, but – how much does the modeling exercise add to a direct estimate of time-of-arrival and of rate-of-spread that are assessed directly from the archaeological data? That is, if you just take the signor-Lipps-spatial-biased-corrected map, what is the earliest date that appears there, and does it point to the same primary introduction point that the study currently highlight? And is the second-earliest date at 50kya, near the northern inferred introduction point? In other words, is the most common-sense timing+route of introduction, based on the corrected map, the same timing+route that the complicated simulations lead to? If yes, perhaps clarify this, and also discuss what added value the simulations have? (they definitely have a cost in the form of relying on 10-20 assumptions that are all questionable).
• I suddenly realized that you estimate that the colonization of the whole continent happened within 5000 years, which would be 20,000 years before the second arrival that you suggest is likely at the north site, if the first colonization was in the western site at 75,000. That doesn't seem to make sense.
• Which leads to the question: did you penalize the model for added complexity? A model that allows more than one entry point would necessarily be able to produce a better fit, as it has an added level of freedom. If you did, how? If not, why? Either way – worth discussion.
• The model prediction that the arid region would have been a barrier for movement is, again, not a result – it's an assumption, baked into the model through the carrying capacity calculation.
• Modeling dispersal – not sure how exactly you implemented dispersal, but simplistically you could imagine that a group decides to bud off, and that this decision is not effected by the availability of nearby unsettled territory or nearby water resources, and only secondarily the group decides where to go while taking into account these considerations, or alternatively you could have the availability of vacant and close-to-water routes/land be baked into the calculation of whether to immigrate at all. The choice among these (and similar considerations) could lead to huge differences in the emergent dynamics.
• The same is true regarding distance of dispersal – you can view it as a pre-determined coin-toss, or you can imagine that a group continues on and on until they find an appropriate place to stay for the longer run (the "on and on" can play out within years or across a few generations). The latter option might lead to non-intuitive results, e.g. that deserts are actually crossed much faster than expected, and that spread through zones of intermediate bio-productivity might be way faster than zones with high productivity (even under the linear K-biodiversity relation curve).
• I think I wrote this, but modeling dispersal distances and rates of an "invading" species that is expanding its range into unoccupied territory (unoccupied by another similar organism, that is), based on data estimates for populations of animals and hunter-gatherers that are at a steady-state might be extremely misleading, possibly hitting off-target by orders of magnitude. At least discuss this.
• Global scale conclusions – no support for coastal-migration preference: of course the model here didn't support such preference; the assumptions that the idea's proponents highlight that would make such a scenario feasible are not considered in the model!
(to name some - consistent supply of same-type resources that are readily accessible along the coast and don't require adaptation to new methods of hunting/gathering/handling, ease of movement along the coast – by foot along the coastal plane and/or by means of water-transportation).
• Calculating a minimal global arc and fitting its length to the mean rate of per-generation movement that the model suggests seems extremely simplistic and misleading. At the least, it almost sounds like it implies that humans knew how to take the shortest route to the Sahul, and that that's where they

were going. The hyper-sensitivity of this argument to deviations seems obvious (e.g. what if the mean rate of movement is twice or three times faster? Or twice slower? What are the confidence interval with which you estimate the 0.71-0.92 km/year rate, if you consider slight alterations to the more arbitrary among the model assumptions?)

• I don't think the model adds to the current knowledge about "adaptability and technologically advanced state of anatomically modern humans" – that isn't what the model models, and any results along these lines are direct reflection of the assumptions, not an emergent result. Other papers (by some of the same authors) do perhaps support some of these statements, but this model doesn't lend any additional support, as far as I can tell.

• The same is true regarding the population sizes, as noted earlier. The finding that might support the idea that these weren't small unorganized bands but a larger and more advanced population have to do with the number of individuals that arrived at the same time (nearly) and things like that – but these are results of some of the authors' previous studies, and this one relies on them – it doesn't add any independent support as far as I can tell.

• (I'm agnostic regarding whether these claims are correct; I find them very reasonable, I like to think they are true, and I find some previous papers from these authors about these questions convincing – I'm merely pointing out that these aren't results of the model in this study, as they are presented)

• Model sensitivity section: I don't think this is what I'd consider a sensitivity analysis. This is an important assessment of the importance of the different processes and parameters, but it doesn't explore how sensitive the results are (or, at least it doesn't report to the reader) to changes to these parameters. Sensitivity analysis should answer the question: what changes, in which parameters, would lead to a qualitatively different main result/s. In this case, I'd like a sensitivity analysis to include, for example, an exploration of how much would the estimate of colonization timing and length change if the long-distance-migration distance were, say, 5 times larger than the one considered (a scale of long-distance dispersal that is clearly within a reasonable range, given the references mentioned for human yearly mobility info). (this is just an example, of course. The same is true for each of the parameters, particularly those that are highlighted as most influential).

Moreover, a sensitivity analysis might also be expected to explore breaking of the slightly-more-fundamental model assumptions. For example, the maximal migration distance in the model is 10 cells (55km each). If I understand correctly, this is undertaken maximum once per generation in the simulation. However the citation from Gould suggests that this scale of movement might occur within a single bout, leaving open the possibility that some nomadic hunter-gatherers might have done this on a much more regular basis than once per generation. The wave of colonization isn't determined by the mean of the population, it is determined by the front – those few outliers, possibly even an "outlier culture" that moves fastest. Cane toads in Australia – not a very rapidly moving organism compared to some mammals – seem to be spreading PER YEAR at a rate that is very close to the far-right edge of the considered per-generation distribution; perhaps dispersal/long-distance-migration in an ecosystem with available niches is much much faster than in saturated ecosystems, from which the parameter estimates used in this study were derived?

Revision Summary

We reply to each of the reviewers' comments in detail below, but we summarise our main revisions and responses here:

- We have completely redone the global sensitivity analysis, mainly to address the concerns about maximum dispersal distance (our original conclusions are robust).
- We have provided more simulations testing the effect of increasing dispersal distance (our original conclusions remain unchanged).
- We now show the magnitude of the effect of the parameters deemed to influence time to continental saturation from the global sensitivity analysis.
- We demonstrate that nothing is "baked in" in this fully stochastic model, and that archaeological data provide the basis with which to compare all model predictions.
- We have now provided all archaeological data we used to derive the comparison map layers in the Supplementary Information.
- We elucidate the statistical methodology and interpretation of the correlations between the simulations and archaeological map layer.
- We clarify that all population estimates represent maximum potential, but that they are also consistent with measured population densities for hunter-gatherer societies.
- We show that we have already considered our results with and without a potentially controversial site (Madjedbebe).
- We reiterate that no other study has ever come close to this degree of sophistication for modelling ancient human spread across any landscape, let alone Sahul.

Reviewer #1

This is a well-written manuscript, on a most interesting topic, i.e., the peopling of Sahul. As noted by the authors, Sahul was one of the earliest places on Earth that was solely occupied by migrating groups of modern human populations. The current group of authors have been writing on this extremely interesting topic of late, examining routes of entry into Sahul, the rate of movement, and the size of the dispersing populations. This article concerns a testing of these hypotheses through use of stochastic models. I am not a modelling expert, and I can not evaluate the modelling aspect of this paper. I did, however, carefully read the presentation, and I was able to follow the authors logic, which was nicely laid out. Through assembly of a range of data sets and analysis, the authors present a series of significant results and conclusions. They argue in favour of a entry point via the northwestern portion of the Sahul shelf and they find that the continent was probably occupied rapidly, meaning across ca. 5,000 years. I am particularly interested to see that they conclude that they entry could have been between 75-50 ka, consistent with arguments that Asia could have been originally colonised between 90-60 ka by H. sapiens, employing terrestrial routes, as opposed to a model for rapid coastal dispersals.

I have very little to quibble with in this article. One point I would like to make, however, is that one of the most important aspects underlying this model is archaeology. The early archaeological record of Sahul is pulled together nicely in the Supplementary Information.

As noted by the authors, the archaeological data is crucial and underpins what is being done here, even if it is not entirely complete. I found Figure S1 to be of great importance, and I recommend that it be integrated as a fifth figure into the main text. The data behind this figure is of importance to the model, and it provides the basis, as the authors note, for future ground work, and I could not agree more with their final statement: "Improving and expanding the archaeological record in places of initial arrival and periods must therefore form a research priority to improve model predictions of the development of human society during the Late Pleistocene." I therefore believe the authors should consider integrating Figure S1 into the main text as it would clearly show the importance of the data that we need to prove the conclusions of the model as correct.

RESPONSE #1: We have now made Fig. S1 a main-text figure (new Fig. 1).

Reviewer #2

The major claims of this paper are: 1. That the most likely date for the first entry of humans into Sahul are 50 Ka, (with 75 as a second option); 2. That from colonisation to reaching carrying capacity was rapid (~ 4000 - 6000 years); 3. That the mostly likely route was directly into NW Australia, rather than via New Guinea; and 4. That the equilibrium population was higher than previous estimates (6.3 - 6.5 million people).

This is a relatively well-established research question - the timing and routes of Australian colonisation, and the routes and dates proposed are approximately ones that have been extensively discussed as the most likely.

RESPONSE #2: We agree that the research question itself has been discussed a lot, and many different conceptual models have been proposed. However, there are few (if any) actual tests of the question itself. Up until our paper, suppositions regarding entry points and the ensuing patterns of movement have only been surmised from logical plausibility[1-4] (i.e., conceptual 'models'), assessments of hypothesised routes for relative ease of arrival[5-9], archaeological arguments[10-13], point estimates of genetic divergence[14-16], or even putative hydrological connectivity[17]. In other words, all existing literature only proposes routes of movement, or draws arbitrary lines on maps between dated archaeological or genetic samples.

We have specifically avoided doing this by constructing a *stochastic* model based on human-ecological rules to discover the emergent property of peopling patterns without presupposing particular pathways lacking direct evidence. To these emergent (unguided, not predisposed) patterns, we compared the highest-quality archaeological evidence and, also for the first time, spatially and taphonomically corrected the biases inherent in such data to provide a form of relative validation.

We emphasise that no such analysis exists for this much-discussed, but rarely tested, phenomenon — we provide the first such test in our manuscript.

The novelty of the paper lies in the approach. The paper is a purely modelling one, using a number of parameters to calculate patterns of dispersion. The key and novel elements of this

one are ecological - that is, in addition to using the generally well-established route models, i.e. friction models or visual connectivity - emphasis is placed on the ecology, particularly productivity and carrying apacity (using spatially explicit modelling and time-corrected ecological values). These environmental parameters are tied in to human demographic ones, particularly residential mobility of hunter-gatherers (based on a number of previously published papers on hummer gatherer ecology and mobility).

The construction of the model is extremely impressive, as it treats with considerable sophistication a whole number of thorny issues in ecological modelling - for example, the tension between business as usual models of population growth and dispersion and the impact of stochastic and detrimental effects. The interaction of hunter-gatherer demography with the shifting levels of primary productivity is also nicely done.

> **RESPONSE #3**: This is not strictly correct. While certainly a model, it is not one which relies on presupposing any underlying spatial patterns (see Response #2). In fact, it relies mainly on measured parameters or well-established ecological principles to infer a spatially emergent property of expansion across the entire landscape of Sahul. It is also the first to incorporate a temporally and spatially dynamic expectation of human carrying capacity. More importantly, the model is anchored to real archaeological data, thereby providing a mechanism to rank scenario likelihood (again, without presupposing any scenario as more or less realistic, except in terms of limiting the entry times, points, sequence, and $K \sim P_p$ relationships to plausible ranges — see also Response #13).

As ever, there remain issues with the paper that I think need to be addressed in a revision. 1. The major one is that the paper is written in such a way as to suggest that the model demonstrates what happened in Australian prehistory, rather than ascribing probabilistic values to scenarios, or ruling out others as infeasible. This is partly a matter of how it is written, but it also reflects the very low prominence in the main text given to the actual archaeological evidence. This is presented only as a series of Spearman correlations with a highly abstracted model of the Australian archaeological record. Impressive as the approach using the Signor-Lipps effect is, this is no substitute for actually looking hard at the evidence.

> **RESPONSE #4**: We respectfully disagree. We have been careful to contextualise every model prediction in terms of relative weight of evidence, probability, and likelihood. That said, we have now better emphasised the probabilistic framework throughout the manuscript to avoid any accusation of stating prediction as fact — after all, "All models are wrong, but some are useful" (the late George Box, 1976), which means that models are necessary simplifications of a chaotic and complex universe that attempt to extract the most important elements driving the system toward particular outcomes or phenomena.
>
> We also highlight that archaeological data form a prominent and important role in this paper — data that validate the entire process. As such, the reviewer saying that "archaeology is not considered" is erroneous (also see Response #2).
>
> In terms of "hard evidence", one must be careful in assuming that a particular archaeological specimen's estimated date is of (a) high quality (i.e., is reliable), (b) taphonomically relevant, and (c) spatially representative. We argue that we have

indeed incorporated the "hard evidence" in the best possible manner because no single specimen can conceivably attest to the initial or terminal event due to sampling bias and taphonomic variability. This is the heart of the Signor-Lipps effect, to which archaeology is not immune. Had we *not* corrected for these biases (including the spatial bias imposed by non-uniform/non-random sampling in space), then our comparison layer would have had too many biases to compare to the model outputs. To address the reviewer's concerns we have now included all of the archaeological ages underpinning the comparison layer in the Supplementary Information (new Table S1).

The obvious, and recognised, issue is the site of Madjedbebe, which has a date at least 65 Ka, possibly older. Unless this site is rejected (unlikely as one of the authors excavated it), it seems to me that it constitutes one of Huxley's 'ugly facts' in the face of a beautiful model. As the difference in probability between the 50 Ka date and the earlier date entry is not that great, it would be worth paying more attention to the actual evidence, especially as it rules directly to their major claim.

RESPONSE #5: While we acknowledge the general archaeological debates over Madjedbebe — and our paper includes several authors on both sides of the issue — we must highlight that the study presents analyses both including and excluding the site for validation purposes. Hence, our paper intrinstically acknowledges the uncertainty around the site, but has found limited difference whether the site is included or not (to the main conclusions, albeit the scenario *without* Madjedbebe produced worse correspondence to the archaeological map layer). This result and approach are prominent throughout both the main text and Supplementary Information, i.e.,:

- Fig. 1c
- "When we repeated the analysis excluding the Madjedbebe site …" (main text lines 147–152)
- Figures S3 and S4
- "To that end, we derived a second archaeological layer where we excluded all of the dates[3,18-24] from the Madjedbebe site, and then redid the Signor-Lipps- and spatial bias-corrected layer for comparison (Fig. 1c). Rerunning all scenarios as for the original archaeological layer, we generated a new scenario rank (Supplementary Fig. S3 and S4)" (Supplementary text lines 63–66)

All figure references here refer to the revised order.

It is essential here to recall that excluding the Madjedbebe dates resulted in a much poorer fit overall, even though the main conclusions were upheld. What is unsurprising is that the timing component became less conclusive, with no clearly dominant entry time (55 ka and 50 ka were, as expected, slightly more supported, although 70 ka and 75 ka were a close second; Fig. S3). What is rather telling though, is that the form of the $K{\sim}P_p$ relationship and the entry sequence were identical to the results *including* Madjedbebe (Fig. S3).

2. In the same vein, another conclusion is the very rapid colonisation - 4000-6000 years in round figures. My experience in modelling population dispersals is that models tend to come

up with very rapid rates - in practice, modelled populations tend to breed like rabbits, and spread very fast.

RESPONSE #6: Models can only use the functions they are given. In our case, the rates of spread are contingent on how fast the population can grow (grounded in well-established human demography, including results from full age-structured models[25]), the rules of immigration/emigration (contingent on hindcasted carrying capacity), and dispersal rates (again, grounded in both allometry and anthropological evidence). The model cannot go beyond these rules, but it samples from within the supplied ranges according to the probability density functions applied.

So, the model predictions are as real as the plausibility of their component parameters. In terms of the rate of spread itself, we explicitly deal with the point raised by the third reviewer regarding maximum dispersal distance (see Responses #33–36).

The problem is getting the rates down to something that sort of makes common sense.

RESPONSE #7: So, following Response #6, if the underlying parameters in the model are plausible and supported ("common sense"), then so too must the emergent patterns. However, we are not in the business of stating anything as "common sense", and instead require evidence to support our conclusions. Still, the emergent rates of spread are not beyond other estimates — those returned by our model are entirely in keeping with genetic estimates of dispersal rate in ancient Sahul[15]. We have now added that additional support in the Discussion:

> "Our estimated migration rates of 0.71–0.92 km year^{-1} are entirely consistent with previous estimates of rapid spread throughout Sahul from genetic evidence[15]." (lines 342–344)

It is possible that these were the rates, and that is the period of time to saturation, but either this needs to be assessed more in the context of very rapidly expanding invasive species (which do occur), or there needs to be a bit more sensitivity testing to the relationship with carrying apacity, or the level at which carrying capacity is set.

RESPONSE #8: We disagree that "invasive species" is the appropriate model to which we should compare human dispersal, especially considering that we did not attempt to correct for any vital or dispersal rates that can be higher in populations at the leading edge of species invasions[26,27] (but see Response #38).

As explained in Response #6, the emergent dispersal pattern and rate is contingent on population growth, rules of immigration/emigration, and dispersal rates. The sensitivity analysis we provided (and have now updated — see Responses #33–36) showed clearly that the parameters related to population distance to carrying capacity for emigration (minimum and maximum N/K; see Fig. 5) had next to no effect on the rate of continental saturation. Further, the only other parameter dictating movement from a focal cell modified by carrying capacity (hydrological resistance Ω) had minimal effect on the saturation rate (Fig. 5).

But just to make this point even stronger, we re-ran 100 iterations of the top-ranked model (50 ka southern entry only) by halving *K* across all of the landscape (keeping the stochastic resampling across time and space as before). This merely increased the lower bound of the estimated time to saturation to 187 generations (156 in the original model), but hardly changed the upper bound (202 generations compared to 200 in the original model). We feel there is no need to provide this new result in the manuscript because it merely reflects what the global sensitivity analysis concludes (but we will be guided here by the editor's preference).

3. I have no particular quarrel with the southern point of entry, but Malaspinas et al, in their paper on the genomics of Australia, suggest that the Sahulian population entered as a a single clade, but diverged quickly afterwards. While that is feasible from the southern entry, the geographical patterns of relatedness between Australians and Papuans needs to be accounted for. I should add, in fairness, that the Malaspinas genomic dates accord well with the preferred 50 Ka scenario in terms on chronology, even if they do not geographically.

RESPONSE #9: While genetic evidence contradicting the Malaspinas *et al.*[14] conclusion of a single entry already existed at the time of submission[28] (which we cited in the Introduction), new evidence[16] has been published since then in further support of the dual-entry hypothesis.

In this new paper by Pedro and colleagues[16], the results strongly suggest a diversification of mitochondrial lineages in Sunda/Wallacea and a dual north/south entry into Sahul, with little gene flow between northern (today's New Guinea) and southern (today's Australia) populations afterwards.

It is important to note that this new genetics paper cannot identify routes or entry points — it only show phylogeographic structure whereby lineages from New Guinea and Australia are deeply rooted. They[16] used the same Bayesian framework as did Tobler and colleagues[15] to estimate time in the phylogenies, and ended up with the same wide confidence interval for first entry into Sahul for all mitochondrial lineages.

In conclusion, these new mitochondrial data support our models insofar as:

- the timings between genetic results and our model are compatible;
- the genetic data show dual entry, also supported by some of our models;
- based on mitochondrial data, it is impossible to determine if the northern and southern entries were simultaneous or if one preceded the other — our models disentangle this; and
- there is clearly evidence for a Late Pleistocene population expansion in the genetic data.

Perhaps the most surprising - but not impossible - claim in the paper is the proposed population of Australia at demographic equilibrium - presumably before 40 Ka, if I understand the paper correctly, but certainly in the Pleistocene. Over 6 million people is a very large estimated number. Previous estimates have varied considerably, but as far as I know none has exceeded 2 million (certainly for the Pleistocene), and most are considerably less, seldom exceeding 1 million. While there is some discussion of this in the text, one cannot help wondering if this number reflects a problem with the model.

I am a fan of modelling approaches, as I think one of the biggest problems in most archaeological work has been the absence of properly quantified expectations. I think this is a very promising approach and paper, and would like to see a version of it published. However, I think some further revisions or testing of the parameters are required to address the issues outlined above. I always think models are at their most interesting where they highlight anomalies, and these are what could very positively be assessed.

I have not checked the code or statistical work, which would require specialist assessment.

RESPONSE #10: This outcome is not the result of any problem with the model. It is a reflection of the distribution of carrying capacity estimated from the hindcasted climate model, the best-supported relationship between carrying capacity and net primary production, and the expression of catastrophic die-offs altering population trajectories.

As we discussed in the paper, we are fully aware that this is larger than previous estimates[29,30] (done mainly by co-author Alan Williams). However, as the reviewer stated, this is not impossible for several reasons:

- Previous estimates have ignored the New Guinea component of the entire Sahul population (~ 15% of Sahul's landmass area during the window of investigation). It turns out that this equates to a maximum of ~ 1 million people in New Guinea alone (compared to the remainder in 'Australia' if we split Sahul roughly across where Torres Strait is today); we have included this population split in the Abstract and Results in the revision).
- As the reviewer is no doubt aware, the reconstruction of population sizes and trends from archaeological material is contingent on many assumptions regarding discard rates, taphonomy, and sampling bias. Thus, these models have their own limitations, and as such cannot be robustly used to indicate that our model is 'wrong'.
- The population size estimates vary markedly depending on the form of the relationship between carrying capacity and net primary production. While we found the best fit for the rotated parabolic (which makes sense in terms of human[31] and other species' ecology[32] as we described), we only tested three variants of the relationship. As is shown in updated Fig. 3, the relationship is important for the final estimate of population size, because each one weights the contribution of a cell across the landscape differently (see new maps included in Fig. 3 that show example population distributions at 300 generations). Even though they provided worse fits to the archaeological data, the two other hypothesised $K \sim P_p$ relationships predicted maximum populations approximately half as large (3.1–3.5 million; Fig. 3).
- As we stated in the Discussion, these estimates reflect the *maximum potential* population sizes under the assumptions of the $K \sim P_p$ relationship, the incidence and distribution of catastrophes, and the reconstructions from other hunter-gatherer societies[33]. If other, unknown conditions prevented populations from reaching carrying capacity (warfare, difficulty in obtaining sufficient food resources, bushfire, etc.) beyond those we considered using the spatially clustered catastrophe function, then the population estimates would be smaller accordingly.
- Finally, we also stated in the Discussion that the population densities we derived over the entirety of Sahul (0.55–0.57 people km^{-2}) are not dissimilar to

previous estimates of hunter-gather densities in Sahul (0.34 ± 0.24 people km^{-2}; note that the upper limit of the previous estimate equates to 0.81 people km^{-2} assuming a Gaussian distribution, which is well above even our own upper estimates), or shifting agriculturalists in New Guinea (4.37 ± 4.22 people km^{-2}); granted, the latter estimate is contingent on the additional food supplementation derived from agriculture, so it is expected to be higher than hunter-gatherer densities.

We have now summarised these caveats in the revised Discussion.

Reviewer #3

This interesting paper attempts to assess the fit between alternative models of colonization and spread through the Sahul and the archaeological data, while considering ecological/climatic factors.
The study is an impressive exercise along two lines of exploration – (1) of utilizing an array of inference methods and bias corrections for the archaeological data, and (2) the implementation of generative models that simulate alternative colonization and spread dynamics across a realistic explicit spatial grid.
I am generally skeptical regarding the possibility to precisely infer a value for singular events in systems that involve so many moving parts (in this case: exactly the when and the where of the colonization).

RESPONSE #11: We are not attempting to argue for anything "precise" regarding this obviously complex system; rather, we are demonstrating through the use of scenario analysis which predictions have more *relative* support than others. We cannot possibly test all combinations of plausible entry points, entry times (singular or multiple) or relationships between net primary production and carrying capacity. No computer on Earth is capable of such a feat.

Instead, we are taking the most plausible ranges of previously hypothesised phenomena, and testing incrementally which have the most *relative* support as indicated by the corrected archaeological map layer(s). As we mentioned in Response #5, models (testing different scenarios) are necessary simplifications of a chaotic and complex universe that attempt to extract the most important elements driving the system toward particular outcomes or phenomena. Thus, we are testing which simplifications of reality (model scenarios) have *relatively* more support based on the available evidence.

Models that consider multiple interacting dynamics like this one are super-useful to get a handle on order-of-magnitude of timescales of processes, to demonstrate feasibility of alternative qualitative dynamics, to assess the relative importance of different parameters and processes in the larger picture, and to fit parameters of recurring processes (say, for example, the rate of migration between populations, or the rate and distribution of effect-sizes of adaptations, or even the effective size of a population bottleneck which is in reality a convolution of dozens of demographic fluctuations of different magnitudes, and so on).

However, testing with such models whether a scenario of colonization at 75 kya or one that occurred at 50kya is more likely, seems to me over-ambitious when there are so many unknowns. This study didn't convince me otherwise.

RESPONSE #12: As mentioned in more detail in Response #11, this is exactly what the models can do — assess *relative* likelihood. They do not, under any circumstance, recreate reality in its entirety. In fact, it is remarkable that, based on stochastically resampled principles of human ecology, we can find any congruence at all to the patchy archaeological data that do exist. In our opinion, this is one of the most remarkable outcomes of the approach, and demonstrates that our models do capture a major component of the dominant mechanisms driving human migration and settlement in ancient Sahul.

It is a bit odd to test only 5 or 7 scenarios (time-wise), get nearly the highest-possible support for the latest date tested, and then have the title use the wording "models reveal rapid early peopling…" in the title.

RESPONSE #13: Given that we know that arrival was at least by 50 ka, and plausibly much earlier (> 75–80 ka), we chose the most realistic range based on the available evidence to test. Had we opted for more than a 5000-year increment between these limits (say, every 100 or 500 years), the degree of implied precision would have gone far beyond what we could conceivably expect our model, and the available archaeological evidence, to reciprocate. Thus, we chose these eight different periods to examine the *relative* evidence. We did not in any way imply that 50 ka (or 75 ka) were the *precise* dates of major entry; rather, we show that these general periods were more supported than the others within that plausible range.

But, in an effort to be entirely transparent, we have added another four scenarios for the purposes of the response (for the logic provided above that initial arrival was most certainly ≥ 50 ka) — these included entry only in the south at 45 ka or 40 ka (i.e., two scenarios), and entry in the south initially at 45 ka or 40 ka, followed by a second entry via the northern route 2000 years later in each case (i.e., another two scenarios). You can view these new relative correlations (boldface) to the archaeological layer (including Madjedbebe) below (all scenarios here assume the rotated parabolic $K\sim P_p$ relationship):

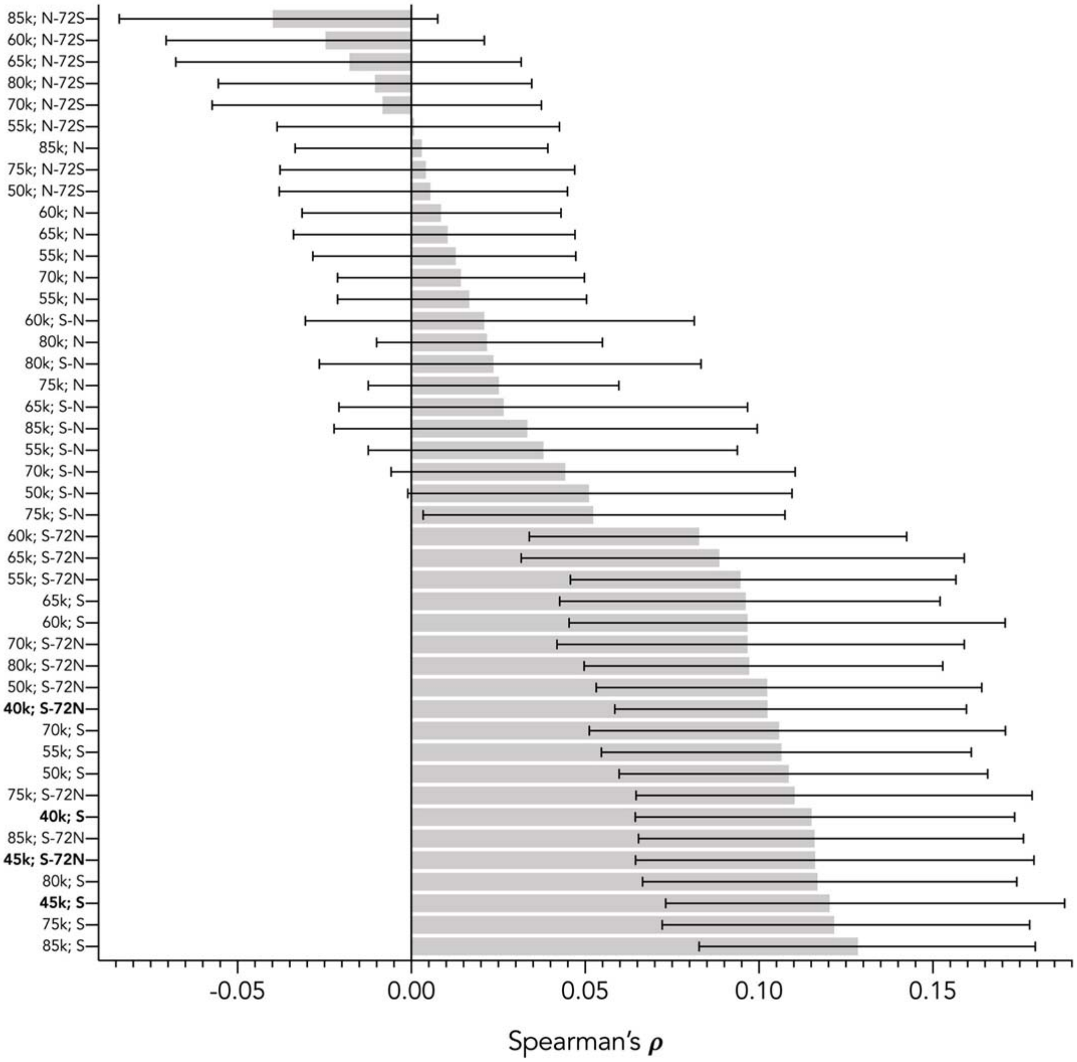

These results clearly show that the new scenarios are inferior to the top-ranked scenarios within the range we originally presented. Again, we have not run these for all $K \sim P_p$ relationships, so we do not consider it essential to include these results in the manuscript itself.

The same logic applies to the five different entry sequences we tested, based on the two known (and most plausible) *major* entry routes; we cannot consider other minor routes without completely redesigning both the objectives and structure of the model.

For the three $K \sim P_p$ relationships, we have no available data to design a more specific set of scenarios (apart from a general supposition that it more likely to be non-linear[31,32,34]), so we proposed three rather different scenarios to demonstrate which extreme version(s) had more *relative* support.

All told, this means we tested **8**×**5**×**3** = **120** different scenarios for each of the two archaeological comparison layers (with and without Madjedbebe). With **240** scenarios tested (and many other set-up scenarios we do not report here), each requiring > 30 hours to run on the supercomputer cluster, this means that we logged well over **7200 hours** (**300 days**) of computational time to provide just these results (this does not

include the many weeks necessary to run the sensitivity analyses). In our view, this is a major undertaking to test the most plausible range of values.

It is also our opinion that the word 'reveal' is therefore appropriate for the title to get to the heart of our main results succinctly. That said, we would be happy to entertain other suggested titles by the editor or the reviewers.

Population size estimates are also odd: they are "baked" into the model to a great extent by the parameters of carrying capacity; they don't rely on any archaeological data (I think), and they aren't tested in comparison to archaeological data. Thus, the result that the model predicts larger population sizes than previously considered is primarily a model assumption, and not a result, isn't it?

RESPONSE #14: It is possibly unjust to claim that anything in a temporally and spatially dynamic model where all parameters are stochastically resampled is "baked". This is not how such models function. There is nothing deterministic about this model, so one could never predict an outcome based on a single (e.g., median) value for any particular parameter.

For example, each cell has a hindcasted net primary production confidence range that changes through time, from which each iteration samples randomly. Nothing is constant over space or time here. When translated to carrying capacities, these sampled ranges are carried over into expressions of maximum human densities. Then, the demographic and dispersal components are also derived from resampling probability distributions. Therefore, not a single component of this model can be predicted based on first principles — the emergent property of all these stochastic processes is exactly why such a model is needed to project any element of interest (spread rate, densities, etc.). *No other study has ever come close to this degree of sophistication for modelling ancient human spread across any landscape.*

Furthermore, uncertainty is further derived from the carrying capacity ~ net primary production relationships (largely unknown, but see Response #13), and this is an entirely novel addition to the model.

Finally, the model does not, nor should it, be predicated on any archaeological data because the latter are used as a form of validation to rank scenario results. Had we included archaeological reconstructions within the model structure itself, it would have been circular to test them against the model outcomes.

The reason that previous studies considered smaller population sizes is that most studies do not assume that paleolithic carrying capacities align very well (perhaps due to cultural complexity, or maybe other factors) with present-day hunter-gatherer societies. So the difference between this study and others seems to stem from the assumptions, not the results (I'm agnostic regarding which is correct; just pointing this out).

RESPONSE #15: The reviewer's agnosticism nothwithstanding, we are not aware of any evidence purporting a measured difference between Palaeolithic and present-day hunter-gatherer carrying capacities. This is an assumption based on the notion that

cultural complexity increases monotonically through time. There is certainly no evidence for this in Pleistocene Sahul (although there is some evidence perhaps for technological development in the mid-Holocene period). Without evidence to the contrary, we are compelled to work with the available data.

The assumptions regarding mortality/reproduction rates due to minimum viable population size seem to me unwarranted. A population size suffers from inbreeding below a certain effective population size in the long run, after equilibrating to the genetic diversity that is characteristic of such a small populations size. A population of 100 individuals that is sampled at random from a 10,000-individual population size will have heterozygosity and even allelic diversity that is only slightly skewed compared to the original population size, and very very far from the genetic diversity that characterizes an N=100 population. It would take dozens or hundreds of generations (on the order of the whole time of the simulation) for heterozygosity to drop to the low levels that characterize N=100 at equilibrium and for inbreeding depression to kick in.

RESPONSE #16: The reviewer is incorrect here, and the lead author has published extensively on this topic with one of the world's leading conservation geneticists, Professor Richard Frankham. Our 2014 paper[35] published in *Biological Conservation* (now with 463 citations in Google Scholar, which we cite in this manuscript) explicitly shows that inbreeding depression below population sizes of 100 can manifest in as few as five generations. The problem with how the reviewer states this issue is that we are not modelling 100 individuals sampled at random from a large, outbred population; rather, we are referring to the founder effects of small groups dispersing from sometimes already-small populations at the edges of distributions and in areas of low carrying capacity.

Further, the assumption that the source population breeds randomly and is outbred is decidedly incorrect for almost all species studied, including humans. In fact, the ratio between effective population size and census population size averages around 0.1, meaning that the degree of expected heterozygosity is much lower (~ 90%) in real populations than random mating would otherwise predict[36]. We demonstrated this phenomenon well in our 2019 paper examining minimum viable populations for the first humans in Sahul[25].

In the case of humans in particular, we are also assuming that many other types of Allee effects (not just inbreeding depression) operate at low population sizes below the minimum viable one, including social disruption, inadequate social structure to fulfil all roles, lack of dispersed child raising, etc.

We have elaborated on this issue further in the relevant section of the Methods to make these elements clearer.

> "This assumes that individual fitness declines when populations decline below the minimum size due to inbreeding depression from non-random mating and other Allee effects related to social disruption."

There is no statistical significance on anything. This is fine, but there also isn't an alternative statistical approach that would help tell apart what differences are more or less reliable (e.g. some kind of Bayesian analysis, or other non-frequentist approach). It seems from the error bars in figure S2 that more than half of the scenarios tested have fit quality that are not different from one another in a statistically significant way.

RESPONSE #17: We acknowledge that we did not provide adequate detail here, which might be the cause for some of the reviewer's confusion. To describe what we did, it is important to note that a rank correlation (non-distributional) such as Spearman's over a landscape composed of nearly 4000 cells will nearly *always* provide a small Type I error estimate (probability that the estimated coefficient could be generated at random). Without delving into the meaninglessness of arbitrary rejection thresholds and the (ir)relevance of 'significance'[37], this means that we had to avoid pseudo-replication and provide a way to test whether the estimated Spearman's ρ were $\neq 0$.

To do this, we randomly resampled cells from the landscape for both the estimated arrival times from the modelled scenarios and the validation layer, and determined the ρ for these cells. This also partially avoids spatial autocorrelation by reducing the number of adjacent cells in the sample. We then calculated the upper and lower 95% confidence limits for ρ for each of the 100 iterations of the validation run for each scenario tested. We then resampled those ρ across all iterations to provide the confidence intervals reported in Fig. S1 and S4. Where the confidence intervals do not overlap 0, the values are statistically non-random (i.e., 'significant').

We have now added text describing this approach in a new section of the Supplementary Information (second section following the description of the reference archaeological date compilation), and referred to this new section in the main text:

> "***Spatial correlation between modelled and archaeologically derived dates of initial arrival***
> For each scenario, we tested the correlation between the modelled date of initial arrival for each cell in the landscape and the date derived from the bias-corrected archaeological layer. To do this, we randomly resampled cells from the landscape for both the estimated arrival times from the modelled scenarios and the validation layer, and determined the Spearman's ρ for these cells. This approach partially avoids spatial autocorrelation by reducing the number of adjacent cells in the sample. We then calculated the upper and lower 95% confidence limits for ρ for each of the 100 iterations of the validation run for each scenario tested. We then resampled those ρ across all iterations to provide the correlation coefficients' confidence intervals reported in Fig. S1 and S4. Where the confidence intervals do not overlap 0, the values are statistically non-random."

I suddenly realized – the best fits of the model and the data have a rho on the order of 0.15? That doesn't sound very high. Maybe I misunderstand. Perhaps add an explanation.

RESPONSE #18: On the contrary, these values are remarkably high given the uncertainty in both modelled outputs and the bias-corrected archaeological layer. This

is why we avoided the term 'validation' because a validation assumes that the base to which one compares a modelled output is of high accuracy and precision. This is therefore more of a test of congruency. As mentioned in Response #17, many of the scenarios demonstrate non-random association with the archaeological map layer (Fig. S1, in particular).

It is extremely annoying to read the results with the figures not embedded in the appropriate place in the text. Nat Comms allows submission with embedded figures and captions. An annoyed reviewer is a less constructive one.

RESPONSE #19: Embedding figures is not required for submission, but we have complied with the reviewer's request in the revision.

Figure S1, which is crucially important for understanding what the main findings are based on, is in the supplementary, and very hard to understand. A legend would help a lot, and clearer explanation and marking of what each isohyet means would help. Are they currently at numbers like "24,380 kya" instead of round numbers? When the error bars around each estimate are on the order of magnitude of hundreds or thousands of years? Since you have a full topographic map of the whole "landscape of timing", you can choose which isohyets to mark…

RESPONSE #20: See Response #1 (now provided as the first main figure).

Also, are they estimated using dating methods as an absolute date, and then translated to a date relative to an inferred date of first arrival, which is the greatest unknown in the study? Or did I misunderstand? Either way – probably deserves some clarification.

RESPONSE #21: We neglected to specify that we calibrated all radiocarbon dates to calendar years before present (defined as AD 1950 by convention) using the Southern Hemisphere Calibration curve (ShCal13) from the OxCal radiocarbon calibration tool version 4.2 (c14.arch.ox.ac.uk)[38,39]. We then did all Signor-Lipps manipulations on these calibrated dates (and incorporated their ensuing errors within our bias-correction procedure as described) as indicated in the Methods. We have now included this information in the first section of the Supplementary Information.

This is a bit of a blasphemous question, but – how much does the modeling exercise add to a direct estimate of time-of-arrival and of rate-of-spread that are assessed directly from the archaeological data? That is, if you just take the signor-Lipps-spatial-biased-corrected map, what is the earliest date that appears there, and does it point to the same primary introduction point that the study currently highlight? And is the second-earliest date at 50 kya, near the northern inferred introduction point? In other words, is the most common-sense timing+route of introduction, based on the corrected map, the same timing+route that the complicated simulations lead to? If yes, perhaps clarify this, and also discuss what added value the simulations have? (they definitely have a cost in the form of relying on 10-20 assumptions that are all questionable).

RESPONSE #22: This is exactly what the correlations do (indicate which scenarios have the greatest correlation to the archaeological inference). However, there is an impediment to doing what the reviewer suggests, in that the spatial sparsity of the archaeological data means that the inferred introduction timings after the bias corrections are too early to be taken as reasonable. We therefore only expressed the maps in terms of relative appearance times (with intial entry set to time 0). This way, we avoided the large uncertainty associated with the oldest ages and in regions with the fewest dates (e.g., New Guinea in particular).

While we did provide the bias-corrected archaeological maps for scrutiny, it is essential to note that these are entirely phenomenological and provide no mechanisms for how people spread across and settled the landscape. Thus, the archaeological layer alone provides no information whatsoever about the *processes* of peopling, which is what our mechanistic model does. We keep coming back to this point, but the fact that we can yield the main processes leading to the phenomena observed from the data we have at hand reveals the important elements of human ecology that archaeology alone cannot.

I suddenly realized that you estimate that the colonization of the whole continent happened within 5000 years, which would be 20,000 years before the second arrival that you suggest is likely at the north site, if the first colonization was in the western site at 75,000. That doesn't seem to make sense.

RESPONSE #23: We are not entirely clear about what the reviewer is suggesting, but it appears that there is some confusion. Among the double-entry scenarios we tested were a 75 ka entry in the north (or south) followed by a 73 ka entry in the south (or north), or a 50 ka entry in the north (or south) followed by a 48 ka entry in the south (or north). Other scenarios had simultaneous entry at the allotted times in both the north and the south. We did not code any scenarios where there was a 20,000-year lag between entries.

Which leads to the question: did you penalize the model for added complexity? A model that allows more than one entry point would necessarily be able to produce a better fit, as it has an added level of freedom. If you did, how? If not, why? Either way – worth discussion.

RESPONSE #24: This is a cellular-automaton model where the emergent properties of interest (rate, pattern of spread, population size) result for the interactions of all parameters. There is no distribution to which the output is fit, there are no degrees of freedom, and therefore 'overfitting' is a non-issue. The concern is whether the output can emulate something realistic, which we determine based on the correlation with the derived archaeological map layer. We do not think this requires additional discussion because it is a well-understood statistical perspective. We now include these concepts are in the new text in the Supplementary Information (see Response #17).

In addition, the results show clearly that adding a second entry *did not* improve the fit (i.e., a single, southern entry had the best fit to the archaeological data), so this disproves the assertion above that more entry points would be necessarily better.

The model prediction that the arid region would have been a barrier for movement is, again, not a result – it's an assumption, baked into the model through the carrying capacity calculation.

> **RESPONSE #25**: Again, there is nothing "baked" into the model; rather, we are imposing empirical relationships between net primary production and carrying capacity. That the arid arrays often do not accumulate sufficient population sizes to be deemed as 'settled' is an emergent property of low carrying capacities, dispersal modifiers with aridity, intrinsic population growth rates, and likelihood of rescue immigration. So, we did not specifically include arid zones in the model as any form of barrier or deliberate design; the modelled outcome is purely a result of underlying data, and the *stochastic* model. A 2016 *Nature* paper[40] followed similar logic.

Modeling dispersal – not sure how exactly you implemented dispersal, but simplistically you could imagine that a group decides to bud off, and that this decision is not effected by the availability of nearby unsettled territory or nearby water resources, and only secondarily the group decides where to go while taking into account these considerations, or alternatively you could have the availability of vacant and close-to-water routes/land be baked into the calculation of whether to immigrate at all. The choice among these (and similar considerations) could lead to huge differences in the emergent dynamics.

> **RESPONSE #26**: We agree that non-ecological (environmental) decisions to emigrate could modify or run counter to many of the ecological constraints. We have made this clear throughout the manuscript — that it is primarily an environmentally driven model. That said, we cannot possibly model the vagaries of cultural, societal, and emotional human decision-making in a model testing the ecological mechanisms underlying human dispersal, especially for societies that existed over 50,000 years ago. If we had data to the contrary, it might be possible, but without which it becomes a 'what if' scenario with no clear underlying hypotheses. Perhaps more importantly, the spatial scale at which the model operates (55 × 55 km) most likely reveals the general environmental constraints and drivers, below which cultural decisions probably operated.

The same is true regarding distance of dispersal – you can view it as a pre-determined coin-toss, or you can imagine that a group continues on and on until they find an appropriate place to stay for the longer run (the "on and on" can play out within years or across a few generations). The latter option might lead to non-intuitive results, e.g. that deserts are actually crossed much faster than expected, and that spread through zones of intermediate bio-productivity might be way faster than zones with high productivity (even under the linear K-biodiversity relation curve).

> **RESPONSE #27**: We have already accounted for this in two ways: (*i*) by modifying the dispersal probability function according to aridity (net primary production), such that drier areas have a tendency to produce more long-distance dispersal events, but (*ii*) long-distance dispersal in the most arid regions is ultimately constrained by the availability of water (absence of water limits dispersal). Thus, we have a probability distribution of dispersal pushed and pulled by these opposing functions.

I think I wrote this, but modeling dispersal distances and rates of an "invading" species that is expanding its range into unoccupied territory (unoccupied by another similar organism, that is), based on data estimates for populations of animals and hunter-gatherers that are at a steady-state might be extremely misleading, possibly hitting off-target by orders of magnitude. At least discuss this.

RESPONSE #28: There is no steady state in the model because carrying capacities and movement parameters are constantly shifting temporally and spatially, and are resampled within the hindcasted range (carrying capacity) or probability distributions (movements). We have dealt with any concerns regarding movement rates in detail in Responses #34–36.

That said, perhaps the reviewer is also referring to the idea of altered carrying capacity from occupancy and resource depletion. Of course, this is already accounted for by the very concept of carrying capacity included in the model because it imposes a limit on how much the local landscape can provide resources for a given population. This notion is validated in the vast, global database and analysis provided by Tallavaara and colleagues[33], which quantifies the spatial variation in densities relative to net primary production.

So, the only other forms of carrying-capacity modification would be technical (i.e., increasing local productivity through agriculture — something we specifically avoided for Late Pleistocene Sahul because of a complete lack of evidence [there is increasing evidence this might have occurred in some regions during the Holocene]), or some form of environmental hysteresis. The latter is the subject of much debate, and so there are no clear mechanisms we could conceivably include in the model. One scenario is that the loss of megafauna via human-caused overexploitation changed the structure of both the vegetation and animal communities, leading to a new equilibrium and therefore different carrying capacities to those that could be predicted using hindcasted net primary productivity. We hesitate to add speculation regarding this (currently untestable) hypothesis in the context of human settlement mechanisms and patterns.

Global scale conclusions – no support for coastal-migration preference: of course the model here didn't support such preference; the assumptions that the idea's proponents highlight that would make such a scenario feasible are not considered in the model!
(to name some - consistent supply of same-type resources that are readily accessible along the coast and don't require adaptation to new methods of hunting/gathering/handling, ease of movement along the coast – by foot along the coastal plane and/or by means of water-transportation).

RESPONSE #29: Again, the reviewer is referring to putative cultural decision-making that is well beyond the scope of our model (or its capacity, given the lack of data), which, we emphasise, is attempting to reveal the main ecological mechanisms influencing human settlement patterns. See also Response #26.

However, many of the coastal margins were indeed more productive and therefore attractive to humans, and our hindcasted primary production-derived carrying capacities would have revealed this. This also depends, of course, on the relationship between carrying capacity and primary production (Fig. S2). More importantly, our model does not downgrade the importance of coastal movements *per se*; rather, it reveals that many interior regions were ecologically plausible areas of both dispersal and settlement. This is a novel finding that in no way negates the discussion surrounding coastal phenomena.

We further direct the reviewer to a 2018 paper[41] by co-authors Alan Williams and Sean Ulm on sea-level change in the Late Pleistocene, which demonstrates that the coastal fringe was highly active (in terms of sea-level changes) during this period, and might not have been conducive to large-scale use by early Aboriginal populations.

Calculating a minimal global arc and fitting its length to the mean rate of per-generation movement that the model suggests seems extremely simplistic and misleading. At the least, it almost sounds like it implies that humans knew how to take the shortest route to the Sahul, and that that's where they were going. The hyper-sensitivity of this argument to deviations seems obvious (e.g. what if the mean rate of movement is twice or three times faster? Or twice slower? What are the confidence interval with which you estimate the 0.71-0.92 km/year rate, if you consider slight alterations to the more arbitrary among the model assumptions?)

RESPONSE #30: We did not suggest that people were necessarily moving in the most efficient manner; rather, we demonstrated that the time taken to traverse the continent via complete saturation (i.e., all cells occupied/settled) could have occurred as quickly as 0.71–0.92 km $year^{-1}$. Because of the cell-based diffusion pattern, we are not modelling specific routes *per se* (we are modelling settlement), so we are obliged to provide a distance that has some relevance to the scale of the study (i.e., the distance between entry points and the southeastern limit of Sahul). Furthermore, many least-cost path models used to examine the patterns of movement by ancient peoples implicitly assume that movements are 'efficient'. From a community perspective, the more people are committed to solve a problem (e.g., finding the best route), the more likely the group will, on average, choose this path. We have already demonstrated previously[5] that people arriving in Sahul was not accidental, so community strategies likely existed to promote efficiency.

Also, the reviewer did not have the benefit of viewing animations of the model unless she/he reran the R code we provided. We have now uploaded, with the revision, a small animated .gif (file: Sahul spread animation example.gif) of the 50 ka southern-entry simulation (single iteration) to show that the diagonal northwest–southeast distance to estimate rate of spread is not unrealistic given the behaviour of the advancing wave across the continent.

The range of 0.71–0.92 km $year^{-1}$ comes from the 100 iterations of the most-supported scenarios, because stochastic models will give slightly different outcomes for every iteration run.

We deal with the comments regarding maximum dispersal distance in greater detail in Responses #34–36, but the model outputs of spread rate are relatively invariant to large changes in maximum dispersal.

I don't think the model adds to the current knowledge about "adaptability and technologically advanced state of anatomically modern humans" – that isn't what the model models, and any results along these lines are direct reflection of the assumptions, not an emergent result. Other papers (by some of the same authors) do perhaps support some of these statements, but this model doesn't lend any additional support, as far as I can tell.

RESPONSE #31: We are reluctant to remove this section, given that we can infer from the pace of people moving across a wide range of environments that they must have been adaptable and advanced. However, should the editor deem it necessary, we can remove it.

The same is true regarding the population sizes, as noted earlier. The finding that might support the idea that these weren't small unorganized bands but a larger and more advanced population have to do with the number of individuals that arrived at the same time (nearly) and things like that – but these are results of some of the authors' previous studies, and this one relies on them – it doesn't add any independent support as far as I can tell.
• (I'm agnostic regarding whether these claims are correct; I find them very reasonable, I like to think they are true, and I find some previous papers from these authors about these questions convincing – I'm merely pointing out that these aren't results of the model in this study, as they are presented)

RESPONSE #32: Again, we are reluctant to remove this clause for the reasons provided in Response #31, but we will take the editor's advice here.

Model sensitivity section: I don't think this is what I'd consider a sensitivity analysis.

RESPONSE #33: The reviewer is incorrect; what we present is exactly a sensitivity analysis. (Indeed, the lead author has previously co-authored one of the only major papers published on how to do sensitivity analyses for stochastic models of this nature, which we cite in the text[42].)

Perhaps the reviewer is thinking more of a perturbation analysis, where a single parameter is modified at a time to examine its effect on a model outcome. However, single-parameter perturbation analyses are inappropriate for stochastic models where many models interact in unpredictable ways. Only *global* sensitivity analyses (modifying many parameters simultaneously) can provide robust sensitivity measures in the presence of non-linear responses and interactions among parameters[43,44].

This is an important assessment of the importance of the different processes and parameters, but it doesn't explore how sensitive the results are (or, at least it doesn't report to the reader) to changes to these parameters.

RESPONSE #34: The results of the global sensitivity analysis revealed that only variation in the lower bound of the maximum dispersal distance (lower D_m) and the proportion of people migrating per migration event (P_{mig}) gave rise to any appreciable variation in the output of continental saturation time, with minor contributions from mean catastrophe mortality (M_{cat}) and the additional mortality below minimum viable population size (M_{MVP}) (former Fig. 4; now Fig. 5). We acknowledge, however, that the degree to which these influenced saturation time was not readily apparent.

We have therefore decided to do another global sensitivity analysis (replacing the previous one) where we substantially modified the approach in the following ways:

- We standardised the assessed variation in the variables examined to ± 50% of the median values set in the model; the submitted version of the sensitivity analysis somewhat arbitrarily set the variation in these parameters to limits ranging from -50% to +100% of the median values. In this way, we 'standardised' the assessed variation as a proportion of the median value for each parameter. We have replaced the relevant sensitivity analysis R code on Github.
- This point deals directly with the following comment (see Response #35), but requires explanation here. Upon inspection of the upper limit of maximum dispersal and how it is invoked in the stochastic simulations, we realised that we were perhaps de-emphasising this parameter's role because of a limitation on the maximum number of cells that could be moved in a single, long-distance dispersal event (irrespective of the specific probability). We have therefore taken the reviewer's suggestion and increased this cell-based maximum dispersal distance by five times, and replaced the variation in the upper maximum dispersal distance (upper D_m) in the global sensitivity analysis with this new parameter (max D_{cell}) ranging from 1× to 5× the value set in the original model (of course, the variation in this parameter is not ± 50% as for the others). This modifies the estimated probability of dispersal curve (see Fig. S7a) to the expanded version shown to the left.

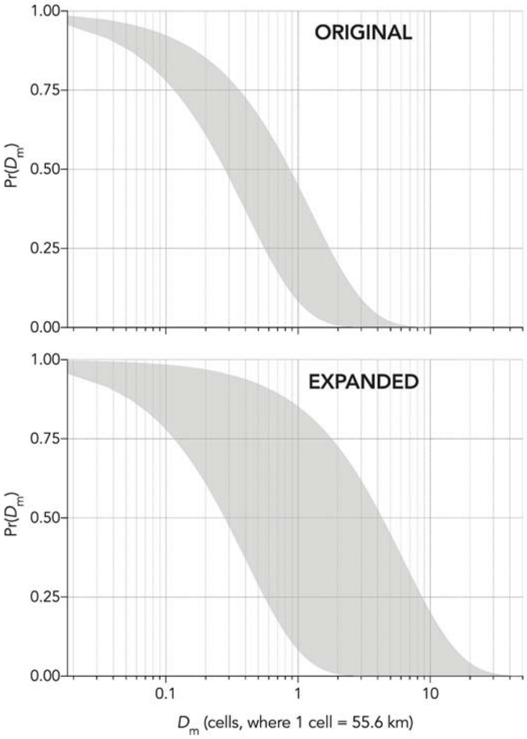

  The updated curve in the bottom panel ('EXPANDED') demonstrates how there is now a higher probability of longer-distance dispersal events across the range compared to the original curve ('ORIGINAL'); the upper limit is now 50 cells (albeit still at the lower end of the probability distribution because long-distance dispersal events are comparatively rare). We have not included the figure shown in this response in the Supplementary Information, but can do so if deemed necessary.
- Next — and this lies at the heart of the reviewer's comment — we now provide the predicted values for the saturation time (in generations) as a function of the ±

50% variation (and 1× to 5× for max D_{cell}) in the most influential parameters derived from the boosted regression tree emulator.

The new global sensitivity analysis with max D_{cell} instead of upper D_m indicates a slightly different result from that obtained previously. The lower D_m is no longer the most influential parameter (it was taking most of the influence away from the other extreme of the distribution given the limitation we imposed in the original model); instead, P_{mig} is now the most influential parameter (previously, it was the second-most influential), followed by M_{cat} (previously the third-most influential; new Fig. 5). N_{MVP} moves from fifth to fourth place. The new, third-most influential parameter is now max D_{cell}, and lower D_m is demoted to second-last in the ranking (Fig. 5). In conclusion, while variation in maximum dispersal distance now has a more realistic influence on the time to saturation, its overall effect is small.

Given that variation in only two variables (P_{mig}, M_{cat}) has most influence on the variation in saturation time, with three others providing weak (but detectable) variation (max D_{cell}, N_{MVP}, M_{MVP}), we only show these five relationships in a new 5-panel plot in the Supplementary Information (new Fig. S5). In other words, the relationships are all more or less straight lines for the remaining, non-influential parameters. We also provide the relative percentage change in saturation time with the modelled variation in each of these six parameters in new Fig. S5.

Sensitivity analysis should answer the question: what changes, in which parameters, would lead to a qualitatively different main result/s. In this case, I'd like a sensitivity analysis to include, for example, an exploration of how much would the estimate of colonization timing and length change if the long-distance-migration distance were, say, 5 times larger than the one considered (a scale of long-distance dispersal that is clearly within a reasonable range, given the references mentioned for human yearly mobility info). (this is just an example, of course. The same is true for each of the parameters, particularly those that are highlighted as most influential).

RESPONSE #35: While the original global sensitivity analysis identified only the *lower* limit of the maximum dispersal distance as having an impact on time to saturation (with negligible effect of the *upper* limit of the maximum dispersal distance; former Fig. 4), we have now — as described in detail in Response #34 — completely redone the sensitivity analysis. Maximum dispersal distance has only a small effect (albeit slightly more than we had estimated originally; new Fig. 5 and S5).

However, to emphasise this for the reviewer more clearly, we repeated a model run (100 iterations) where we invoked the new dispersal curve (bottom panel of figure shown in Response #35) for one of the top-ranked scenarios (southern entry only at 50 ka). Using the 5× max D_{cell} (i.e., 5× the value set in the original model), the time to continental saturation now ranges from 153 to 185 generations (4285–5152 years), which is only slightly faster than the range predicted by the original settings of the model (156–200 generations). This emphasises that maximum dispersal probability does not fundamentally change the model's performance or predictions.

We now provide this new result in the *Global sensitivity analysis* section of the Results in the main text.

Moreover, a sensitivity analysis might also be expected to explore breaking of the slightly-more-fundamental model assumptions. For example, the maximal migration distance in the model is 10 cells (55km each). If I understand correctly, this is undertaken maximum once per generation in the simulation.

RESPONSE #36: This is correct, but the dispersal kernel follows a probablility distribution and a stochastic resampling. As mentioned in Responses #34 and #35, we have provided another model run to demonstrate the model's relative invariance to assumptions regarding maximum dispersal distance.

However the citation from Gould suggests that this scale of movement might occur within a single bout, leaving open the possibility that some nomadic hunter-gatherers might have done this on a much more regular basis than once per generation. The wave of colonization isn't determined by the mean of the population, it is determined by the front – those few outliers, possibly even an "outlier culture" that moves fastest.

RESPONSE #37: Although this point is now moot, considering the negligible effect of increasing the upper limit of the maximum dispersal distance on the time to saturation, it is important to emphasise that we are not modelling *movement per se*; rather, we are modelling *settlement*. Thus, occasional forays *not* accompanied by establishment (and persistence) according to the minimum-group components we implemented are not taken as 'settlement' by the model. In this way, we explicitly focus on the most important pathways for settlement and determine the dominant patterns, rather than those that could be susceptible to outlier pathways of movement only. We have modified the text in the document accordingly to avoid any confusion.

Cane toads in Australia – not a very rapidly moving organism compared to some mammals – seem to be spreading PER YEAR at a rate that is very close to the far-right edge of the considered per-generation distribution; perhaps dispersal/long-distance-migration in an ecosystem with available niches is much much faster than in saturated ecosystems, from which the parameter estimates used in this study were derived?

RESPONSE #38: We do not think that cane toads are a good model for human expansion, not just because of the completely different life cycles, traits, rates of movement, generation times, and habitat requirements, but also because amphibians do not have the same complex settlement relationships as anatomically modern human societies.

However, we explicitly consider the saturation components of migration with relation to (*i*) the realised expression of maximum carrying capacity of the origin cells (i.e., how far a cell's population is away from its carrying capacity), and (*ii*) the relative saturation of the destination cells. This is a fundamental component of the stochastic model that provides the unpredictable, emergent property of expansion in each stochastic run.

References

1 O'Connell, J. F. & Allen, J. The Restaurant at the End of the Universe: modelling the colonisation of Sahul. *Aust. Archaeol.* **74**, 5-17, doi:10.1080/03122417.2012.11681932 (2012).

2 O'Connell, J. F. & Allen, J. The process, biotic impact, and global implications of the human colonization of Sahul about 47,000 years ago. *J. Archaeol. Sci.* **56**, 73-84, doi:10.1016/j.jas.2015.02.020 (2015).

3 O'Connell, J. F. *et al.* When did *Homo sapiens* first reach Southeast Asia and Sahul? *Proc. Natl. Acad. Sci. U.S.A.* **115**, 8482, doi:10.1073/pnas.1808385115 (2018).

4 Allen, J. & O'Connell, J. Both half right: updating the evidence for dating first human arrivals in Sahul. *Aust. Archaeol.* **79**, 86-108, doi:10.1080/03122417.2014.11682025 (2014).

5 Bird, M. I. *et al.* Early human settlement of Sahul was not an accident. *Sci. Rep.* **9**, 8220, doi:10.1038/s41598-019-42946-9 (2019).

6 Bird, M. I. *et al.* Palaeogeography and voyage modeling indicates early human colonization of Australia was likely from Timor-Roti. *Quat. Sci. Rev.* **191**, 431-439, doi:10.1016/j.quascirev.2018.04.027 (2018).

7 Kealy, S., Louys, J. & O'Connor, S. Reconstructing palaeogeography and inter-island visibility in the Wallacean Archipelago during the likely period of Sahul colonization, 65–45 000 years ago. *Archaeol. Prospect.* **24**, 259-272, doi:10.1002/arp.1570 (2017).

8 Kealy, S., Louys, J. & O'Connor, S. Least-cost pathway models indicate northern human dispersal from Sunda to Sahul. *J. Hum. Evol.* **125**, 59-70, doi:10.1016/j.jhevol.2018.10.003 (2018).

9 Norman, K. *et al.* An early colonisation pathway into northwest Australia 70-60,000 years ago. *Quat. Sci. Rev.* **180**, 229-239, doi:10.1016/j.quascirev.2017.11.023 (2018).

10 Veth, P. Islands in the interior: a model for the colonization of Australia's arid zone. *Archaeol. Oceania* **24**, 81-92, doi:10.1002/j.1834-4453.1989.tb00217.x (1989).

11 Birdsell, J. B. Some population problems involving Pleistocene man. *Cold Spring Harbor Symp. Quant. Biol.* **22**, 47-69 (1957).

12 Bowdler, S. in *Sunda and Sahul: Prehistoric Studies in Southeast Asia Melanesia and Australia* (eds J. Allen, J. Golson, & R. Jones) 205-246 (Academic Publishing, 1977).

13 Tindale, N. B. in *Ecological Biogeography of Australia* (ed A. Keast) 1761-1796 (Dr W Junk Publishers, 1981).

14 Malaspinas, A.-S. *et al.* A genomic history of Aboriginal Australia. *Nature* **538**, 207-214, doi:10.1038/nature18299 (2016).

15 Tobler, R. *et al.* Aboriginal mitogenomes reveal 50,000 years of regionalism in Australia. *Nature* **544**, 180-184, doi:10.1038/nature21416 (2017).

16 Pedro, N. *et al.* Papuan mitochondrial genomes and the settlement of Sahul. *J. Hum. Genet.*, doi:10.1038/s10038-020-0781-3 (2020).

17 Bird, M. I., O'Grady, D. & Ulm, S. Humans, water, and the colonization of Australia. *Proc. Natl. Acad. Sci. U.S.A.* **113**, 11477-11482 (2016).

18 Clarkson, C. *et al.* Human occupation of northern Australia by 65,000 years ago. *Nature* **547**, 306-310, doi:10.1038/nature22968 (2017).

19 Clarkson, C. *et al.* Reply to comments on Clarkson et al. (2017) 'Human occupation of northern Australia by 65,000 years ago'. *Aust. Archaeol.* **84**, 84-89, doi:10.1080/03122417.2018.1462884 (2018).

20 Veth, P. Breaking through the radiocarbon barrier: Madjedbebe and the new chronology for Aboriginal occupation of Australia. *Aust. Archaeol.* **83**, 165-167, doi:10.1080/03122417.2017.1408543 (2017).

21 Bowdler, S. 'Human occupation of northern Australia by 65,000 years ago' (Clarkson et al. 2017): a discussion. *Aust. Archaeol.* **83**, 162-163, doi:10.1080/03122417.2017.1408198 (2017).

22 Hiscock, P. Discovery curves, colonisation and Madjedbebe. *Aust. Archaeol.* **83**, 168-171, doi:10.1080/03122417.2017.1408544 (2017).

23 Wood, R. Comments on the chronology of Madjedbebe. *Aust. Archaeol.* **83**, 172-174, doi:10.1080/03122417.2017.1408545 (2017).

24 Dortch, J. & Malaspinas, A.-S. Madjedbebe and genomic histories of Aboriginal Australia. *Aust. Archaeol.* **83**, 174-177, doi:10.1080/03122417.2017.1408546 (2017).

25 Bradshaw, C. J. A. *et al.* Minimum founding populations for the first peopling of Sahul. *Nat. Ecol. Evol.* **3**, 1057-1063, doi:10.1038/s41559-019-0902-6 (2019).

26 Lindström, T., Brown, G. P., Sisson, S. A., Phillips, B. L. & Shine, R. Rapid shifts in dispersal behavior on an expanding range edge. *Proc. Natl. Acad. Sci. U.S.A.* **110**, 13452, doi:10.1073/pnas.1303157110 (2013).

27 Chuang, A. & Peterson, C. R. Expanding population edges: theories, traits, and trade-offs. *Glob Change Biol* **22**, 494-512, doi:10.1111/gcb.13107 (2016).

28 Nagle, N. *et al.* Mitochondrial DNA diversity of present-day Aboriginal Australians and implications for human evolution in Oceania. *J. Hum. Genet.* **62**, 343-353, doi:10.1038/jhg.2016.147 (2017).
29 Williams, A. N., Ulm, S., Cook, A. R., Langley, M. C. & Collard, M. Human refugia in Australia during the Last Glacial Maximum and Terminal Pleistocene: a geospatial analysis of the 25–12 ka Australian archaeological record. *J. Archaeol. Sci.* **40**, 4612-4625, doi:10.1016/j.jas.2013.06.015 (2013).
30 Williams, A. N. A new population curve for prehistoric Australia. *Proc. R. Soc. Lond. B* **280**, 20130486 (2013).
31 Hope, J. & Hope, G. in *The Origin of the Australians* (eds R.L. Kirk & A.G. Thorne) 29-54 (Institute of Aboriginal Studies, 1976).
32 Hempson, G. P., Archibald, S. & Bond, W. J. A continent-wide assessment of the form and intensity of large mammal herbivory in Africa. *Science* **350**, 1056, doi:10.1126/science.aac7978 (2015).
33 Tallavaara, M., Eronen, J. T. & Luoto, M. Productivity, biodiversity, and pathogens influence the global hunter-gatherer population density. *Proc. Natl. Acad. Sci. U.S.A.* **115**, 1232-1237, doi:10.1073/pnas.1715638115 (2018).
34 Finlayson, C. *et al.* The *Homo* habitat niche: using the avian fossil record to depict ecological characteristics of Palaeolithic Eurasian hominins. *Quat. Sci. Rev.* **30**, 1525-1532, doi:10.1016/j.quascirev.2011.01.010 (2011).
35 Frankham, R., Bradshaw, C. J. A. & Brook, B. W. Genetics in conservation management: revised recommendations for the 50/500 rules, Red List criteria and population viability analyses. *Biol. Conserv.* **170**, 56-63, doi:10.1016/j.biocon.2013.12.036 (2014).
36 Frankham, R. Effective population size/adult population size ratios in wildlife: a review. *Genet. Res.* **66**, 95-107, doi:10.1017/S0016672300034455 (1995).
37 Amrhein, V., Greenland, S. & McShane, B. Scientists rise up against statistical significance. *Nature* **567**, 305-307, doi:10.1038/d41586-019-00857-9 (2019).
38 Bronk Ramsey, C. Bayesian analysis of radiocarbon dates. *Radiocarbon* **51**, 337-360, doi:10.1017/S0033822200033865 (2009).
39 Bronk Ramsey, C. Deposition models for chronological records. *Quat. Sci. Rev.* **27**, 42-60, doi:10.1016/j.quascirev.2007.01.019 (2008).
40 Timmermann, A. & Friedrich, T. Late Pleistocene climate drivers of early human migration. *Nature* **538**, 92-95, doi:10.1038/nature19365 (2016).
41 Williams, A. N., Ulm, S., Sapienza, T., Lewis, S. & Turney, C. S. M. Sea-level change and demography during the last glacial termination and early Holocene across the Australian continent. *Quat. Sci. Rev.* **182**, 144-154, doi:10.1016/j.quascirev.2017.11.030 (2018).
42 Prowse, T. A. A. *et al.* An efficient protocol for the global sensitivity analysis of stochastic ecological models. *Ecosphere* **7**, e01238, doi:10.1002/ecs2.1238 (2016).
43 Drechsler, M. Sensitivity analysis of complex models. *Biol. Conserv.* **86**, 401-412 (1998).
44 Wainwright, H. M., Finsterle, S., Jung, Y., Zhou, Q. & Birkholzer, J. T. Making sense of global sensitivity analyses. *Comput. Geosci.* **65**, 84-94, doi:10.1016/j.cageo.2013.06.006 (2014).

Reviewers' comments:

Reviewer #1 (Remarks to the Author):

I have re-read the paper and I find it to be a fine piece of scientific work. In my first review, I indicated that I was generally pleased with the article, though I left the modelling aspects to others (who I see have significant comments, though the rebuttal and revisions look strong). My main point was to have the archaeology speak more, and I also recommended that the figure on archaeology be moved from the supplementary section to the main text. This has been done, and I think that has improved the paper more, with the contribution of archaeology being more apparent.

Reviewer #4 (Remarks to the Author):

The paper presents the results of a cellular automaton model that uses ecological, ethnographic, climatological, and geographic parameters to simulate the dispersal of modern humans across Sahul. The aim of the paper is to evaluate an array of possible entry routes and subsequent expansions across the continent that are then correlated with the archaeological record (which is itself corrected for observational bias).

The model supports an initial entry from the northwest shelf of Sahul followed by a second entry through New Guinea. The timing is consistent either with a recent entry of 50kyBP or an early entry of 75kyBP. The results also suggest a rapid expansion of humans across Sahul achieving a maximum possible population abundance of over 6 million.

The size and scope of the project is immense, and so I assume the work involved was equally immense. As I'm sure the authors are aware a project of this size is on the one hand impressive and on the other daunting.

In studies such as these where the emphasis is placed on sophisticated, complex computation rather than the analysis of data, for example, the reader (and reviewer) must have faith that the results presented have robust computational support. This is even more relevant here as these results, while replicable in theory I'm sure, cannot be replicated without access to a supercomputer and a team of specialists. This in itself is not a problem, just a statement of fact.

So, the question is how convinced is the reader (and reviewer) that the results as presented are both robust and supported by the computation. To begin, it is reasonably safe to proceed under the assumption that the computation was done correctly and thoroughly by people with the relevant expertise, otherwise they wouldn't have undertaken a project like this. As such, the following comments are not about the computational ability of the authors, but the computation itself and the issues that arise.

Sensitivity. As noted by previous reviewers a major issue in this kind of project is sensitivity. I can't begin to count the number of parameters and their interactions, all of which must be estimated from data and the results of previous work. Clearly, every parameter is estimated individually in some way, and unavoidably all will involve varying amounts of statistical error. I have no reasonable suggestion of how to untangle the sensitivity of the results to variation in the value of parameters, let alone their interactions and their functional forms. The authors run a boosted-regression model in response to similar observations from previous reviews, but of course, the cellular automaton model is not a boosted-regression model and so how the results of one relate to the other are uncertain. Perhaps this is all that can be done as a conventional sensitivity analysis is not possible here. As such, I would like to see an acknowledgment of this limitation in the paper and some discussion of the fact that all these parameters and the functional forms of their response are simply models. The reason that this is important is that you want to avoid condescending the reader by implying this model is too

complicated for the average reader to understand, and trust us, it works.

Results. Again, as raised by previous reviewers, the primary question I have about the results is the population density and abundance estimates. I found the response to the very reasonable questions raised by the previous reviewers to be unconvincing. Extraordinary claims require extraordinary evidence etc. The authors suggest these estimates are maxima, but I don't see how they are maxima in the statistical sense; they are simply the results of the model at saturation given the parameters. If in some way the same model was to reduce the abundance estimate the timing of saturation would necessarily change, altering the results, as implied by the boosted-regression sensitivity.

The actual issue here is that the 6+ million abundance estimate is so large that the worry is specialists will then question the validity of the entire model, as this number is about an order of magnitude greater than other estimates. This then requires much explanation, which may indeed be possible. But it will be hard for readers to be convinced by one set of results while being skeptical of the others. Maybe deemphasize the population results in favor of the speed of colonization. Out of interest, the average densities proposed for late Pleistocene Australia (0.5+ N/ km2) are in the upper half of the distribution of ethnographic observations (i.e., central African tropical foragers), which is surprising, and then whether these estimates are uniformly valid across the entire landscape is questionable too. My hunch is that there's something in the modeling of interactions among hunter-gatherer population densities, carrying capacities, and environments which is being missed, or averaged over in some way.

A related issue is that I don't understand the population density modeling or approach. There is now quite a reasonable literature on hunter-gatherer population densities, their environmental dependence and how this is related to ecological principles in general. I don't follow why carrying capacities are modeled as parabolic with environmental productivity, or quadratic. Moreover, I don't follow Figures S2b, c, and d. In Figure S2b, why does population density decrease with K? With higher carrying capacities you find lower population densities? Isn't that opposite to the ecological definition of a carrying capacity? Similarly, in Figure S2c, if there's a parabolic relationship between K and P, why does population density increase with K? And how do these relationships result in the maps below (e-g)? For example, Figures S2b and c show almost the inverse relationships between density and K, so why are the maps similar (generally speaking)? There's something there I'm missing. I can't match up how the spatial distributions emerge from the 1d distributions.

I also agree with reviewer #2 in their comment about the low Spearman's ρ scores. Regardless of arbitrary levels of statistical significance, ρ can vary between -1 and 1, and within that range there are strong and weak correlations. A ρ of 0.12+/- is not a strong correlation regardless of the nature of the data. In their response #18 they say this is remarkably high given the uncertainty in the parameters and the nature of the archaeological data, but in this sense, they are arguing that it is remarkable that there is a non-random signal at all. This may be true, but again, it is hard to argue this is a strong result, and this is why these results are in the SI, not the main text. This raises the more important point that even though they do not use the archaeological record as "validation", but "congruence", statistically speaking the archaeological record is being used as validation as it is the only link between the computation and the real world. The problem here I suspect is that there is little to no significant spatial gradient in the radiocarbon ages of early Australian sites to empirically model the expansion across the continent (and that's why this result is not presented). If there is no signal in the validation (or congruence) layer then the spatial distribution of site ages is random, in which case I'm not sure what the expectation of this correlation would be. Perhaps this is what they mean by claiming this is in someway a remarkable result. If so, this logic needs to be clarified further. However, statistically, you cannot turn a weak result into a strong result by claiming special circumstances. A low ρ by definition means that there will be a large number of alternative hypotheses that could generate this correlation. The onus is then on the authors to explain why this weak correlation supports their model (which is entirely plausible).

Discussion. The first half of the discussion is very interesting in terms of what this model means for understanding the colonization of Australia, though it is unclear in places how much some of the "results" are simply the result of the parameters fed into the model (i.e., "baked in" to use the other reviewer's phrase), and this is an important point that readers will wonder about. I found the second half of the discussion to be underwhelming and not well thought out. In the second half (line 379 on) there are a few claims about what this model means for the ability of humans to adapt to a diversity of habitats (line 381) and the complexity of human technological adaptations (line 395). Neither the model, nor the modeling approach says anything about these issues. The model is a sophisticated CA that runs given a certain number of inputs and generates some simulated output. It says nothing about human adaptation, or flexibility, or diversity etc. Please rethink these claims and makes sure archaeologists on the team review this section. Similarly, the last claim in the main text (line 404) suggests the large assemblage at Madjedbebe is consistent with a large regional population size; this statement makes little archaeological sense without further reasoning.

In sum, this is an interesting and impressive paper, but given the scale of the approach there are many details a specialist will want to see discussed further.

Please see the attached pdf for specific comments on the text.

Revision Summary

Following our appeal where we were invited to submit a revised version in response to Reviewer #4, we reply to each of the reviewer's comments in detail below and detail how we revised the manuscript accordingly. We also specifically respond to the Associate Editor's three main requests:

- we have now clarified any possible misunderstandings in the manuscript;
- we have added caveats to some of the original claims we made about population size, which proved a key issue for several of the reviewers. This includes removing the text describing the population-size estimates from the Abstract, and focussing on the main outputs of the model around the speed and directionality of the peopling of the continent;
- we changed the title to reflect more accurately some of the uncertainty inherent in our approach — We had changed the original title from ***Stochastic models reveal rapid early peopling of Late Pleistocene Sahul*** (submitted version) to ***Stochastic models reveal ~~rapid~~ early peopling of Late Pleistocene Sahul*** (R1 version) in response to Reviewer #3 in the first round of reviews (our Response #13 in our first response to reviewers). In that response, we aquiesced regarding the ambiguity of an early (~ 75 ka) *versus* later (~ 50 ka) entry and removed the word 'rapid' from the title, but we defended our use of the word 'reveal'. Upon reflection and following the Associate Editor's advice, as well as acknowledging Reviewer #4's scepticism, we have now modified the title to ***Stochastic models support rapid peopling of Late Pleistocene Sahul***. This new title de-emphasises the 'revelation' claim, and focuses on the main, uncontested result — the rapidity of continental peopling irrespective of assumptions about the carrying capacity of ancient human societies (see also Response #4 below).

Reviewer #4

The paper presents the results of a cellular automaton model that uses ecological, ethnographic, climatological, and geographic parameters to simulate the dispersal of modern humans across Sahul. The aim of the paper is to evaluate an array of possible entry routes and subsequent expansions across the continent that are then correlated with the archaeological record (which is itself corrected for observational bias). The model supports an initial entry from the northwest shelf of Sahul followed by a second entry through New Guinea. The timing is consistent either with a recent entry of 50kyBP or an early entry of 75kyBP. The results also suggest a rapid expansion of humans across Sahul achieving a maximum possible population abundance of over 6 million. The size and scope of the project is immense, and so I assume the work involved was equally immense. As I'm sure the authors are aware a project of this size is on the one hand impressive and on the other daunting.

In studies such as these where the emphasis is placed on sophisticated, complex computation rather than the analysis of data, for example, the reader (and reviewer) must have faith that the results presented have robust computational support. This is even more relevant here as these results, while replicable in theory I'm sure, cannot be replicated without access to a supercomputer and a team of specialists. This in itself is not a problem, just a statement of fact.

RESPONSE #1: (**a**) The reviewer's implies that modelling is inferior to "analysis", when in fact without modelling complex systems we would not have any idea of the magnitude of future climate change, human population growth, expected biodiversity extinction rates, required energy transitions, or agricultural trends over the next century. Complex-systems

models are therefore an integral part of modern science today. That we can apply complex-systems models to investigate the past (hindcasting rather than forecasting) and use data from several different disciplines represent an advance, not a weakness. We have therefore added the following text (added/modified text in red below) in the third paragraph of the Introduction to clarify this aspect:

> "To address this large gap in our understanding, we constructed a fully stochastic, cellular-automaton model at a resolution of 0.5° × 0.5° to quantify how the patterns of peopling of Sahul could have occurred based on measured ecological mechanisms. Stochastic, complex-system models like the one we present here are useful not only for forecasting phenomena like global and regional climate patterns[1], biodiversity extinctions[2], and land use[3], but also for hindcasting past phenomena based on hypothesised, but hitherto untested mechanisms[4,5]. Our model incorporates … "

(**b**) The reviewer seems to imply that we did not analyse data. This is incorrect. Data analyses are at the core of every single element of the model, as well as the congruency analysis (more on this below in Responses #8–9). For example, we:

- **analyse data on human demography**[6] within a base population model[7],
- **analyse climate data** hindcasted using a global circulation model[5,7,8] to predict spatially explicit and temporally variable human carrying capacities in the form of densities (derived from **analyses of data on hunter-gatherer densities**[9]),
- incorporate and **analyse data from several sources**[10-12] **to build fissioning and dispersal** functions that depend on our **original analyses of data on territory size fluctuating as a function of rainfall**[13] and **landscape ruggedness**[14] (that we analysed and computed with even more data[15]).

Even the functions we assumed are based on strong theoretical support and other data sources, including:

- our spatially clustered catastrophe function (based on real data for vertebrates[16]),
- water limitation for dispersal[17,18], and
- the productivity-carrying capacity relationship for humans[9,19,20].

Not only is our model itself based on these many data analyses, the **congruency analysis is an entirely novel analysis of archaeological data** developed specifically for this paper using an advanced technique to remove biases.

We have therefore added/modified (changes in red) the following text in the third paragraph of the Introduction:

> "Our model and analyses incorporate data-based human-population dynamics to project cell-specific population densities relative to hindcasted[5], climate-dependent[21] predictions of maximum carrying capacity derived from established relationships between hunter-gatherer densities and environmental conditions[9], data-based rules of migration between neighbouring cells, measured long-distance dispersal[12,22-24] contingent on water availability[17] and landscape ruggedness[25] (a proxy for accessibility), as well as spatially clustered catastrophic mortality events operating at a generational scale based on data from across vertebrate taxa[16].

We also changed the fifth paragraph of the Introduction to:

> "To rank the relative realism[26] of which timings of initial entry, point(s) of initial entry, and carrying-capacity scenarios were best supported by the available archaeological data, we designed a novel analysis of the oldest-available, quality-rated archaeological dates to generate a map of initial arrival across the continent corrected for spatial bias and temporal uncertainty. We calculated the correspondence between this bias-corrected map and each of the 120 modelled scenarios to rank their relative likelihood given the archaeological data."

(**c**) The reviewer's states that "… while replicable in theory I'm sure, cannot be replicated without access to a supercomputer and a team of specialists". However, this is incorrect. All of the code and relevant data are freely available online and can be run on any personal desktop/laptop (there no need of a "supercomputer" — we used a high-performance computing facility of the type available to almost every university researcher in the world that simply renders the processing more efficient because of running simulations in parallel rather than in sequence).

To avoid such confusion, we have clarified this aspect in the first section of the Methods:

> "**Cellular-automaton framework**. We constructed the cellular-automaton model in the open-access R statistical computing environment (cran.rproject.org). We provide all code, data, and instructions to repeat the analysis at github.com/cjabradshaw/SahulHumanSpread, which can be run on any desktop computer. Our spatial model is based … "

There is therefore no requirement to take anything we present with any "faith", because every element is well-described, repeatable, with all data and open-access code available to anyone.

So, the question is how convinced is the reader (and reviewer) that the results as presented are both robust and supported by the computation. To begin, it is reasonably safe to proceed under the assumption that the computation was done correctly and thoroughly by people with the relevant expertise, otherwise they wouldn't have undertaken a project like this. As such, the following comments are not about the computational ability of the authors, but the computation itself and the issues that arise.

RESPONSE #2: Following Response #1c, the reader is not required to make an "assumption" or take anything on "faith".

Sensitivity. As noted by previous reviewers a major issue in this kind of project is sensitivity. I can't begin to count the number of parameters and their interactions, all of which must be estimated from data and the results of previous work. Clearly, every parameter is estimated individually in some way, and unavoidably all will involve varying amounts of statistical error. I have no reasonable suggestion of how to untangle the sensitivity of the results to variation in the value of parameters, let alone their interactions and their functional forms. The authors run a boosted-regression model in response to similar observations from previous reviews, but of course, the cellular automaton model is not a boosted-regression model and so how the results of one relate to the other are uncertain. Perhaps this is all that can be done as a conventional sensitivity analysis is not possible here. As such, I would like to see an acknowledgment of this limitation in the paper and some discussion of the fact that all these parameters and the functional forms of their response are simply models. The

reason that this is important is that you want to avoid condescending the reader by implying this model is too complicated for the average reader to understand, and trust us, it works.

RESPONSE #3: All the parameters in the model are clearly specified both in the text and the open-access R code. We explain each and every parameter, its derivation, its calculation, and most importantly in the context of this comment, the influence of each parameter on the model predictions (Figure 5). The reviewer might not be familiar with the well-established and empirically demonstrated strength of global sensitivity analyses[27] that we used to take all uncertainty into account, including all parameter uncertainty, interactions, dependencies and feedbacks.

We never claimed that "… the cellular automaton model is … a boosted-regression model …". We explicitly stated in the *Global sensitivity analysis* section of the Methods that it is the emergent *result* of the model (time to saturation) that we use in a second analysis (boosted regression tree of the Latin hypercube-sampled parameter volume) as a response variable to see how variation in the underlying parameters affect it. Thus, the cellular-automaton spatial model is run 1000 times, varying the parameters within the ranges as indicated, and then variation in the outcome (time to continental saturation) is then explained by the relevant variation in the input parameter values. This is the essential process of all global sensitivity analyses (a synposis that the lead author co-wrote explaining how global sensitivity analyses are done is available open-access here[27]; we also cite that paper in the manuscript) used across the sciences, and we explicitly provide all the code so that others can repeat our analyses. We never implied that the model is "… too complicated for the average reader to understand …".

To remove any ambiguity about our description of this approach, we have modified the relevant section in the Methods (first two paragraphs):

> "For this global sensitivity analysis, we used the initial scenario parameters of a 50-ka entry at the southern route, the rotated parabolic relationship between K and P_p, and assuming a founding population size stochastically sampled between 1300 and 1500 people for the entry point[7].
>
> Here, we ran the cellular-automaton spatial model 1000 times, randomly sampling 12 of its parameters uniformly for each iteration based on a Latin hypercube-sampling protocol[27]. We set the 12 parameters to be sampled with ± 50% variation on the median value used in the model (except for N_{cat} and max N/K with a maximum upper bound of 0.99, and for maximum D_{cell} — see below); these 12 parameters were: (*i*) …"

and (last paragraph):

> "We chose to summarise the output of each of these 1000 parameter-sampled runs of the spatial model as the time taken to achieve continental saturation (i.e., the number of years taken from initial entry to occupy every cell in Sahul). In a separate analysis, we then tested the influence of the per-model run parameter values (predictors) on the time to continental saturation (response) using a boosted-regression tree[28] emulator with the function *gbm.step* in the `dismo` R library[29]. Here, we set the error distribution family as Gaussian, the bag fraction to 0.75, the learning rate to 0.008, the tolerance to 0.0001, the maximum number of trees to 10,000, and the tree complexity to 2 (first-order interactions only). To assess the relative contribution of each of the 12 randomly sampled parameters to the time to spatial saturation, we calculated the boosted-regression tree metrics of relative influence[27]."

Results. Again, as raised by previous reviewers, the primary question I have about the results is the population density and abundance estimates. I found the response to the very reasonable questions raised by the previous reviewers to be unconvincing. Extraordinary claims require extraordinary evidence etc. The authors suggest these estimates are maxima, but I don't see how they are maxima in the statistical sense; they are simply the results of the model at saturation given the parameters. If in some way the same model was to reduce the abundance estimate the timing of saturation would necessarily change, altering the results, as implied by the boosted-regression sensitivity.

RESPONSE #4: We did not claim that the carrying-capacity estimates (which are derived from real data[9], contrary to what the reviewer suggests) were statistical maxima. Instead, they are 'maxima' in the sense of what *carrying capacity* means: "… some upper limit to total abundance"[30], which is expressed mathematically as the point at which net population growth becomes stable ($r = 0$)[31,32]. It is therefore an ecological concept, not a statistical one. We have clarified this by adding the following sentence in the 'Carrying capacity' section of the Methods:

> "In this case, we define *carrying capacity* as the upper limit to total abundance[30], which is expressed mathematically as the point at which net population growth becomes stable ($r = 0$)[31,32]."

More importantly, the reviewer's statement that the carrying capacity influences time to saturation is incorrect. The parameters *maximum and minimum N/K* and *maximum population growth rate* (r_m) — all parameters that depend on carrying capacity (K) — have almost no influence on the time to saturation (Figure 5).

We have clarified this in the first paragraph of the Discussion:

> "… and temperate forests. This result was largely insensitive to assumptions regarding landscape carrying capacity (minimum and maximum ratios of N/K per cell invoking an emigration, and maximum population growth) (Fig. 5). Based on …"

The actual issue here is that the 6+ million abundance estimate is so large that the worry is specialists will then question the validity of the entire model, as this number is about an order of magnitude greater than other estimates. This then requires much explanation, which may indeed be possible. But it will be hard for readers to be convinced by one set of results while being skeptical of the others. Maybe deemphasize the population results in favor of the speed of colonization. Out of interest, the average densities proposed for late Pleistocene Australia (0.5+ N/ km2) are in the upper half of the distribution of ethnographic observations (i.e., central African tropical foragers), which is surprising, and then whether these estimates are uniformly valid across the entire landscape is questionable too. My hunch is that there's something in the modeling of interactions among hunter-gatherer population densities, carrying capacities, and environments which is being missed, or averaged over in some way.

RESPONSE #5: There are no pre-existing estimates of human density for Sahul apart from those co-author Alan Williams and colleagues[33] constructed from archaeological specimen densities. While that research explored the initial peopling of Australia, it was hampered by a lack of data during this period, did not account for arrival potentially as early as ~ 65,000 years ago, and ultimately focussed on periods much later than the one we are modelling here. While there are many assumptions and debates about the approach

used in that study, there is nothing in that work that invalidates our estimates because ours are based on ecological principles grounded in real data.

As we clearly stated, previous estimates have not included New Guinea — a region of some of the highest productivities in all of Sahul — as do ours, so our values will necessarily be higher than any previous estimates of population size. Further our estimates also include large parts of the sunken coastal shelf (~ 1.43 million km^2)[34] — an area that is 19% larger than today's Australia, with much of it potentially more productive than extant continental Australia. None of the existing studies has considered this before. Our landmass-based model will therefore produce higher estimates than any of those previously attempted. It is also essential to recall that smallpox and other European diseases devastated *in situ* Aboriginal populations at the time of colonisation, so our estimates of pre-contact population size are likely to be downwardly biased.

To demonstrate this aspect further, we overlaid the grid of modern-day Australia + New Guinea to the population layer for the 50 ka-entry (as in Fig. 3) at continental saturation. We have added this overlay as an inset in a revised version of Figure 3 (reproduced below). If we add up the population values for the grid cells that are now underwater, this gives 1.78 million people, or 27.6% of the population estimate expressed in Fig. 3 ('para'). If we look at just the Australian component of these estimates (again, based on the modern-day extent of the Australian grid), the underwater cells predict a total of 1.26 million people, or 23% of the total population estimate for the Australian component of Sahul. This means that if we just look at the above-water land cells in the Australian part of Sahul only, the total predicted population size would be 3.1 million people. The 'missing' area to which our estimates apply therefore account for much of the discrepancy between previous estimates and those predicted from our model.

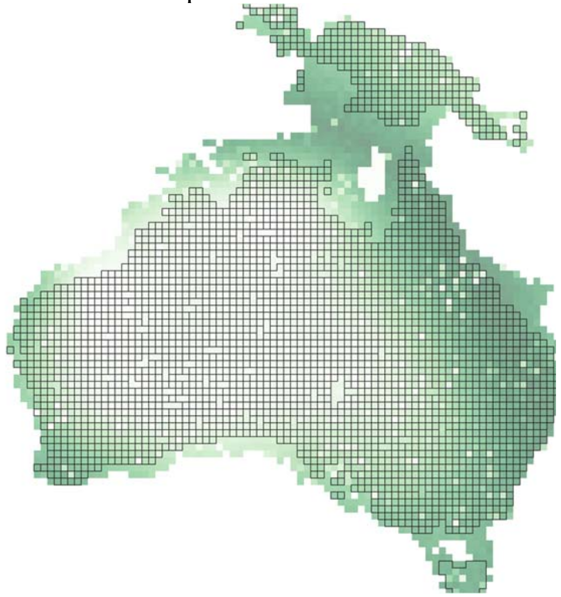

New inset figure (Fig. 3d) showing the overlay of the modern-day Australia/New Guinea grid on the maximum extent of Sahul.

Also as we previously stated, our density estimates are well within the range of existing ethnographic observations of human density, and so have ecological validity. There is no existing evidence that supports the notion that ancient humans were incapable of achieving the same densities as hunter-gather societies today (this is an untested assumption). This, coupled with the recently published paper by De Deckker and colleagues[35] that during the period of likely human entry into Sahul, the intertropical convergence zone shifted south,

resulting in relatively wetter conditions in northern Australia that likely increased local carrying capacity on the extended continental shelf.

We do not state or otherwise imply that "… these estimates are uniformly valid across the entire landscape …". The densities vary in both time and space according to the predictions from the empirically supported climate and human density models. Nor does the reader require any "hunch", because we justify the model parameters, we explicitly and empirically outline all interactions, nor did we do any averaging or apply otherwise hidden functions. Finally, despite the well-supported model outputs regarding density and population size, we clearly state that this is not a 'prediction' of what actual densities were as such, and that the most important component of the model is the pace and spatial pattern of dispersal.

However, given the persistent resistance to our presentation, characterisation, and qualification of the population estimates, we have radically toned down the relevant sections of the manuscript, and provide additional caveats regarding the interpretation of this result. First, we added the following to the Results where we first mention the population estimate:

> "These values are considerably greater than previous estimates[33], and so we provide several caveats regarding the interpretation of this result in the Discussion."

Next, we have completely modified the relevant section of the Discussion as follows:

> "The highest-ranked relationship between carrying capacity and net primary production predicted a maximum continental-wide population of 6.3–6.5 million (0.55–0.57 people km^{-2}) based on the carrying capacity predicted from the LOVECLIM Earth-systems model and the particular form of the best-supported relationship between human population density and net primary production we considered. One of the main determinants of this range — which is substantially larger than previous reconstructions of population size for Late Pleistocene Sahul[33] — is the relatively higher support for the non-linear relationship (rotated parabolic) between human carrying capacity and net primary production compared to the other two forms. Although a non-linear relationship is consistent with evidence from elsewhere that ancient people selected habitats that were structurally intermediate (ecotones) between open plains and dense forests (e.g., savannas)[20], thus providing vegetation mosaics that were more ecologically diverse and favourable to human visitation and settlement than maximum-productivity environments[19], we did not have empirical data to support the exact form of that relationship. Testing the *relative* support among three different types of relationships does not mean that the rotated parabolic is in fact the correct one; instead, it merely gives more support for the hypothesis of non-linearity *versus* linearity. The combination of the spatial pattern of net primary production across Sahul (Supplementary Fig. S2) and the latter's relationship with human density drives the magnitude of the total population estimate (Fig. 3). Without a specific, empirically supported relationship, it is entirely plausible that the total population size could have been substantially smaller, especially if other, unknown conditions prevented populations from achieving local carrying capacity. The true magnitude of the population estimate is therefore not central to our approach or the ensuing results.
>
> Nonetheless, the large estimate and the resulting population-density range are not at odds with post-contact estimates of hunter-gatherer densities across temperate coastal regions of Australia (mean ± SD: 0.34 ± 0.24 people km^{-2}, with an upper limit of 0.81 people km^{-2} assuming a Gaussian distribution) and densities of shifting agriculturalists

in New Guinea (4.37 ± 4.22 people km^{-2})[36]. Additionally, the only existing estimates of population size in Sahul[33] did not account for arrival before 50 ka, and instead focussed on periods much later than the arrival window we considered. Neither did those estimates include New Guinea, which represents ~ 15% of the Sahul landmass at during the period of interest (and contains areas of higher ecological productivity than much of the remainder of Sahul), nor did they include the sunken coastal shelf (~ 1.43 million km^2, or ~ 19% more land area than Australia today). Overlaying the modern extent of Australia and New Guinea on the predicted population grid at 300 generations from initial entry indicates that approximately 27.5% of the continent's total population is attributed to cells that are now under water (Fig. 3). In the case of the 50 ka single-entry scenario shown in Fig. 3, the above-water component of the Australian part of Sahul would give a total population of 3.1 million people at saturation."

It is important to highlight that we have elected not to remove this result because it is an emergent outcome of the model parameters that is needed to understand some of the other outputs. Instead, we de-emphasise the result in terms of the underlying assumptions leading to its magnitude and any untested assertions of its interpretation.

A related issue is that I don't understand the population density modeling or approach. There is now quite a reasonable literature on hunter-gatherer population densities, their environmental dependence and how this is related to ecological principles in general. I don't follow why carrying capacities are modeled as parabolic with environmental productivity, or quadratic.

RESPONSE #6: We disagree. There is actually very little information on the relationship between primary production and density for humans, and we have cited all relevant papers[19,20] (including those covering non-human species[37]). We have also cited the authoritative paper[9] on the relationships between hunter-gatherer population densities and various environmental variables, from which we directly derived the carrying capacities across the landscape. We did not model carrying capacities according to these hypothetical relationships — as we stated in the manucript. Instead, we modelled the hypothetical relationships between human densities and net primary production. This was necessary to construct empirically based (from climate hindcasts) estimates of human carrying capacity.

As explained in Response #5, because there is just the mere suggestion of evidence for a nonlinear relationship between these two measures in previous work[19,20], and no empirical relationship *per se*, we were obliged to test two generalised nonlinear relationships (the rotated parabolic and quadratic yield density [not "quadratic"]) against a simple linear model. The exact form of the nonlinear models is not the point here, it is whether a nonlinear model outperforms a linear one, which we have demonstrated empirically with comparison to the archaeological reconstruction map. The restructured Discussion given in Response #5 makes this clearer.

Moreover, I don't follow Figures S2b, c, and d. In Figure S2b, why does population density decrease with K? With higher carrying capacities you find lower population densities? Isn't that opposite to the ecological definition of a carrying capacity? Similarly, in Figure S2c, if there's a parabolic relationship between K and P, why does population density increase with K? And how do these relationships result in the maps below (e-g)? For example, Figures S2b and c show almost the inverse relationships between density and K, so why are the maps

similar (generally speaking)? There's something there I'm missing. I can't match up how the spatial distributions emerge from the 1d distributions.

RESPONSE #7: This is a simple misunderstanding that can be easily rectified. The 'densities' in Figures S2b-d are not human population densities, they are probability densities derived from the frequency histograms of cell-based estimates of carrying capacity (*K*). Here, the '*K*' estimates on the *x*-axis are human population densities (maximal, as explained in Response #5), and the *y*-axis label is the *probability* density (i.e., frequencies expressed as probability densities, where they sum to 1). This is a common way to show frequency histograms and a standard statistical convention. We have now clarified this by adding 'probability' in front of 'density' for the *y*-axis label in Figure S2b, and modified the caption accordingly:

> "**Fig. S2** (a) Assumed relationships between LOVECLIM-hindcasted net primary production and human carrying capacity (*K*, individuals per cell): linear, rotated parabolic, and reciprocal quadratic yield density. The probability density distribution (frequency histogram standardised so the values sum to 1) of cell-specific carrying capacities …"

I also agree with reviewer #2 in their comment about the low Spearman's ρ *scores. Regardless of arbitrary levels of statistical significance,* ρ *can vary between -1 and 1, and within that range there are strong and weak correlations. A* ρ *of 0.12+/- is not a strong correlation regardless of the nature of the data. In their response #18 they say this is remarkably high given the uncertainty in the parameters and the nature of the archaeological data, but in this sense, they are arguing that it is remarkable that there is a non-random signal at all. This may be true, but again, it is hard to argue this is a strong result, and this is why these results are in the SI, not the main text.*

RESPONSE #8: The main results here are NOT in the SI, they are expressed in Fig. 2 as the difference in correlation coefficients between models. The actual values, as we argued in the first revision, are not the central metric of the congruence analysis. What is important here is the relative change among the scenarios.

Indeed, if one were doing a simple bivariate correlation between *y* and *x*, then one could conclude that the correlation of 0.12 is not "strong" (even if statistically the relationship is supported). However, we are not doing simple bivariate correlations in this paper. We have a fully stochastic model built from real data combined into functions that capture human behavioural phenomena as they respond to temporal and spatial variability in environmental carrying capacity. Comparing the emergent property of these many different processes (peopling of cells progressively over time) to an equally uncertain expression of actual human presence derived from the archaeological data (corrected for spatial and preservation biases) means that we cannot possibly expect that these two layers (model-predicted peopling *versus* estimated timing of human presence) would ever approach "strong" correlations one might see in a simple bivariate relationship, especially across nearly 4000 cells. This is exactly why we are not doing a "validation" as such and instead a "congruency" (we elaborate and defend the use of our terminology for good reasons below in Response #9). To whit, it is incorrect to claim that the strength of the relationship was not "strong".

To address this issue in the manuscript, we have added the following text to the *Spatial correspondence between modelled and archaeologically derived dates of initial arrival* section of the Supplementary Information:

"It is important to understand that the actual value of ρ is not the central metric of the congruency analysis. What is instead important is the relative change in ρ among the scenarios tested. The fully stochastic model built from real data combined into functions that capture human behavioural phenomena as they respond to temporal and spatial variability in environmental carrying capacity cannot be expected to return high correlation coefficients as might be expected from a simple bivariate analysis. Comparing the emergent property of these many different processes (peopling of cells progressively over time) to an equally uncertain expression of actual human presence derived from the archaeological data (corrected for spatial and preservation biases) necessarily implies lower expected correlation, especially across nearly 4000 cells. Therefore, the comparison is not a validation as such, but instead a congruency. A validation requires a set of known (observed) attributes used to 'validate' the predictions of a model. In the current case, however, the archaeological data are not measured observations of a phenomenon — they are themselves subject to extensive uncertainty, including but not restricted to dating uncertainty, contamination, preservation bias, and sampling bias. They cannot therefore be used to validate the model predictions. Compiling these archaeological dates into a single layer incorporating both their inherent biases and uncertainties, in addition to the real (and often ignored[38]) spatial biases they create, means that the resultant archaeological date layer is itself a modelled output. We can, however, examine congruency between that layer and the model predictions to demonstrate which of the scenarios tested is (more) ecologically realistic."

The reviewer is perhaps a little more correct in claiming that our congruency analysis demonstrates non-random behaviour, but this belies the fact that the model is already non-random because the behavioural rules are governed by functions with appropriate embedded uncertainty. That there is any congruency (correlation) at all, and of the magnitude shown, is a remarkable indication of the model's ecological reality.

This raises the more important point that even though they do not use the archaeological record as "validation", but "congruence", statistically speaking the archaeological record is being used as validation as it is the only link between the computation and the real world. The problem here I suspect is that there is little to no significant spatial gradient in the radiocarbon ages of early Australian sites to empirically model the expansion across the continent (and that's why this result is not presented). If there is no signal in the validation (or congruence) layer then the spatial distribution of site ages is random, in which case I'm not sure what the expectation of this correlation would be. Perhaps this is what they mean by claiming this is in someway a remarkable result. If so, this logic needs to be clarified further.

RESPONSE #9: A validation is made when a set of **known** attributes is used to **validate** the predictions of a model. For example, a global circulation (climate) model is 'validated' by comparing measured climatic variables (e.g., **observed** maximum temperature, precipitation) to the **predictions** hindcasted from the model. If the correlation is low, then one can update the model's parameters to get closer to the observed values.

We are not dealing with an analogous system here, in that the archaeological data are not 'measured observations' of a phenomenon, so they cannot be used to 'validate' the model. The archaeological data are themselves subject to extensive uncertainty, including

but not restricted to dating uncertainty, contamination, preservation bias, and sampling bias. Further, to pull these archaeological dates into a single layer incorporating both their inherent biases and uncertainties, in addition to the very real (and nearly always ignored[38]) spatial biases they create, means the resultant archaeological date layer is itself a modelled output. It cannot therefore be used to 'validate' the model. We can, however, examine congruence to show that the model is ecologically realistic. The added text described in Response #8 addresses this issue in the Supplementary Information.

However, statistically, you cannot turn a weak result into a strong result by claiming special circumstances. A low ρ by definition means that there will be a large number of alternative hypotheses that could generate this correlation. The onus is then on the authors to explain why this weak correlation supports their model (which is entirely plausible).

RESPONSE #10: We are not claiming anything of the sort, nor are we arguing "special circumstances". We have already explained the approach in detail, and outlined the limitations, the assumptions, and the uncertainties involved as described in the previous responses and modified/added text to the manuscript.

Discussion. The first half of the discussion is very interesting in terms of what this model means for understanding the colonization of Australia, though it is unclear in places how much some of the "results" are simply the result of the parameters fed into the model (i.e., "baked in" to use the other reviewer's phrase), and this is an important point that readers will wonder about.

RESPONSE #11: Our previous response should be sufficient to address this unsupported claim of 'baked in', but we elaborate more here. We contend that in a temporally and spatially dynamic model where all parameters are stochastically resampled, nothing is "baked" in. This is not how such models function. There is nothing deterministic about our model, so one could never predict an outcome based on a single (e.g., median) value for any particular parameter.

For example, each cell has a hindcasted net primary production confidence range that changes through time, from which each iteration samples randomly. Nothing is constant over space or time. When translated to carrying capacities, these sampled ranges are carried over into expressions of maximum human densities. Then, the demographic and dispersal components are also derived from resampling probability distributions. Therefore, not a single component of this model can be predicted based on first principles — the emergent property of all these stochastic processes is exactly why such a model is needed to project any element of interest (spread rate, densities, etc.). No other study has ever come close to this degree of sophistication for modelling ancient human spread across any landscape. Furthermore, uncertainty is further derived from the carrying capacity ~ net primary production relationships (see Response #4), and this is an entirely novel addition to the model. In short, nothing is pre-supposed in our model.

The reviewer's suggestion to clarify this aspect for the reader is a good one, so we have added the following text to the end of the first paragraph of the Discussion:

"The complexity of this fully stochastic model also ensures that no single component — measured or otherwise assumed — can dominate the outcomes of each scenario run. The emergent property of the many interacting and stochastically sampled functions and parameters provide the net spatial patterns of peopling and the plausible pace of this process. No single function within the model could achieve the same outcomes."

I found the second half of the discussion to be underwhelming and not well thought out. In the second half (line 379 on) there are a few claims about what this model means for the ability of humans to adapt to a diversity of habitats (line 381) and the complexity of human technological adaptations (line 395). Neither the model, nor the modeling approach says anything about these issues. The model is a sophisticated CA that runs given a certain number of inputs and generates some simulated output. It says nothing about human adaptation, or flexibility, or diversity etc. Please rethink these claims and makes sure archaeologists on the team review this section.

RESPONSE #12: We respectfully disagree. While not necessarily a direct output of the model, indirectly the results can be combined with our broader understanding of hunter-gatherer behaviour to make inferences about what the model outputs could mean for the broader archaeological discipline, as we have done here. Our discussions are necessarily cautious, since we acknowledge that the model does not directly ouput such human behaviour. However, the result of rapid expansion and persistence in such variable (in many parts, environmentally hostile) regions of the Sahul megacontinent strongly implies advanced adaptability aided by technological prowess would have been required to achieve the results we observe; and this implication is important for the broader story of human peopling of the globe.We therefore provide a balanced discussion focussing on the model outputs, while making some suggestions as to what these might mean more broadly for others to explore further. We confirm that all the archaeologists on the manuscript — including some of the leading Australian researchers in the discipline — were involved in the development of this component of the manuscript.

Similarly, the last claim in the main text (line 404) suggests the large assemblage at Madjedbebe is consistent with a large regional population size; this statement makes little archaeological sense without further reasoning.

RESPONSE #13: The lower (Phase 2) assemblage at Madjedbebe contains tens of thousands of stone artefacts, over a thousand pieces of ground ochre, more than 400 grindstones and ground fragments, more than 30 ground-edge axes/fragments, and includes distinctive technologies not recovered from the overlying layers (e.g., discoidal cores, thinning flakes, convergent flakes, points). These totals come from a 2 × 2 m area and final sorting is not yet completed (hence numbers will increase). This density is greater than that seen for the Holocene layers at the site and the richness of cultural materials exceeds even that of most dense Holocene sites in Australia or New Guinea. It is on these grounds that we can support the claim that Madjedbebe was intensively occupied, supportive of the early high population densities predicted by our model.

We did not think it was necessary to elaborate here because the Madjedbebe site has been extensively published in *Nature*[39] and a range of other publications[40,41]. There are also several archaeological sites that include substantive evidence of human activity around 50,000 years ago, including Devils Lair (southwest Australia) and Boodie Cave (northwest Australia).

Given the reviewer's request for further reasoning, we have split the relevant paragraph of the Discussion into two, and added the following:

"The large assemblage of artefacts in the earliest phase of human occupation at Madjedbebe is also potentially consistent with a large population size[39]. For example, a 2 × 2 m area of the lower (Phase 2) assemblage at Madjedbebe contains tens of

thousands of stone artefacts, > 1000 pieces of ground ochre, > 400 grindstones and ground fragments, > 30 ground edge axes/fragments, and includes distinctive technologies not recovered from the overlying layers (e.g., discoidal cores, thinning flakes, convergent flakes, points)[39]. This density of artefacts is greater than that seen for the Holocene layers at the site and the richness of cultural materials exceeds even that of many dense Holocene sites in Australia and New Guinea[40,41]. These data support the idea that Madjedbebe was intensively occupied, supportive of the early high population densities predicted by our model. Additionally, recent evidence suggests that northern Sahul was relative wet during the period of human arrival as the intertropical convergence zone shifted south[61], potentially increasing productivity in this region. Improving and expanding the archaeological record in places of initial arrival and where population densities were highest, including now-drowned but previously productive coastal-shelf sites[42], must therefore form a research priority …" (note new citation[42] for underwater archaeology in Sahul).

However, we have now placed this new text immediately after the discussion of population-size caveats.

References (renumbered in this response; numbers do not correspond to reference numbers in the main text)

1 Rummukainen, M. State-of-the-art with regional climate models. *WIREs Climate Change* **1**, 82-96, doi:10.1002/wcc.8 (2010).

2 Strona, G. & Bradshaw, C. J. A. Co-extinctions annihilate planetary life during extreme environmental change. *Sci. Rep.* **8**, 16724, doi:10.1038/s41598-018-35068-1 (2018).

3 Dietzel, C. & Clarke, K. The effect of disaggregating land use categories in cellular automata during model calibration and forecasting. *Computers, Environment and Urban Systems* **30**, 78-101, doi:10.1016/j.compenvurbsys.2005.04.001 (2006).

4 Caley, T., Roche, D. M. & Renssen, H. Orbital Asian summer monsoon dynamics revealed using an isotope-enabled global climate model. *Nat. Comm.* **5**, 5371, doi:10.1038/ncomms6371 (2014).

5 Goosse, H. *et al.* Description of the Earth system model of intermediate complexity LOVECLIM version 1.2. *Geosci. Mod. Dev.* **3**, 603-633, doi:10.5194/gmd-3-603-2010 (2010).

6 Gurven, M. & Kaplan, H. Longevity among hunter-gatherers: a cross-cultural examination. *Pop. Dev. Rev.* **33**, 321-365, doi:10.1111/j.1728-4457.2007.00171.x (2007).

7 Bradshaw, C. J. A. *et al.* Minimum founding populations for the first peopling of Sahul. *Nat. Ecol. Evol.* **3**, 1057-1063, doi:10.1038/s41559-019-0902-6 (2019).

8 Bird, M. I. *et al.* Early human settlement of Sahul was not an accident. *Sci. Rep.* **9**, 8220, doi:10.1038/s41598-019-42946-9 (2019).

9 Tallavaara, M., Eronen, J. T. & Luoto, M. Productivity, biodiversity, and pathogens influence the global hunter-gatherer population density. *Proc. Natl. Acad. Sci. U.S.A.* **115**, 1232-1237, doi:10.1073/pnas.1715638115 (2018).

10 Birdsell, J. B. Some environmental and cultural factors influencing the structuring of Australian Aboriginal populations. *Am. Nat.* **87**, 171-207, doi:10.1086/281776 (1953).

11 McNiven, I. J. in *Australian Coastal Archaeology* (eds H.J. Hall & I.J. McNiven) 157-168 (ANH Publications, 1999).

12 Sutherland, G. D., Harestad, A. S., Price, K. & Lertzman, K. P. Scaling of natal dispersal distances in terrestrial birds and mammals. *Conserv. Ecol.* **4**, 1-16 (2000).

13 Hiscock, P. *Archaeology of Ancient Australia*. (Routledge, 2008).

14 Marwick, B., Johnson, A., White, D. & Eff, E. A. *binford*: Binford's Hunter-Gatherer Data, R package version 0.1.0 CRAN.R-project.org/package=binford (2016).

15 Riley, S. J., DeGloria, S. D. & Elliot, R. A terrain ruggedness index that quantifies topographic heterogeneity. *Intermount. J. Sci.* **5**, 23-27 (1999).

16 Reed, D. H., O'Grady, J. J., Ballou, J. D. & Frankham, R. The frequency and severity of catastrophic die-offs in vertebrates. *Anim. Conserv.* **6**, 109-114, doi:10.1017/S1367943003147 (2003).

17 Bird, M. I., O'Grady, D. & Ulm, S. Humans, water, and the colonization of Australia. *Proc. Natl. Acad. Sci. U.S.A.* **113**, 11477-11482 (2016).

18 Saltré, F., Chuine, I., Brewer, S. & Gaucherel, C. A phenomenological model without dispersal kernel to model species migration. *Ecol. Model.* **220**, 3546-3554, doi:10.1016/j.ecolmodel.2009.06.026 (2009).
19 Hope, J. & Hope, G. in *The Origin of the Australians* (eds R.L. Kirk & A.G. Thorne) 29-54 (Institute of Aboriginal Studies, 1976).
20 Finlayson, C. *et al.* The *Homo* habitat niche: using the avian fossil record to depict ecological characteristics of Palaeolithic Eurasian hominins. *Quat. Sci. Rev.* **30**, 1525-1532, doi:10.1016/j.quascirev.2011.01.010 (2011).
21 Eriksson, A. *et al.* Late Pleistocene climate change and the global expansion of anatomically modern humans. *Proc. Natl. Acad. Sci. U.S.A.* **109**, 16089, doi:10.1073/pnas.1209494109 (2012).
22 Gould, R. A. Puntutjarpa Rockshelter and the Australian desert culture. *Anthropol. Pap. Am. Mus. Nat. Hist.* **54**, 1-187 (1977).
23 Hewlett, B. S., van de Koppel, I. M. H. & Cavalli-Sforza, L. L. in *African Pygmies* (ed L. L. Cavalli-Sforza) 65-79 (Academic Press, 1986).
24 Fort, J., Pujol, T. & Cavalli-Sforza, L. L. Palaeolithic populations and waves of advance. *Cambridge Archaeol. J.* **14**, 53-61, doi:10.1017/S0959774304000046 (2004).
25 Binford, L. R. *Constructing Frames of Reference: An Analytical Method for Archaeological Theory Building Using Ethnographic and Environmental Data Sets*. (University of California Press, 2001).
26 Vahdati, A. R., Weissmann, J. D., Timmermann, A., Ponce de León, M. S. & Zollikofer, C. P. E. Drivers of Late Pleistocene human survival and dispersal: an agent-based modeling and machine learning approach. *Quat. Sci. Rev.* **221**, 105867, doi:10.1016/j.quascirev.2019.105867 (2019).
27 Prowse, T. A. A. *et al.* An efficient protocol for the global sensitivity analysis of stochastic ecological models. *Ecosphere* **7**, e01238, doi:10.1002/ecs2.1238 (2016).
28 Elith, J., Leathwick, J. R. & Hastie, T. A working guide to boosted regression trees. *J. Anim. Ecol.* **77**, 802-813, doi:10.1111/j.1365-2656.2008.01390.x (2008).
29 Hijmans, R. J., Phillips, S., Leathwick, J. & Elith, J. dismo: Species Distribution Modeling. R package version 1.1-4. CRAN.R-project.org/package=dismo. (2017).
30 Bradshaw, C. J. A. & Brook, B. W. in *Conservation Biology for All* (eds N.S. Sodhi & P.R. Ehrlich) 313-334 (Oxford University Press, 2010).
31 Bell, T. in *The Princeton Guide to Ecology* 239-246 (Princeton University Press, 2009).
32 Turchin, P. *Complex Population Dynamics: A Theoretical/Empirical Synthesis*. (Princeton University Press, 2003).
33 Williams, A. N. A new population curve for prehistoric Australia. *Proc. R. Soc. Lond. B* **280**, 20130486 (2013).
34 Williams, A. N., Ulm, S., Sapienza, T., Lewis, S. & Turney, C. S. M. Sea-level change and demography during the last glacial termination and early Holocene across the Australian continent. *Quat. Sci. Rev.* **182**, 144-154, doi:10.1016/j.quascirev.2017.11.030 (2018).
35 De Deckker, P. *et al.* Climatic evolution in the Australian region over the last 94 ka - spanning human occupancy -, and unveiling the Last Glacial Maximum. *Quat. Sci. Rev.* **249**, 106593, doi:10.1016/j.quascirev.2020.106593 (2020).
36 Lourandos, H. *Continent of Hunter-Gatherers: New Perspectives in Australian Prehistory*. (Cambridge University Press, 1997).
37 Hempson, G. P., Archibald, S. & Bond, W. J. A continent-wide assessment of the form and intensity of large mammal herbivory in Africa. *Science* **350**, 1056, doi:10.1126/science.aac7978 (2015).
38 Saltré, F. *et al.* Climate-human interaction associated with southeast Australian megafauna extinction patterns. *Nat. Comm.* **10**, 5311, doi:10.1038/s41467-019-13277-0 (2019).
39 Clarkson, C. *et al.* Human occupation of northern Australia by 65,000 years ago. *Nature* **547**, 306-310, doi:10.1038/nature22968 (2017).
40 Clarkson, C. *et al.* Reply to comments on Clarkson et al. (2017) 'Human occupation of northern Australia by 65,000 years ago'. *Aust. Archaeol.* **84**, 84-89, doi:10.1080/03122417.2018.1462884 (2018).
41 Clarkson, C. *et al.* The archaeology, chronology and stratigraphy of Madjedbebe (Malakunanja II): A site in northern Australia with early occupation. *J. Hum. Evol.* **83**, 46-64, doi:10.1016/j.jhevol.2015.03.014 (2015).
42 Benjamin, J. *et al.* Aboriginal artefacts on the continental shelf reveal ancient drowned cultural landscapes in northwest Australia. *PLoS One* **15**, e0233912, doi:10.1371/journal.pone.0233912 (2020).

Reviewer #4 (Remarks to the Author):

The edits provided by the authors have improved the manuscript and the responses to my previous questions were helpful on the most part. Other comments were dismissed.

The role of data analysis and how the computation was conducted has been clarified and I think this greatly helps the reader.

The issue of the 6 million + abundance estimate remains but is now downplayed. Nevertheless, I worry that this issue will detract from the other results in this paper.

Other than that I have no further comments.
